# An HLA map of the world: A comparison of HLA frequencies in 200 worldwide populations reveals diverse patterns for class I and class II

**DOI:** 10.3389/fgene.2023.866407

**Published:** 2023-03-23

**Authors:** Esteban Arrieta-Bolaños, Diana Iraíz Hernández-Zaragoza, Rodrigo Barquera

**Affiliations:** ^1^ Institute for Experimental Cellular Therapy, University Hospital Essen, Essen, Germany; ^2^ German Cancer Consortium (DKTK), partner site Essen/Düsseldorf, Heidelberg, Germany; ^3^ Molecular Genetics Laboratory, Escuela Nacional de Antropología e Historia (ENAH), Mexico, Mexico; ^4^ Department of Archaeogenetics, Max Planck Institute for Evolutionary Anthropology (MPI-EVA), Leipzig, Germany

**Keywords:** human leukocyte antigen, population differentiation, allele frequencies, MHC, anthropology, population genetics, genetic drift

## Abstract

HLA frequencies show widespread variation across human populations. Demographic factors as well as selection are thought to have shaped HLA variation across continents. In this study, a worldwide comparison of HLA class I and class II diversity was carried out. Multidimensional scaling techniques were applied to 50 *HLA-A* and *HLA-B* (class I) as well as 13 *HLA-DRB1* (class II) first-field frequencies in 200 populations from all continents. Our results confirm a strong effect of geography on the distribution of HLA class I allele groups, with principal coordinates analysis closely resembling geographical location of populations, especially those of Africa-Eurasia. Conversely, class II frequencies stratify populations along a continuum of differentiation less clearly correlated to actual geographic location. Double clustering analysis revealed finer intra-continental sub-clusters (e.g., Northern and Western Europe vs. South East Europe, North Africa and Southwest Asia; South and East Africa vs. West Africa), and HLA allele group patterns characteristic of these clusters. Ancient (Austronesian expansion) and more recent (Romani people in Europe) migrations, as well as extreme differentiation (Taiwan indigenous peoples, Native Americans), and interregional gene flow (Sámi, Egyptians) are also reflected by the results. *Barrier* analysis comparing D_ST_ and geographic location identified genetic discontinuities caused by natural barriers or human behavior explaining inter and intra-continental HLA borders for class I and class II. Overall, a progressive reduction in HLA diversity from African to Oceanian and Native American populations is noted. This analysis of HLA frequencies in a unique set of worldwide populations confirms previous findings on the remarkable similarity of class I frequencies to geography, but also shows a more complex development for class II, with implications for both human evolutionary studies and biomedical research.

## Introduction

Human populations share a common origin in Sub-Saharan Africa (Reyes-Centeno, 2016). However, a long history of migrations and admixture events (Hellenthal et al., 2014), as well as gene flow from other hominins (Abi-Rached et al., 2011; Wall and Yoshihara Caldeira Brandt, 2016), combined with geographic (Novembre et al., 2008) and linguistic (Baker et al., 2017) barriers have produced a complex pattern of population differentiation across the globe. This complex history is reflected and can be traced by our genetic makeup (Genomes Project et al., 2015). Indeed, analysis of polymorphism in the human genome has revealed the relationships between different human groups (Li et al., 2008; Auton et al., 2009; Genomes Project et al., 2015), and is a powerful tool to understand our populations’ genetic history. The Human Leukocyte Antigens (HLA) bear the most extensive polymorphism in the human genome. Currently, according to the IPD-IMGT/HLA database (release 3.50 October 2022) (Robinson et al., 2020), more than 35,000 alleles have been discovered for this genetic system, and it is estimated that the total variation in the whole human population could reach several million alleles (Robinson et al., 2017). Importantly, allelic and haplotypic frequencies of HLA genes vary dramatically across human populations (Fernandez Vina et al., 2012; Askar et al., 2013). Knowledge of HLA allele and haplotype frequencies is of special interest for medical fields such as cellular therapy and transplantation of organs and stem cells (Tiercy and Claas, 2013; Pidala et al., 2014), disease association studies and genetic epidemiology (Ghodke et al., 2005), forensics, and pharmacogenetics (Pirmohamed et al., 2015), but can also be used to assess genetic diversity in human populations (Sanchez-Mazas et al., 2011). Of note, due to its central role in immune responses, the HLA system may be influenced both by demographic as well as by selective mechanisms, in particular balancing selection (Meyer et al., 2006). It is hence of interest to explore the interplay between selective and demographic events on population differentiation and how this has shaped HLA genetic structure across the world. In particular, a comparative analysis of class I and class II variation could also provide insights into the differential population genetics dynamics of these two functionally different components of the HLA system. Various landmark studies have previously examined HLA variation at the continental (Di and Sanchez-Mazas, 2011; 2014; Sanchez-Mazas et al., 2013; Nunes et al., 2014; Sanchez-Mazas et al., 2017a; Sanchez-Mazas et al., 2017b) and global (Middleton et al., 2000; Williams et al., 2001; Mack et al., 2007; Solberg et al., 2008; Nunes et al., 2010; Buhler and Sanchez-Mazas, 2011; Sanchez-Mazas et al., 2012a; Riccio et al., 2013; Dos Santos Francisco et al., 2015; Buhler et al., 2016; Nunes et al., 2022) level. Inspired by these previous efforts, here we aimed at contributing to the study of global patterns of HLA variation by comparing two unique datasets of human population samples from all regions of the world using multidimensional scaling (MDS) and clustering approaches, as well as genetic barrier analysis based on first-field frequencies of 50 class I and 13 class II HLA allele groups.

## Materials and methods

### Population data

HLA frequency data were extracted from peer-reviewed journal articles, proceedings from the International Histocompatibility Workshops (IHWS), and/or the Allele Frequency Net Database (Gonzalez-Galarza et al., 2020; Gonzalez-Galarza et al., 2021) or the HLA-NET platform (Sanchez-Mazas et al., 2012b; Nunes et al., 2014). Population samples selected consisted of healthy individuals included in anthropology or disease association studies or bone marrow donor registries that had available first or second-field DNA-based HLA typing for either class I and/or class II. Inclusion of populations from all regions and major ethnicities of the world was sought. In addition to populations native to each of ten worldwide geographical regions (Solberg et al., 2008), migrant non-native populations were also included to investigate their relation to the extant populations from their ancestral homeland and those from the region where they migrated to. Mixed-ancestry (i.e., “Mestizo”) populations were not included on account of their complex multi-continental admixtures, which have been analyzed elsewhere (Arrieta-Bolanos et al., 2012; Barquera et al., 2020a; Arrieta-Bolanos et al., 2020). Two datasets were constructed: one consisting of class I frequencies (*HLA-A* and *HLA-B*) and another one based on *HLA-DRB1* frequencies. These loci were selected since they show the highest polymorphism of the HLA genes (Robinson et al., 2020), as well the strongest geographic (Di et al., 2017) and ethnic-specific variation (Solberg et al., 2008), while maximizing also the number of populations available for analysis (Gonzalez-Galarza et al., 2020). When available, high-resolution *HLA-A*, *-B*, and *-DRB1* data were reduced to first-field (i.e., allele groups; 50 for class I and 13 for *HLA-DRB1*) in order to homogenize the resolution for all datasets for MDS and clustering analyses. Class I and class II data were compiled and analyzed independently due to their different roles in the immune system and the potential for differential effects of immune mediated selective pressures across classes. Dataset quality control included assessment of cumulative frequencies for each locus for each population to ensure it corresponded to what was reported on allelefrequencies.net or the respective publication for the allele groups investigated, and individual cross-checking of entries for selected populations. Evidence of Hardy-Weinberg equilibrium (HWE) in the population sample was not considered as a pre-requisite for inclusion in the study, but information on this was retrieved from the original publications when available. In a subset of samples for which raw data was available, HWE was tested in this work as explained below. In total, both the class I and class II datasets included 200 and 197 population samples and consisted of 10,200 and 2,561 frequency entries, respectively. In total, the population array included 712,462 chromosomes for class I and 370,794 chromosomes for class II. Details on the populations included in this study, including their nomenclature, abbreviation codes, literature source, sample size, HWE status, geographical region, and corresponding references can be found in Supplementary Tables S1, 2.

### Multidimensional scaling and clustering analyses

The HLA class I and class II frequency data of the populations in each dataset were analyzed by clustering analysis and Principal Coordinates Analysis (PCoA), both based on Euclidean distances, using the Multi-Variate Statistical Package (MVSP version 3.22, Kovach Computing Services, Anglesey, Wales) as explained below. *HLA-A* and *HLA-B* frequencies were analyzed both separately and together, while *HLA-DRB1* frequencies were analyzed separately. A double clustering analysis was performed and dendrograms were generated for both the populations and the HLA allele groups. The clustering method was based on minimum variance of squared Euclidean distances with a randomized input order. The similarity matrices and their associated sorted data were used to construct heat maps showing the variation of HLA frequencies in the clustered data. PCoA analysis was carried out for each dataset and locus, and axes were extracted according to Kaiser’s rule (Kaiser, 1960). The distance matrix generated for each dataset was further analyzed to compare intra and inter-continental subgroup distance distributions.

For subsequent comparative analyses of the MDS output results, population samples were grouped according to their geographic location into continental and subcontinental regions. Migrant populations (i.e. those descending from populations located in a different geographical region) including African-descendant populations in the Americas and European-descendant populations in the United States, Australia, and South Africa were regrouped to their ancestral region for further statistical analysis of the PCoA output and allele group diversity comparisons.

### Detection of genetic discontinuity in worldwide HLA diversity

A set of populations (Supplementary Tables S3–6) for which both HLA class I (*HLA-A*, *-B*) and class II (HLA-DRB1) data was available was used for genetic discontinuity testing using the software *Barrier* v.2.2 (Manni et al., 2004). To construct the genetic distances matrix, we used the HLA frequency data available for the selected populations and calculated the corrected D_st_ genetic distance using the software POPTREEW (Takezaki et al., 2014) with a bootstrap of 1,000 replications. We used the reported coordinates for each population [available in the Allele Frequency Net Database (Gonzalez-Galarza et al., 2020) unless otherwise stated]. We added virtual points in the Pacific Ocean region and the Mediterranean Sea to appropriately model neighboring populations (i.e., preventing the software from assigning North African and Mediterranean populations or Beringian and Oceanian populations as neighboring clusters). To better depict the demographic and natural history of Native American, East Asian, and Oceanian populations, we shifted the longitude coordinates to include America as a continuum to Asian and Oceanian populations by adding 210° to the populations located to the west of the Greenwich meridian (0°) and subtracted 150° to the populations located to the east (Supplementary Tables S3–6), unless otherwise indicated (i.e., populations to the west of the Greenwich meridian part of Europe or Africa that otherwise would be translocated to the East). Then, we computed k = 30 barriers separately for each locus, for both class I loci (*HLA-A*, *HLA-B*), and for class I and *HLA-DRB1* in each of four geographical regions. Finally, to assess the statistical significance of the computed barriers, we analyzed the resampled bootstrapped (k = 100) matrices to test for HLA system-wide discontinuities.

### Statistical analyses

HWE testing was performed for a subset of samples with available raw data (Supplementary Tables S1, 2) using Arlequin ver. 3.5 (Excoffier and Lischer, 2010). *p*-values <0.016 (after Bonferroni correction) were considered statistically significant. Euclidean distances between populations calculated from the PCoA were compared using analysis of variance (ANOVA) and Tukey’s method with adjustment for multiple comparisons. Similarly, the number of HLA allele groups observed in each population was compared across continental groups with ANOVA and Tukey’s multiple comparisons test. For these analyses, a *p*-value of <0.05 was considered statistically significant. ANOVA and descriptive statistical analyses were performed with Prism (version 9.1.2, GraphPad Software Inc., La Jolla, California).

## Results

### Population sample metrics

The population samples for HLA class I analysis included 200 datasets with sample sizes ranging from 36 to 50,614 individuals. For *HLA-DRB1*, the dataset included 197 population samples with sample sizes ranging from 16 to 39,689 individuals. Class I datasets were published between 1995 and 2021 with 70 (35%) published in or after 2010. For *HLA-DRB1*, datasets were published between 1992 and 2020, with 76 (39%) published from 2010 onwards. These populations samples included datasets from 10 geographical regions (Mack et al., 2007), namely Sub-Saharan Africa (SSA), Europe (EUR), North Africa (NAF), Southwest Asia (i.e. Middle East and South Asia, SWA), northern and southern East Asia (NEA, SEA), northern (NAM, including Inuit from Greenland and populations from Central America) and southern America (SAM), Oceania (OCE), and Australia (AUS). A summary of the most relevant aspects of these datasets can be found in Table 1. Overall, median sample size was comparable across continental groups and class I and class II datasets.

**TABLE 1:** Summary of the population datasets included in the analyses and their main characteristics.

	*HLA-A*, *HLA-B*				*HLA-DRB1*			
Region	Number of datasets	Median sample size	Range	Total number of individuals	Number of datasets	Median sample size	Range	Total number of individuals
SSA	29	138	36–5,928	9,900	27	102	32–5,928	9,512
NAF	7	100	73–118	674	7	98	82–118	695
SWA	27	175	42–23,000	78,467	12	155.5	72–15,542	21,663
EUR	65	154	50–39,979	120,899	46	186.5	43–39,689	104,914
NEA	11	158	50–18,604	20,209	16	185.5	50–4,128	11,442
SEA	11	236	50–50,614	96,277	30	104	25–16,807	29,155
NAM	23	100	42–8,525	14,963	23	60	16–3,000	5,316
SAM	14	81.5	40–168	1,165	20	56.5	16–144	1,273
AUS	4	147	75–891	1,260	3	103	41–191	335
OCE	9	111	49–11,499	12,417	13	65	21–292	1,095
**Total**	**200**	**131**	**36–50,614**	**356,231**	**197**	**103**	**16–39,689**	**185,738**

Bold values indicate the totals for each column.

### Clustering analysis reveals HLA worldwide population relationships

**FIGURE 1:**
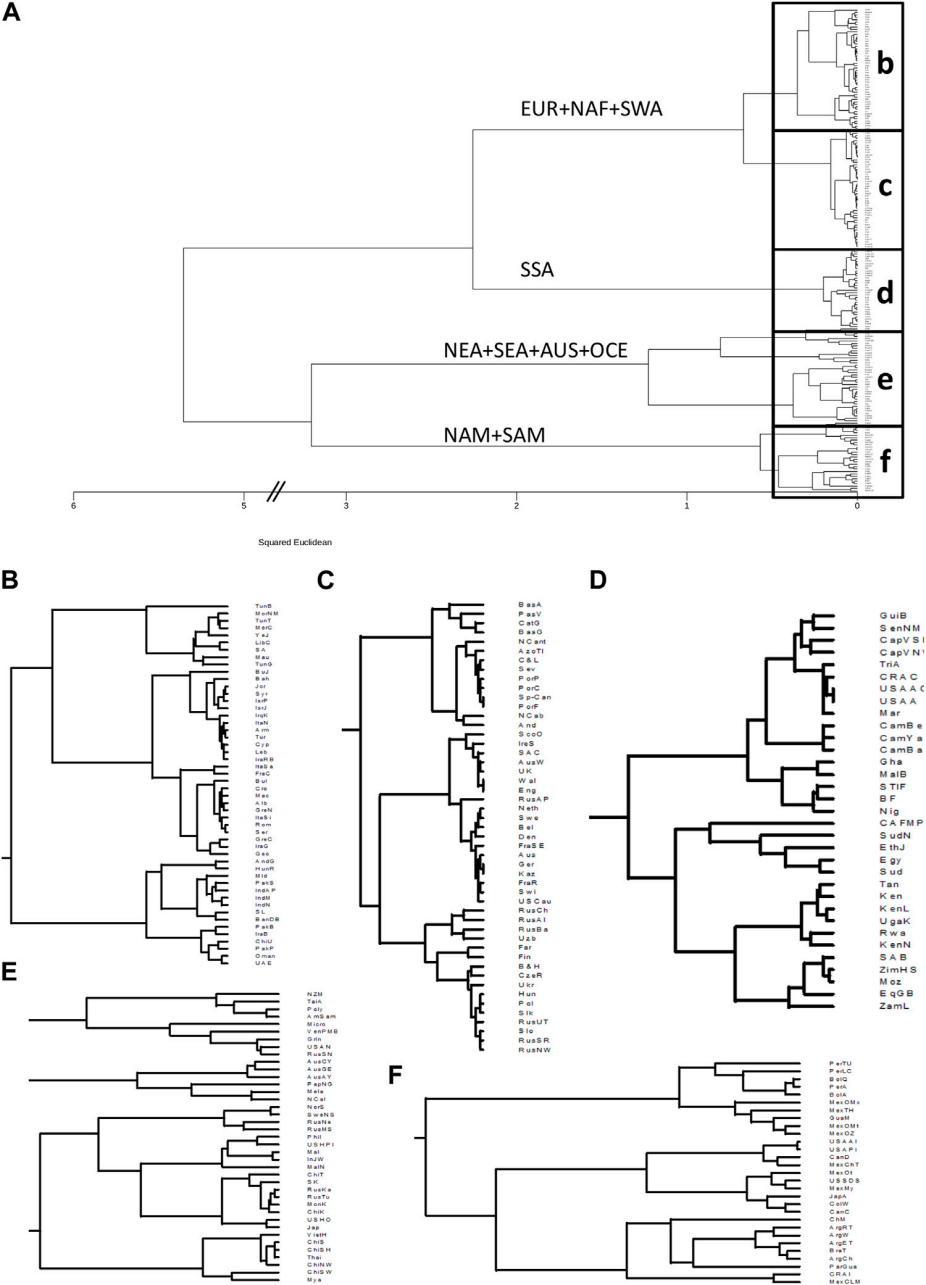
Clustering based on HLA class I frequencies identifies continental and intracontinental population similarities. **(A)**: Dendrogram generated with minimum variance clustering analysis of squared Euclidean distances calculated using 50 HLA-A and HLA-B allele group frequencies in 200 worldwide populations. Main branches are labelled according to the predominant continental origin of populations forming its clusters. b-f. Panels show the detail of the boxes in a. **(B)**: NAF, North Africa; SWA, Southwest Asia. **(C)**: EUR, Europe. **(D)**: SSA, Sub-Saharan Africa. **(E)**: NEA, Northeast Asia; SEA, Southeast Asia; AUS, Australia; OCE, Oceania. **(F)**: NAM, North America; SAM, South America.

**FIGURE 2:**
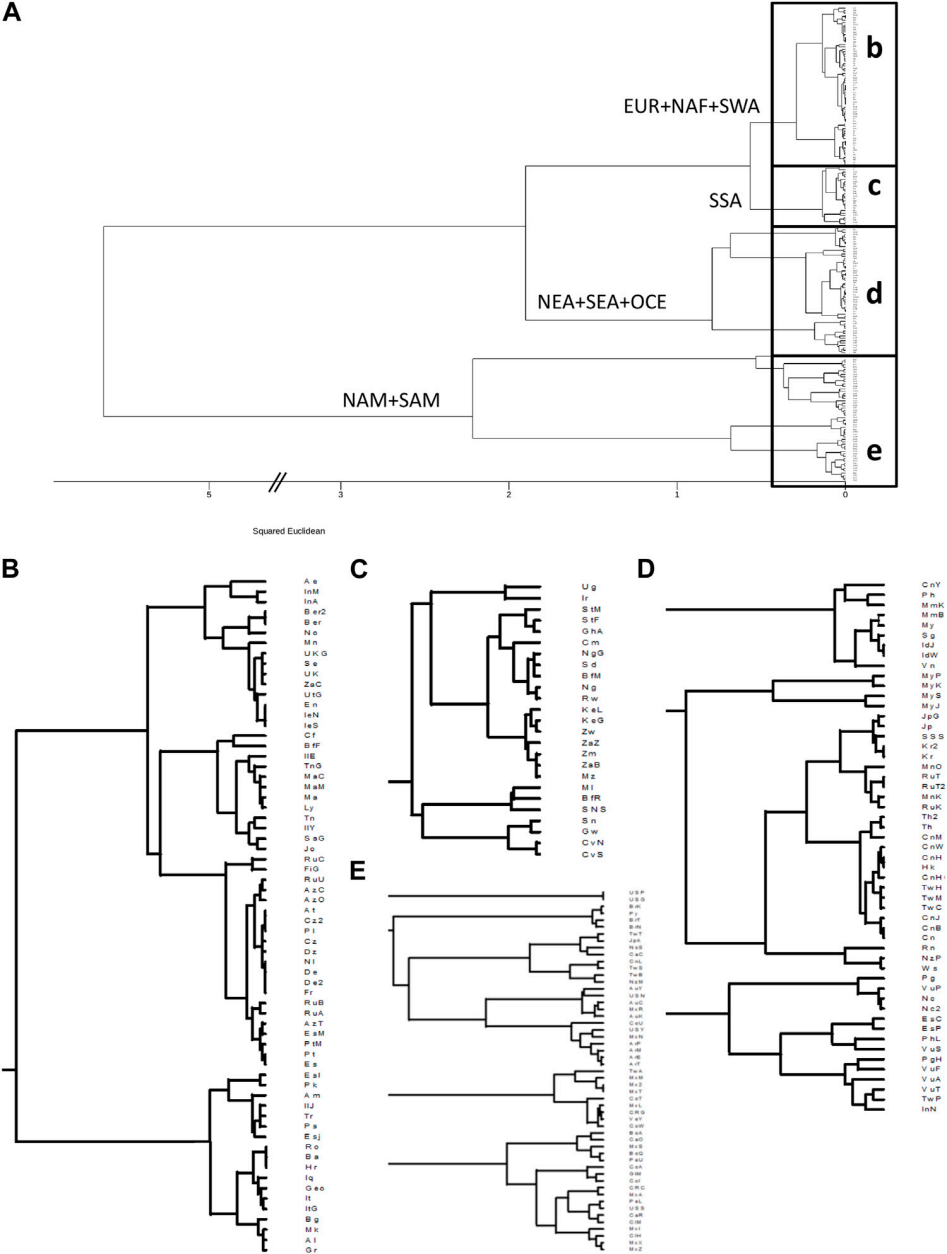
Clustering based on HLA-DRB1 frequencies identifies continental and intracontinental population similarities for HLA class II. **(A)**: Dendrogram generated with minimum variance clustering analysis of squared Euclidean distances calculated using 13 HLA-DRB1 allele group frequencies in 197 worldwide populations. Main branches are labelled according to the predominant continental origin of populations forming its clusters. b-e. Panels show the detail of the boxes in a. **(B)**: NAF, North Africa; SWA, Southwest Asia; EUR, Europe. **(C)**: SSA, Sub-Saharan Africa. **(D)**: NEA, Northeast Asia; SEA, Southeast Asia; OCE, Oceania. **(E)**: NAM, North America; SAM, South America.

The results of the clustering analysis of the populations for class I (*HLA-A* and *HLA-B* together) and class II are shown in Figure 1 and Figure 2, respectively. For class I (Figure 1A), the biggest split in the tree separates the Native American, East Asian, AUS, and OCE populations from those of SSA, EUR, NAF, and SWA. Subsequent splits separate the Native Americans from the oriental populations, and the SSA from the cluster formed by EUR, NAF, and SWA.

Closer examination of the class I phylogenetic tree (Figures 1B–F), reveals that the EUR + NAF + SWA cluster separates into two groups defined mainly by the Alps and Carpathians (Figures 1B,C), with populations south and east of these mountain ranges (Figure 1B) clustering into four subgroups, namely 1) NAF and Arabians, 2) populations from the Near East, the Levant 3) Southeast Europeans and populations from the Mediterranean islands, and 4) South Asians. Interestingly, Romani populations from Hungary (Inotai et al., 2015) and Spain (Gonzalez-Galarza et al., 2020) cluster with the populations from South Asia, and the sample of Bukharan Jews (Manor et al., 2016) clusters with the populations of the Levant.

Sub-clusters to the north and west of the European mountain ranges (Figure 1C) include 1) Iberians and populations from the Balearic, Azores (Gonzalez-Galarza et al., 2020), and Canary (Romon et al., 2016) islands, and 2) the populations north of the Pyrenees, split into the British Isles and the continental Germanic (western) and Slavic-Uralic (eastern) linguistic areas. Similar results for Europe have been previously observed using higher resolution data (Nunes et al., 2010; Riccio et al., 2013). European-descendant populations from the United States (Leffell et al., 2007), South Africa (Paximadis et al., 2012), and Australia (Gonzalez-Galarza et al., 2020) also cluster together with the North-western Europeans.

The SSA cluster (Figure 1D), is split into West Africans and populations from the southern and eastern parts of the continent. Previous research has demonstrated differences between these subregions in Africa (Nunes et al., 2010). Interestingly, despite well-documented gene flow from non-African populations, African-descendant populations in the United States (Leffell et al., 2007; Maiers et al., 2007), Costa Rica (Arrieta-Bolanos et al., 2019), Martinique (Bera et al., 2001), and Trinidad and Tobago (Gonzalez-Galarza et al., 2020) cluster together among West Africans. Of note, the sample of Egyptians (Nunes et al., 2014) is found among the SSA populations, probably reflecting post-Roman SSA gene flow (Schuenemann et al., 2017).

The picture becomes more complicated for populations from East Asia, OCE and AUS (Figure 1E), and the Americas (Figure 1F). However, some patterns emerge as New Zealand Maori (Edinur et al., 2012), Polynesians (Edinur et al., 2012), and American Samoans (Mack et al., 2007) cluster with Taiwan indigenous peoples (Gonzalez-Galarza et al., 2020), whereas the populations from the extreme north-eastern part of Asia (i.e. the Russian Nivkhis (Lou et al., 1998)) cluster with the samples from Alaskan natives (Leffell et al., 2002) and the Inuit from Greenland (Grunnet et al., 1996) (Figure 1F). Moreover, Papuans (Main et al., 2001) and New Caledonians (Main et al., 2001) cluster with the Indigenous Australians, whereas the rest of the Asian populations split into three groups formed by 1) continental and 2) insular SEA, and 3) NEA. Of note, the Russian Kalmyks (Loginova et al., 2021), although settled in Europe, cluster with other populations of Mongolian ethnic descent, and Arctic populations west of the Urals (Evseeva et al., 2002; Johansson et al., 2008; Harbo et al., 2010) are also found in the Asian cluster.

In the Americas (Figure 1F), indigenous populations form a distinct group with local (i.e. Andean, Mesoamerican, Lowlander) patterns of clustering, albeit with complex relations as previously reported (Nunes et al., 2010). Of note, the Ainu from Japan cluster with the Native Americans, something already previously observed (Bannai et al., 1996; Bannai et al., 2000).

When the clustering was performed separately with *HLA-A* or *HLA-B* frequencies, some differences were observed. For *HLA-A* (Supplementary Figure S1), the first split separated AUS and OCE populations from the rest, while the second separated NAM and SAM from SSA, NAF, SWA, EUR, NEA, and SEA. The SSA separate next, leaving the split between the East Asian populations and the EUR + NAF + SWA as the fourth one, with some South Asian populations remaining in a cluster next to the SEA. On the other hand, when only *HLA-B* frequencies were used (Supplementary Figure S2), the main splits resembled more closely those observed in the combined analysis, albeit with some reshuffling of the regional subclusters.

For *HLA-DRB1*, the clustering results roughly resemble the major patterns observed for class I, albeit with some exceptions. In this case, the samples from Native Americans appear as a first breakout group (Figures 2A,B), and AUS populations cluster among them. Of note, the remote Rapa Nui from Easter Island (Thorsby, 2012) cluster with Polynesian (Edinur et al., 2012) and Samoan (Mack et al., 2007) populations (Figure 2C).

### Double clustering identifies HLA allele group variation patterns across populations

**FIGURE 3:**
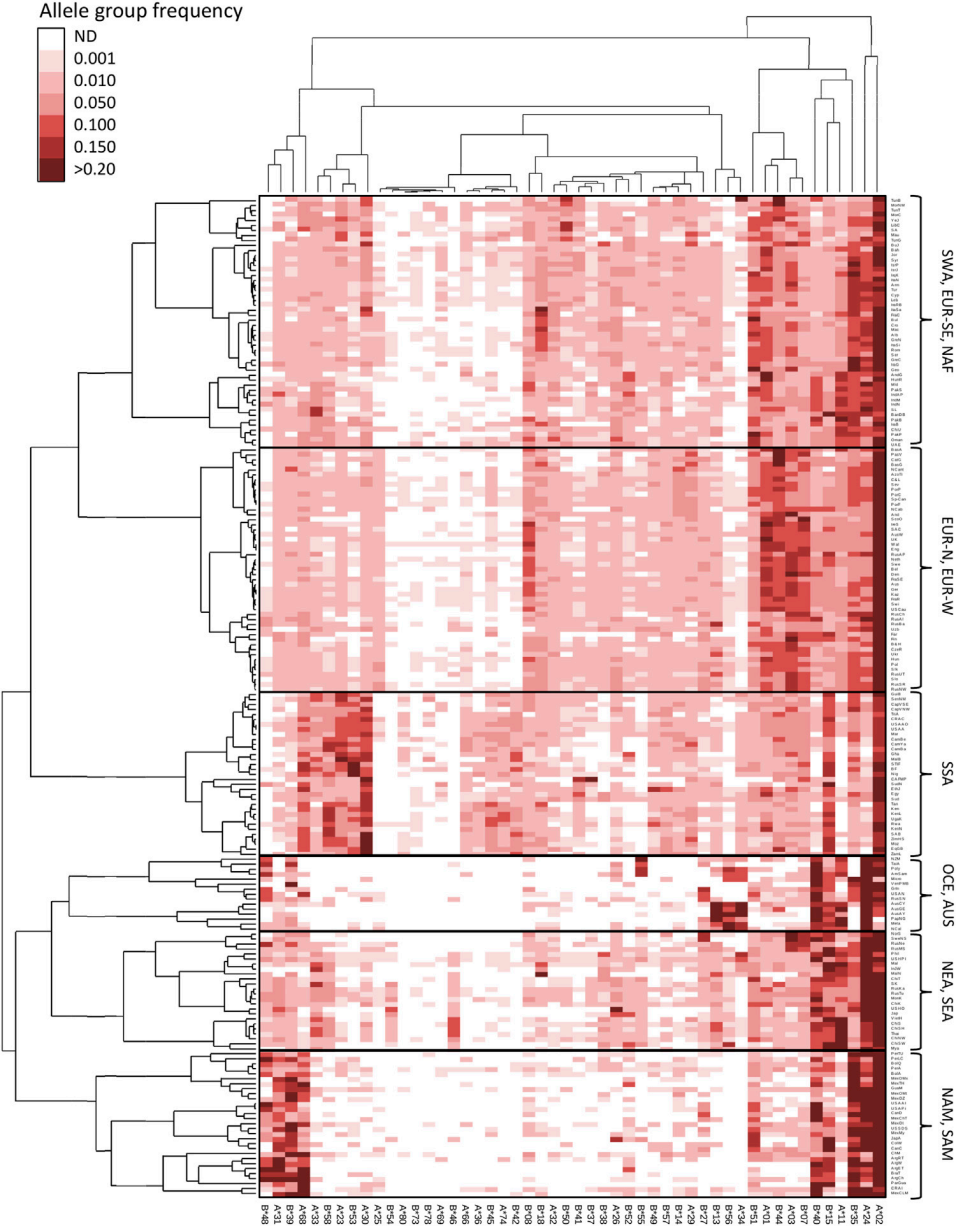
Double clustering reveals worldwide patterns of HLA class I allele group frequencies. Unsupervised double clustering-based heat map of 50 *HLA-A* and *HLA-B* allele groups in 200 worldwide populations. Frequencies of individual allele groups are plotted according to the allele group (top and bottom) and population (left and right) minimum variance squared Euclidean distance dedrograms. Continental grouping of populations is indicated on the right side. AUS, Australia; EUR-N, Northern Europe; EUR-W, Western Europe; EUR-SE, South-eastern Europe; NAF, North Africa; NAM, North America; NEA, Northeast Asia; OCE, Oceania; SAM, South America; SEA, Southeast Asia; SWA, Southwest Asia; SSA, Sub-Saharan Africa. ND, not detected.

**FIGURE 4:**
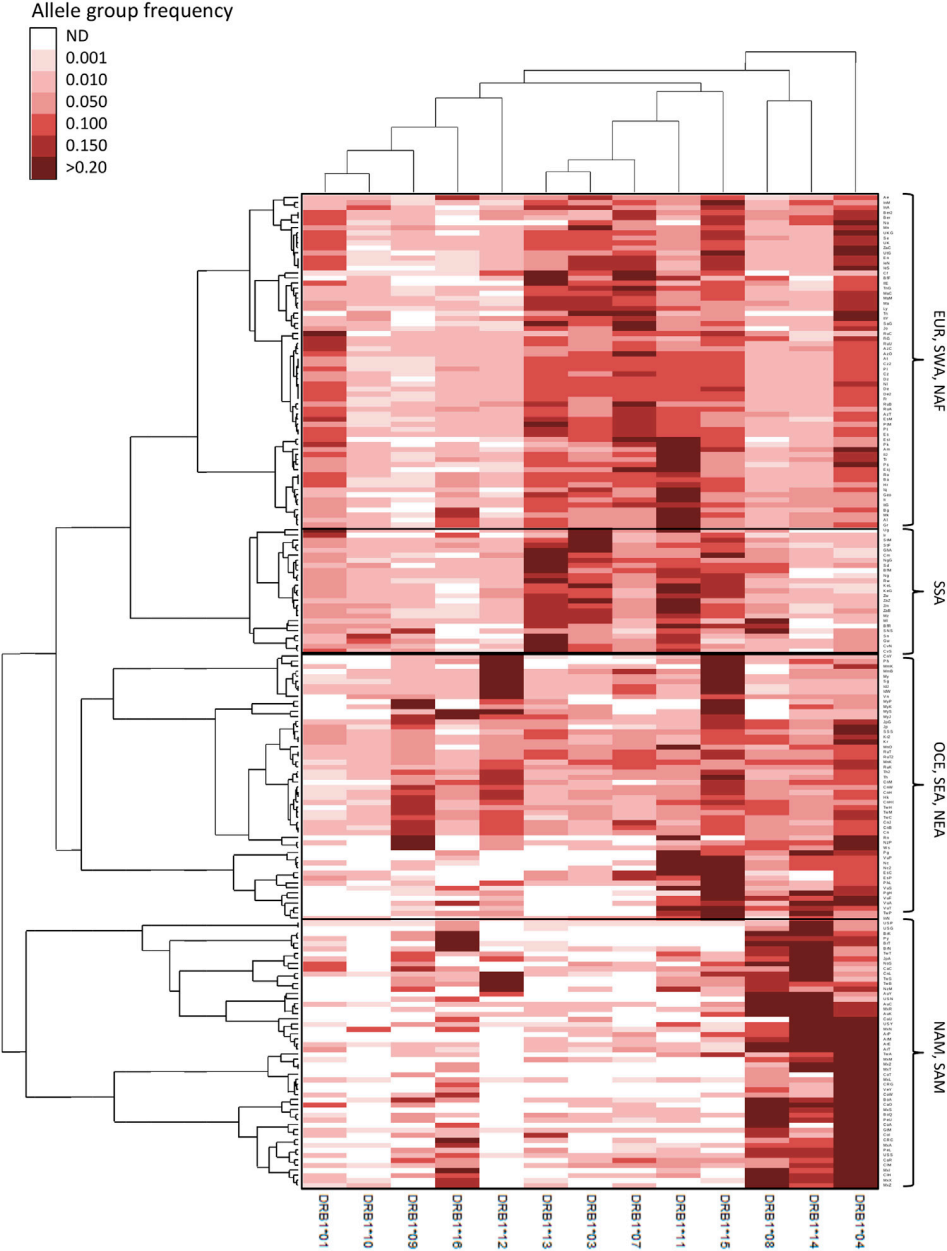
Double clustering reveals worldwide patterns of HLA class II allele group frequencies. Unsupervised double clustering-based heat map of 13 *HLA-DRB1* allele groups in 197 worldwide populations. Frequencies of individual allele groups are plotted according to the allele group (top and bottom) and population (left and right) minimum variance squared Euclidean distance dedrograms. Continental grouping of populations is indicated on the right side. AUS, Australia; EUR, Europe; NAF, North Africa; NAM, North America; NEA, Northeast Asia; OCE, Oceania; SAM, South America; SEA, Southeast Asia; SWA, Southwest Asia; SSA, Sub-Saharan Africa. ND, not detected.

In order to illustrate worldwide HLA allele group variation patterns, we performed a double clustering procedure and integrated the results of the population and the HLA allele group clustering in an unsupervised hierarchical HLA frequency heat maps as shown in Figure 3 and Figure 4 for class I and class II, respectively. For HLA class I (Figure 3), apart from HLA-A*02 having a high frequency in almost all human populations and together with A*24 concentrating most of the *HLA-A* diversity in NAM, SAM, OCE, NEA, and SEA populations, additional patterns reveal HLA signatures in specific continental groups, with high frequencies for B*15, A*30, and A*68 characterizing SSA populations and A*23, A*33, B*53, B*58 being particularly frequent in West Africans. Northern and western EUR populations share high frequencies for A*01, A*03, B*07, and B*44, which set them apart from south-eastern EUR, NAF, and SWA populations, in turn characterized by higher B*51 frequencies. NAM and SAM populations show very high frequencies of A*31, A*68, B*35, B*39, and B*48, with B*40 and B*15 driving the split between populations in these groups. Indigenous Australians, Melanesians and Papuans (Main et al., 2001) share a distinctive signature formed by A*34, B*13, and B*56 also present at lower frequencies in Polynesians (Edinur et al., 2012), Micronesians (Gao et al., 1997), and the Maori (Edinur et al., 2012). The latter three groups differentiate on account of their high B*55 frequencies, while populations from North Eastern Siberia (Lou et al., 1998), Alaska (Leffell et al., 2002), and Greenland (Grunnet et al., 1996) share very high B*27 frequencies, something only found elsewhere in Arctic Europe. Finally, NEA and SEA populations share very high frequencies of A*11, B*15, and B*40, as well as the highest B*46 and B*56 frequencies worldwide. Of note, NEA and SEA differ starkly in their A*34 frequencies, which in the latter could represent a signature of admixture with the pre-East Asian migration substrate of the region (McColl et al., 2018).

For *HLA-DRB1* (Figure 4), distinctive patterns include very high frequencies for DRB1*04, *08, *14, and, in some cases, *16 in NAM and SAM; high DRB1*15 frequencies in OCE and insular SEA, the latter also characterized by high DRB1*12 frequencies; and DRB1*09 being highly prevalent in both East Asian and OCE populations. On the other hand, allele groups DRB1*01, *03, *04, *07, and *15 have all high frequencies in EUR, SWA and NAF populations, with very high frequencies for DRB1*11 characterizing most SE Europeans as previously observed (Nunes et al., 2014). Finally, very high DRB1*11, *13, and *15 in addition to low DRB1*04 frequencies characterize SSA populations.

**FIGURE 5:**
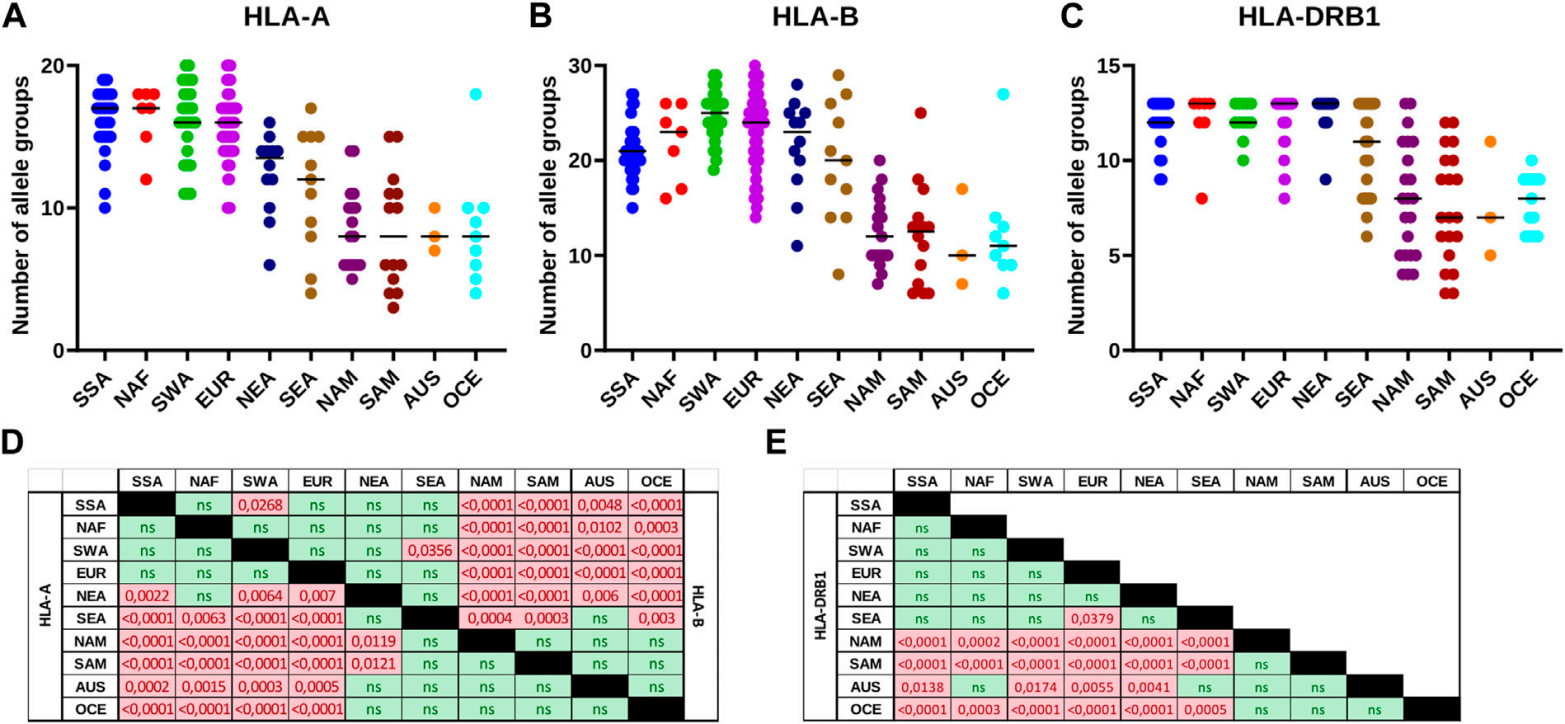
The number of detectable HLA class I and class II allele groups differs across continental groups. **(A-C)**: The number of HLA-A, HLA-B or HLA-DRB1 allele groups detected in each of the populations included in this study is plotted per continental group. Horizontal bar represents the median number of allele groups detected. **(D,E)**: Matrices of statistical testing results for the differences in number of HLA allele groups detected per continental population group. **(D)**: Class I matrix. Lower left half shows results for HLA-A, and upper right half those for HLA-B. **(E)**: HLA-DRB1 matrix. p-values for statistically significant results in pairwise comparisons are coloured in red. AUS, Australia; EUR, Europe; NAF, North Africa; NAM, North America; NEA, Northeast Asia; OCE, Oceania; SAM, South America; SEA, Southeast Asia; SWA, Southwest Asia; SSA, Sub-Saharan Africa. ns, not significant.

Analysis of HLA allele group frequencies patterns revealed a progressive reduction of the diversity proportional to the distance from the African continent. This is especially evident for HLA class I, where a more balanced allele group frequency pattern in SSA, NAF, SWA, and EUR (median of 16–17 and 21–25 allele groups detected for *HLA-A* and *HLA-B*, respectively) contrasts with much more polarized frequencies in Native Americans, OCE, and AUS (medians of 8 and 10–12.5) (Figures 5A,B,D). East Asian populations have only slightly reduced numbers of detected allele groups (medians 14–15 and 20–23). A similar significant diversity reduction is observed for HLA-DRB1 with a median of 12–13 allele groups observed in SSA, NAF, SWA, EUR, and NEA vs. only seven to eight in NAM, SAM, OCE, and AUS (Figures 5C,E). The SEA populations, previously observed to have lower HLA diversity than their NEA counterparts (Di and Sanchez-Mazas, 2011; 2014), do manifest a trend to lower numbers of detected DRB1 allele groups, albeit without reaching statistical significance.

### PCoA confirms similarity between geography and HLA

**FIGURE 6:**
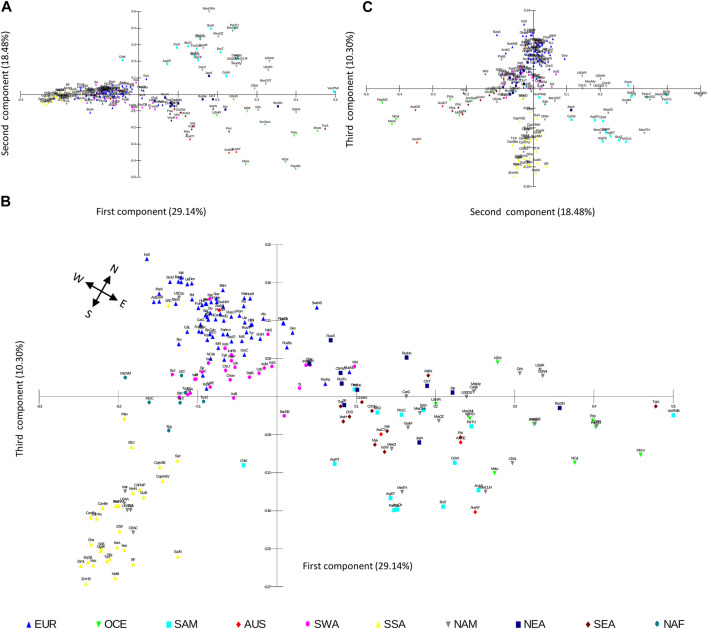
PCoA scatterplots based on HLA class I frequencies correlate with population geographic location. **(A–C)**. Population scatterplots for the PCoA case scores based on Euclidean distances calculated on 50 *HLA-A* and *HLA-B* allele group frequencies in 200 worldwide populations are plotted for the first (29.14% of variation), second (18.48%), and third (10.30%), components. A cardinal rose is included to guide pseudo-cardinal directions in b. Population samples are depicted according to their geographic continental classification. AUS, Australia; EUR, Europe; NAF, North Africa; NAM, North America; NEA, Northeast Asia; OCE, Oceania; SAM, South America; SEA, Southeast Asia; SWA, Southwest Asia; SSA, Sub-Saharan Africa.

To further explore the distribution of worldwide HLA diversity we performed PCoA analysis on the class I and HLA-DRB1 allele group frequencies of the population datasets. For class I, the first component (29.14% of the variation) separates Native American, AUS, OCE, NEA, and SEA populations from SSA, NAF, SWA, and EUR populations, and the second component (18.48%) further distinguishes NAM and SAM from East Asian and OCE and AUS populations (Figure 6A). A further third component (10.30%) shows the separation of EUR, SWA, NAF, and SSA populations, while also allowing the observation of subgroups within Native American populations and a progressive scattering of NEA, SEA, OCE, and AUS populations (Figures 1B,C). Interestingly, when the first and third components of this analysis are plotted, a remarkable similarity between the geographic location of the different population samples and the PCoA scatter plot is observed (Figure 6B). This coincidence with latitudinal and longitudinal location is particularly clear for the African continent (NAF, SSA), and Western (EUR, SWA) and Eastern (NEA, SEA) Eurasia as previously reported (Di and Sanchez-Mazas, 2011; 2014; Sanchez-Mazas et al., 2013; Sanchez-Mazas et al., 2017a).

When the PCoA is performed separately for *HLA-A* and *HLA-B*, the plots using the first three components for each gene resemble the general patterns described above, albeit with less clarity in the separation of the continental groups (Supplementary Figures S3, 4).

In turn, the PCoA based on HLA-DRB1 allele group frequencies produces somewhat different results. In this case, the first component (37.58%) also separates NAM, SAM, OCE, and AUS populations from SSA, NAF, SWA, EUR, NEA, and SEA, although some populations in the latter region also differentiate (Supplementary Figure S5A). The second (18.74%) and third (11.99%) components allow for the separation of SEA populations and a discrete differentiation between SSA and EUR, NAF, and SWA (Supplementary Figures S5B, C), with a more continuous scattering and less clear correlation with actual geographical location.

**FIGURE 7:**
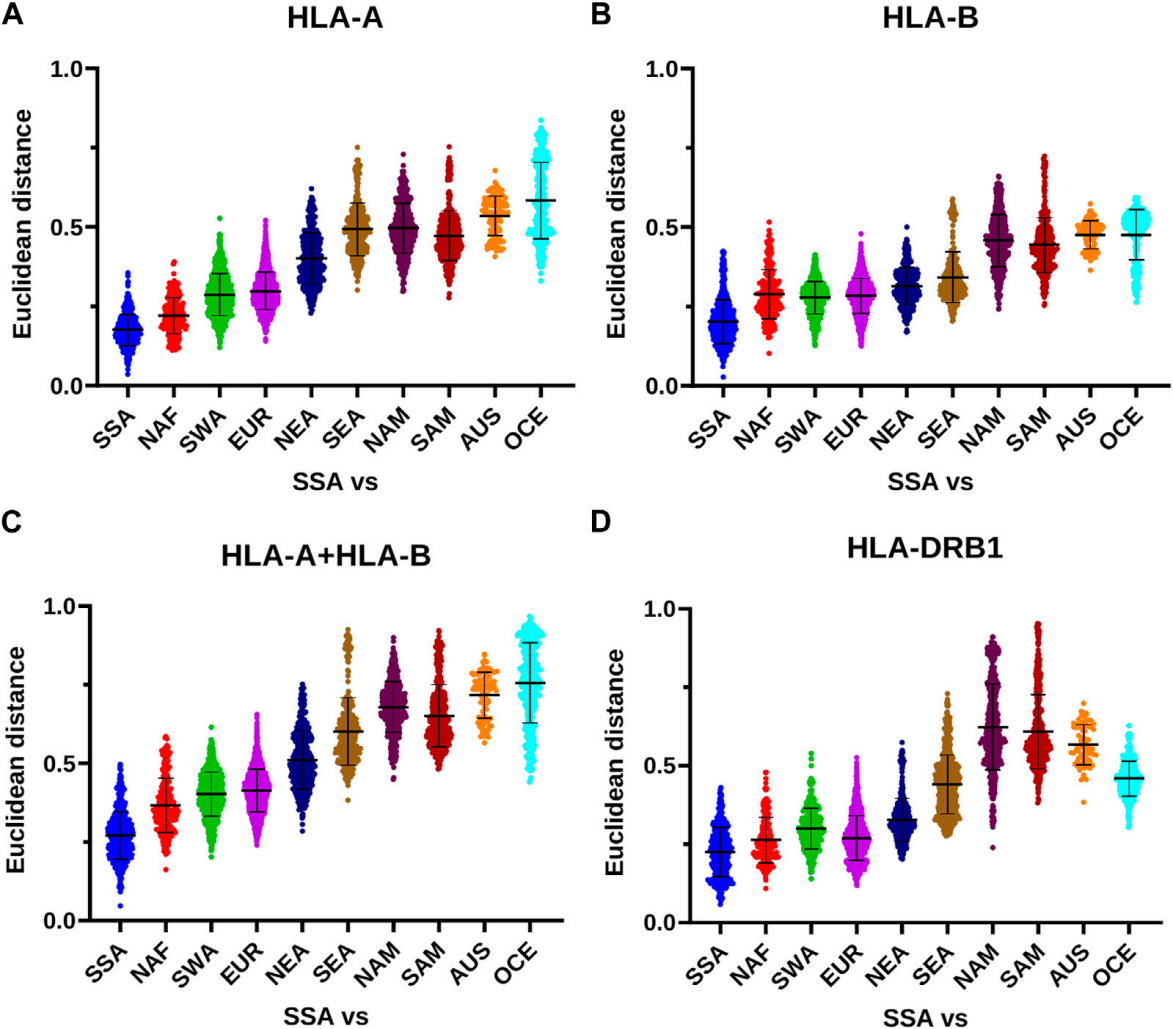
HLA class I and class II distances between SSA and other continental subgroups. **(A–D)** The PCoA Euclidean distances calculated on the frequencies of *HLA-A*, *HLA-B* (both independently and together), and *HLA-DRB1* allele groups among SSA populations (including SSA-descendant populations in the Americas) and between these and other non-SSA populations are plotted according to continental subgroups. Horizontal bar represents the mean distance with its standard deviation. AUS, Australia; EUR, Europe; NAF, North Africa; NAM, North America; NEA, Northeast Asia; OCE, Oceania; SAM, South America; SEA, Southeast Asia; SWA, Southwest Asia; SSA, Sub-Saharan Africa.

The differences in the effects of geographic distance on HLA distribution between class I and class II are also reflected by the continental analysis of the Euclidean distances generated by the PCoA. While for class I, and in particular for *HLA-A*, the distances between non-SSA and SSA populations tend to increase at a constant rate proportional to geographic distance from SSA and reach a maximum for AUS and OCE (Figures 7A–C; Tables 2–4), for *HLA-DRB1* they remain at lower levels for NAF, SWA, EUR, and NEA, and thereafter increase reaching a maximum for NAM and SAM (Figure 7D; Table 5). Overall, these results suggest a different dynamics of HLA diversity differentiation for class I vs. class II at this level of resolution, with class I reflecting better geographical location and distance between populations.

**TABLE 2:** Euclidean distances between continental groups calculated from *HLA-A* frequencies.

SSA vs.	SSA	NAF	SWA	EUR	NEA	SEA	NAM	SAM	AUS	OCE
Mean	0.176	0.221	0.287	0.299	0.400	0.492	0.495	0.473	0.535	0.583
SD	0.049	0.057	0.066	0.059	0.081	0.083	0.079	0.078	0.063	0.121
Minimum	0.036	0.112	0.121	0.141	0.229	0.301	0.297	0.277	0.407	0.330
Maximum	0.356	0.390	0.527	0.521	0.621	0.751	0.729	0.753	0.678	0.837

All intercontinental comparisons except SSAvsSEA, vs. SSAvsNAM, were statistically significant (*p* < 0.01) in the ANOVA. AUS, australia; EUR, europe; NAF, north africa; NAM, north america; NEA, northeast asia; OCE, oceania; SAM, south america; SEA, southeast asia; SWA, southwest asia; SSA, Sub-Saharan Africa; SD, standard deviation.

**TABLE 3:** Euclidean distances between continental groups calculated from *HLA-B* frequencies.

SSA vs.	SSA	NAF	SWA	EUR	NEA	SEA	NAM	SAM	AUS	OCE
Mean	0.203	0.289	0.278	0.283	0.315	0.342	0.458	0.444	0.476	0.477
SD	0.070	0.077	0.051	0.056	0.058	0.080	0.082	0.086	0.044	0.080
Minimum	0.028	0.102	0.128	0.125	0.170	0.204	0.242	0.254	0.365	0.265
Maximum	0.424	0.516	0.413	0.479	0.500	0.589	0.661	0.724	0.574	0.593

All intercontinental comparisons except SSAvsNAF, vs. SSAvsSWA, SSAvsNAF, vs. SSAvsEUR, SSAvsSWA, vs. SSAvsEUR, SSAvsAUS, vs. SSAvsOCE, and SSAvsAUS, vs. SSAvsNAM, were statistically significant (*p* < 0.05) in the ANOVA. AUS, australia; EUR, europe; NAF, north africa; NAM, north america; NEA, northeast asia; OCE, oceania; SAM, south america; SEA, southeast asia; SWA, southwest asia; SSA, Sub-Saharan Africa; SD, standard deviation.

**TABLE 4:** Euclidean distances between continental groups calculated from *HLA-A* and *HLA-B* frequencies.

SSA vs.	SSA	NAF	SWA	EUR	NEA	SEA	NAM	SAM	AUS	OCE
Mean	0.271	0.366	0.402	0.414	0.510	0.601	0.680	0.652	0.717	0.756
SD	0.076	0.086	0.070	0.067	0.093	0.108	0.081	0.098	0.073	0.128
Minimum	0.047	0.162	0.203	0.240	0.285	0.383	0.449	0.482	0.565	0.442
Maximum	0.496	0.585	0.615	0.655	0.752	0.925	0.900	0.922	0.847	0.967

All intercontinental comparisons were statistically significant (*p* < 0.01) in the ANOVA. AUS, australia; EUR, europe; NAF, north africa; NAM, north america; NEA, northeast asia; OCE, oceania; SAM, south america; SEA, southeast asia; SWA, southwest asia; SSA, Sub-Saharan Africa; SD, standard deviation.

**TABLE 5:** Euclidean distances between continental groups calculated from *HLA-DRB1* frequencies.

SSA vs.	SSA	NAF	SWA	EUR	NEA	SEA	NAM	SAM	AUS	OCE
Mean	0.226	0.263	0.300	0.270	0.328	0.441	0.624	0.608	0.567	0.459
SD	0.079	0.073	0.065	0.071	0.069	0.093	0.137	0.119	0.064	0.055
Minimum	0.058	0.109	0.140	0.119	0.203	0.276	0.239	0.382	0.384	0.304
Maximum	0.431	0.479	0.539	0.526	0.574	0.730	0.910	0.953	0.699	0.628

All intercontinental comparisons except SSAvsNAF, vs. SSAvsEUR, SSAvsSEA, vs. SSAvsOCE, and SSAvsNAM, vs. SSAvsSAM, were statistically significant (*p* < 0.01) in the ANOVA. AUS, australia; EUR, europe; NAF, north africa; NAM, north america; NEA, northeast asia; OCE, oceania; SAM, south america; SEA, southeast asia; SWA, southwest asia; SSA, Sub-Saharan Africa; SD, standard deviation.

### *Barrier* analyses highlights continental HLA class I and class II genetic discontinuities

**FIGURE 8:**
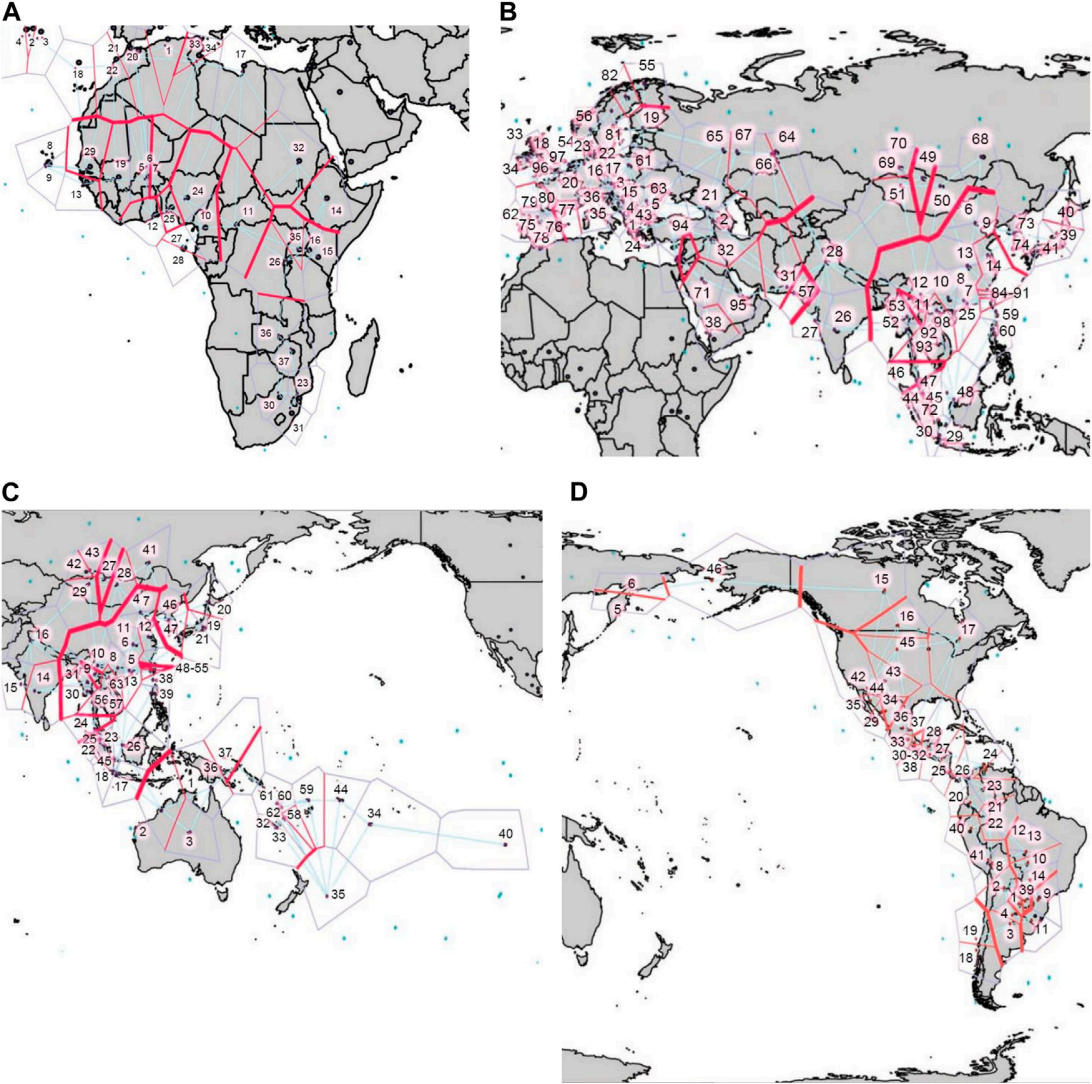
*Barrier* analysis for *HLA-A*, *-B*, and *-DRB1*. Plots show the statistically significant genetic barriers (thick red) determined with the joint analysis of *HLA-A*, *-B*, and *-DRB1* first-field frequencies for populations located in **(A)**. Africa, **(B)**. Eurasia, **(C)**. East Asia and the Pacific, and **(D)**. the Americas. Output of *Barrier* software was aligned to maps of each region to facilitate correlation with geographical context. Numbers correspond to the populations included in the *Barrier* analyses (Supplementary Tables S3-6).

Using *Barrier* analysis, we examined the correlation between HLA genetic discontinuity and geographic location of human populations in four continental regions, namely Africa, Eurasia, East Asia-Pacific, and the Americas. Although many overlapping genetic discontinuities were observed when each of the loci were analyzed independently (Supplementary Figures S6–8), the joint analysis of *HLA-A* and *HLA-B* (Supplementary Figure S9) and of *HLA-A*, *HLA-B*, and *HLA-DRB1* (Figure 8) was required to produce statistically significant barriers. For HLA class I, significant barriers were detected in Africa along the North Africa/Sub-Saharan/Mediterranean axis (Supplementary Figure S9A). Interestingly, this barrier runs south of some East African populations, likely reflecting gene flow from West Asia (Aamer et al., 2021). In Eurasia (Supplementary Figure S9B), in addition to some smaller barriers separating SWA from EUR and Central Asia, a major barrier was found between SEA and populations to the north and west, which was also detected in the East Asia-Pacific plot (Supplementary Figure S9C), in addition to another one separating AUS and OCE from SEA. Interestingly, the latter corresponds with the Wallace line that biogeographically divides Asia and Oceania (White et al., 2021), which could reflect ecological differences in both regions posing also different immune challenges to the inhabitants on both sides of the line. In turn, in the Americas (Supplementary Figure S9D) strong barriers are restricted to the extreme north, between Alaska Natives and Native American populations to the south, and between some SAM regions.

**FIGURE 9:**
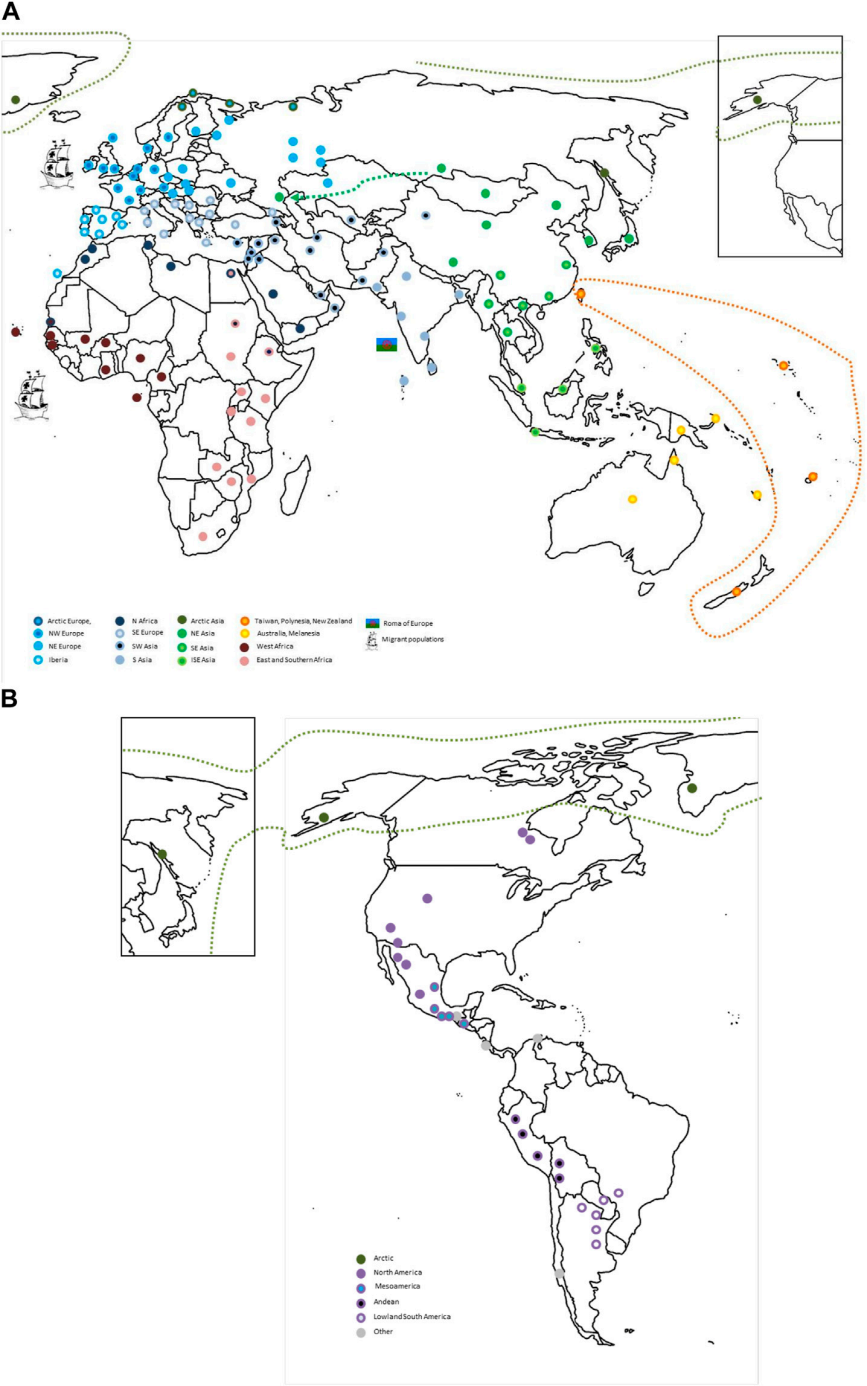
An HLA map of the world. The global human population relationships according to their HLA profiles as inferred from the integration of the analyses presented in this paper are depicted for **(A)** the Old World and **(B)** the Americas. The approximate locations for population samples included in the analyses presented in this paper are indicated with circles color-coded according to their sub-regional HLA similarities (e.g. West Africa vs. East and South Africa) as indicated in the legend. Migrant populations like the European Roma or European-descendant and African-descendant populations in other continents are represented with specific pictograms next to the ancestral region to which they show relatedness. In addition, the closeness of the Russian Kalmyks (Loginova et al., 2021) in the European Republic of Kalmykia to other NEA populations, that of the Taiwanese indigenous people and populations from Polynesia and New Zealand, and of Alaska Natives and Inuit with North-East Siberian populations are highlighted by a discontinuous arrow/line. For visual clarity, one circle can represent several population samples from the same region around that location, especially in regions with high sampling density (i.e. Europe). ISE, insular south-east; N, north; NE, north-east; NW, north-west; SE, south-east.

When both class I and *HLA-DRB1* frequencies are studied together (Figures 9A–D), statistically significant barriers mostly resemble the ones observed using class I only. However, some additional barriers become apparent including one arising between populations in Arctic Europe and those to the south (Figure 9B), and one between AUS and Near Polynesia and the rest of OCE (Figure 9C). In the Americas, the strongest barriers found are still in the extreme north, and between SAM populations separating those further south from Andean ones to the north, and those from populations in the South American lowlands to the east (Figure 9D).

## Discussion

In this study, we performed an analysis of global patterns of HLA diversity by analyzing two unique datasets of HLA class I and class II allele group frequencies in more than 200 population samples from around the world. Our clustering and multidimensional scaling analyses show how geographic closeness correlates with similar patterns of HLA variation, especially for class I. In addition, we show that PCoA results for class I resemble geographic locations in Afro-Eurasia, while class II patterns follow a more continuous pattern, less reminiscent of actual geographic location. Furthermore, we confirm a progressive restriction of HLA diversity according to distance from the African continent, with regions with extreme population differentiation such as AUS, OCE, NAM, and SAM showing the most restricted allele group repertoires. With *Barrier* analysis, we could also detect genetic discontinuities between well-defined ecological regions, a reminiscence of both distant and recent past evolutionary histories. Finally, we confirm HLA signatures indicative of migration patterns between non-native populations and their ancestral homelands, both ancient and more recent. A world map summarizing the main global HLA population relations derived from these analyses is shown in Figure 9.

The results of our analyses at the allele group level are congruent with a major role of demographic events in the shaping of the HLA profiles of human populations as previously reported using higher-resolution allelic frequencies or molecular variation (Prugnolle et al., 2005; Nunes et al., 2010; Buhler and Sanchez-Mazas, 2011; Sanchez-Mazas et al., 2012a; Riccio et al., 2013), especially for HLA class I. These patterns correlate well with the distance and main dispersal routes from the African continent as has been previously observed (Buhler and Sanchez-Mazas, 2011; Di and Sanchez-Mazas, 2011; 2014). Moreover, main geographic barriers between continental and intracontinental regions, such as the Alps in Europe (Novembre et al., 2008; Nunes et al., 2010; Sanchez-Mazas et al., 2013) or the Saharan desert in Africa, have a clear imprint on HLA profiles analyzed here as has been previously described for both HLA and other genetic systems. However, an effect of region-specific pathogen-driven selection on the shaping of HLA population profiles has also been demonstrated (Sanchez-Mazas et al., 2017a) even at the supertype level for the *HLA-B* locus (Dos Santos Francisco et al., 2015), suggesting that HLA locus-specific demographic and selective events can interact with each other in a given geographic context (Prugnolle et al., 2005).

Our results hint to a different strength of correspondence between HLA class I and class II frequency profiles and geographic location at the first-field level, suggesting that the interplay between demographic and selective pressures can have different effects for these genes. Of note, previous studies have found significant correlations between geographic distances and both class I and class II allelic and molecular frequencies, and pointed to a stronger similarity between the *HLA-B* and *HLA-DRB1* polymorphism and diversity (Solberg et al., 2008; Buhler and Sanchez-Mazas, 2011; Di and Sanchez-Mazas, 2014). However, previous work has also observed differential correlations between pathogen richness and HLA class I and class II diversities (Prugnolle et al., 2005; Sanchez-Mazas et al., 2012a), with HLA-A and HLA-B showing a positive correlation for heterozygosity and viral pathogens, in turn absent for HLA-DRB1 (Sanchez-Mazas et al., 2012a). Except for the different splitting order in the clustering and the less pronounced increases in the PCoA distances across continental groups for HLA-B, our results examining *HLA-A* and *HLA-B* independently were mostly congruent and similar to the joint analysis. This could be due to differences in the level of resolution and the methodologies applied in this work. It is however conceivable that the more stringent peptide-binding properties of class I molecules have led to the larger number of alleles (and broader repertoire of allele groups) resulting in a better and more similar representation of the geographic location of populations for class II than *HLA-DRB1* at this level of resolution. Indeed, a proposed model of joint divergent asymmetric selection operating between *HLA-A* and *HLA-B* could account for their increased power to discriminate human populations according to their pathogen histories (Buhler et al., 2016; Di et al., 2021), in particular when analyzed together.

Interestingly, the analysis of HLA barriers among populations provides additional insights in this respect. The analysis of individual loci detected in many cases conserved genetic discontinuity patterns, coupled with locus-specific discontinuity regions. For HLA class I (and thus, adaptation to and coevolution with intracellular pathogens) statistically significant barriers at the continental level were indeed found when both loci were analyzed together. Although we cannot rule out an effect of a reduced repertoire of first-field allele groups for *HLA-DRB1* in the lack of statistical confirmation of class II discontinuities, it is conceivable that once more the biological differences between both classes and their specific interaction with both demographic and infectious factors explain the differences in the strengths of genetic barrier development. Interestingly, several discontinuities become apparent when a combined class I + class II analysis is performed, suggesting that both genetic regions could be simultaneously affected by specific events in a given geographical context leading to system-wide barriers. Overall, the patterns observed here likely correspond to a mixture of immune adaptation intertwined with demographic and historical events. Since we are dealing with present-day data, our observations constitute just a snapshot of an intricate interplay of pathogen and human coevolving taking place for the past hundreds of thousands of years. Finally, it should be noted that the population sampling density in specific regions could affect the detection of statistically significant barriers, and that improved sampling could help confirm the genetic discontinuities observed.

The HLA frequency data analyzed here also reflect relatively recent as well as more ancient migration patterns. While the clustering and PCoA proximity of African- and European-descendant populations in the Americas, South Africa, and Australia with West African and North-western European populations, respectively, is not surprising, the fact that our analyses were also able to detect population relatedness between Jewish diaspora and European Romani populations and those of present day Eastern Mediterranean and South Asian regions, respectively, attest to the persistence of their original HLA signatures despite genetic drift and longer contact and gene flow with neighboring populations, as has been suggested by previous HLA (Klitz et al., 2010; Inotai et al., 2015) and non-HLA (Hammer et al., 2000; Atzmon et al., 2010; Bianco et al., 2020)-based studies. Similarly, the resemblance between HLA profiles of Polynesian, Rapa Nui, and Maori population samples to those of Taiwanese populations are further examples of the capacity of HLA data to detect these sequential, long-range migration patterns as previously noted (Friedlaender et al., 2008; Riccio et al., 2013; Ioannidis et al., 2021).

Apart from the recent and more ancient migration patterns, admixture events and gene flow are evident in the form of genetic clines in populations in intra or intercontinental contact zones (Di and Sanchez-Mazas, 2011; 2014; Hellenthal et al., 2014). Examples of these events that are evident from their HLA fingerprints in our frequency heat maps and their proximity in PCoA and clustering plots and that have been previously observed by others include the relatedness of the European Sámi with other Arctic and Siberian populations (Riccio et al., 2013; Lamnidis et al., 2018), the closeness between Egyptian and East African populations (Schuenemann et al., 2017), and the Australian-Papuan-Polynesian allele group signatures prevalent also in other OCE and SEA populations (Friedlaender et al., 2008).

The reduction in the numbers of HLA allele groups detected for AUS, OCE, NAM, and SAM, resulting in high frequencies in those groups reflect the intense population differentiation in these regions (Mack et al., 2007; Solberg et al., 2008). Previous research had observed this reduction in diversity at the allelic level (Solberg et al., 2008) and both in terms of expected heterozygosity and nucleotide diversity (Buhler and Sanchez-Mazas, 2011). This restriction is especially strong for Native Americans, and is reflected in the complex relation between geography and HLA profiles in the American continent, which seem less driven by geographical proximity, as previously observed (Nunes et al., 2010; Buhler and Sanchez-Mazas, 2011; Barquera et al., 2020b). One likely explanation for this situation in the Americas is the presence of different subsequent waves of colonization, all characterized by various degrees of strong founder effects and genetic drift resulting in high levels of population differentiation (Wang et al., 2007; Reich et al., 2012). Moreover, post-colonial Native American populations are also characterized by low effective population size, relative isolation (Barrantes, 1993), and differing degrees of European and African gene flow (Hollenbach et al., 2001; Arrieta-Bolanos et al., 2018; Barquera et al., 2020b), which further contribute to their complexity. Of note, these populations also show the highest number of private *HLA-DRB1* alleles (Gonzalez-Galarza et al., 2021), which account for a high proportion of their allele pools (Meyer et al., 2006) and are thought to be the result of selective forces acting in a context of severely reduced HLA diversity (Titus-Trachtenberg et al., 1994; Erlich et al., 1997; Hollenbach et al., 2001). In fact, a mixture of long-term balancing selection and a combination of episodes of more recent positive and balancing selection has been associated with simultaneously increasing intra-population diversity and inter-population differentiation in Native Americans (Single et al., 2020; Nunes et al., 2021).

Our study includes a unique sample of human populations and HLA frequency data providing a comprehensive view on worldwide HLA variation patterns; however some aspects remain as limitations for the approach followed. Although sufficient for identifying the main patterns of population differentiation at the global level (Di et al., 2017), the use of first-field molecular frequency data could have masked finer variation patterns characterizing specific ethnic groups or populations at higher levels of resolution (Creary et al., 2021; Nunes et al., 2022). In addition, a lack of genotypic data for many of the datasets precludes assessment of other relevant population genetics parameters (Sanchez-Mazas et al., 2012b). In particular, evidence of HWE was not used as criterion for population sample inclusion. Despite this, the majority of the populations included (61% and 63% for class I and *HLA-DRB1*, respectively) were in HWE, and only a small minority showed deviations (5% and 10%, respectively). These deviations were in most cases for one of the loci, at higher resolution, and had low statistical significance that may have disappeared if correction for multiple testing had been applied by in the original studies. Exclusion of those samples from the analyses did not impact the main observations made at the global and continental levels (not shown). Despite this, the results for samples with deviations and of those in which testing was not done/not reported by the original authors (34% and 25%, for class I and *HLA-DRB1*, respectively) should be taken with caution. An examination of whether the deviations found arise from an effect of sampling or as a consequence of a biological or demographic phenomenon are of interest but out of the scope of the present work.

Due to their significant variation across human populations (Maiers et al., 2007; Gragert et al., 2013) and their conserved nature, it is expected that HLA extended haplotype frequencies be more powerful than allele or allele group frequencies in segregating human populations (Askar et al., 2013; Hollenbach et al., 2015; Arrieta-Bolanos et al., 2018). However, a significant proportion of published HLA frequency data is still of low or intermediate resolution (Gonzalez-Galarza et al., 2021), especially for class I, and haplotype frequency data is much more scarce and subject to important technical limitations (Single et al., 2002; Eberhard et al., 2013; Nunes, 2021). Hence, the homogenization of HLA frequency data to the first field allows for the inclusion of many more datasets, while also reducing the potential confounding effects of sampling, variations in sample size, and different epochs of HLA typing. Newer third and fourth-field high-resolution HLA data obtained with next-generation sequencing methods (Creary et al., 2021; Nunes et al., 2022) will likely further refine the population comparisons based on this genetic system. Moreover, although the inclusion of as many populations from as many geographical regions was striven for, some regions are still much better represented than others due to better sampling. This is particularly important for genetically complex regions like Central Asia (Yunusbayev et al., 2015; Jeong et al., 2019), where paucity of HLA frequency data, of which we have included some recent studies (Shen et al., 2010; Hajjej et al., 2020; Wang et al., 2021), remains a major gap (Gonzalez-Galarza et al., 2021). Hence, future inclusion of additional populations from that and other underrepresented regions will likely improve the comprehensiveness of the HLA global variation picture. Lastly, because our results are based on modern data the effects of recent migration, relatively recent selection events, and the demographic history of the last five centuries cannot be completely dissected from more ancient events. With the aid of ancient DNA and paleogenomics (Quintana-Murci, 2019; Barquera and Krause, 2020), we may be able to better study true selection events and the effects of past pandemics and epidemics on the HLA profiles of human populations across time and space.

In conclusion, this large comparison of worldwide HLA first-field frequencies contributes to our understanding of the global population relations for this genetic system, confirming previous results based on high-resolution data and further demonstrating their remarkable similarity to geography, albeit with different intensities between class I and class II loci. In addition, the unique HLA dataset analyzed here also reflects specific patterns of extreme differentiation, genetic barriers and admixture, and ancient migrations and diasporas, all of which have implications for both human evolutionary studies and biomedical research.

## Data Availability

Publicly available datasets were analyzed in this study. This data can be found here: http://www.allelefrequencies.net/hla.asp.
