# Supplementary material for: An HLA map of the world: A comparison of HLA frequencies in 200 worldwide populations reveals diverse patterns for class I and class II
Source: Front Genet. 2023 Mar 23;14:866407. doi: 10.3389/fgene.2023.866407 (PMC10076764; doi:10.3389/fgene.2023.866407)
Supplement: Supplementary file 1 [file DataSheet1.docx]

Supplementary Material

# Supplementary Tables

| **Supplementary Table 1. Details of the populations samples included in the HLA class I analyses.** | | | | | |
| --- | --- | --- | --- | --- | --- |
| **Code** | **Name** | **Region^1^** | **Sample size (2N)** | **HWE^2*^** | **Reference** |
| Alb | Albanians | EUR | 320 | YES | (Sulcebe et al, 2009) |
| AmSam | American Samoans | OCE | 102 | YES | (Mack, 2007) |
| And | Spanish from Andalucía | EUR | 198 | NT | (Gonzalez-Galarza et al, 2020) |
| AndG | Spanish Roma from Andalucía | EUR | 198 | NT | (Gonzalez-Galarza et al, 2020) |
| ArgCh | Argentinian Chiriguanos | SAM | 108 | NT | (Gonzalez-Galarza et al, 2020) |
| ArgET | Argentinian Eastern Toba | SAM | 270 | YES | (Fernandez-Vina et al, 1997) |
| ArgRT | Argentinian Toba from Rosario | SAM | 172 | NT | (Gonzalez-Galarza et al, 2020) |
| ArgW | Argentinian Wichi | SAM | 92 | YES | (Fernandez-Vina et al, 1997) |
| Arm | Armenians | EUR | 8558 | YES | (Matevosyan et al, 2011) |
| Aus | Austrians | EUR | 400 | NT | (Gonzalez-Galarza et al, 2020) |
| AusAY | Australian Aborigines from Yuendumu | AUS | 382 | YES | (Gao et al, 1997) |
| AusCY | Australian Aborigines from Cape York | AUS | 206 | DEV (HLA-B) | (Gao et al, 1997) |
| AusGE | Australian Aborigines from Groote Eylandt | AUS | 150 | DEV (both) | (Gao et al, 1997) |
| AusW | Australians of European descent | AUS | 1782 | NT | (Gonzalez-Galarza et al, 2020) |
| AzoTI | Azoreans from Terceira Island | EUR | 260 | YES | (Bettencourt et al, 2016) |
| B&H | Bosnians | EUR | 268 | NT | (Gonzalez-Galarza et al, 2020) |
| Bah | Bahraini | SWA | 350 | YES | (Hajjej et al, 2020b) |
| BanDB | Bangldeshi Bangalees from Dhaka | SWA | 282 | YES | (Ali et al, 2008) |
| BasA | Basques from Arratia Valley | EUR | 166 | YES | (Sanchez-Velasco et al, 2003) |
| BasG | Basques from Guipuskoa | EUR | 200 | YES | (Comas et al, 1998) |
| Bel | Belgians | EUR | 198 | NT | (Hidajat et al, 1998) |
| BF | Burkina Faso Mossi and Rimaibe | SSA | 200 | YES | (Modiano et al, 2001) |
| BolA | Bolivian Aymara | SAM | 204 | YES | (Arnaiz-Villena et al, 2005) |
| BolQ | Bolivian Quechua | SAM | 160 | YES | (Martinez-Laso et al, 2006) |
| BraT | Brazilian Terena | SAM | 120 | NT | (Lazaro et al, 1999) |
| BuJ | Bukhara Jews | SWA | 4634 | YES | (Manor et al, 2016) |
| Bul | Bulgarians | EUR | 110 | YES | (Ivanova et al, 2002) |
| C&L | Spanish from Castile | EUR | 3880 | YES | (Alcoceba et al, 2011) |
| CAFMP | Pygmy from Central African Republic | SSA | 72 | YES | (Bruges Armas et al, 2003) |
| CamBa | Cameroon Bamileke | SSA | 154 | NT | (Torimiro et al, 2006) |
| CamBe | Cameroon Beti | SSA | 348 | NT | (Torimiro et al, 2006) |
| CamYa | Cameroon Yaounde | SSA | 184 | YES | (Ellis et al, 2000) |
| CanC | Canadian Cree | NAM | 84 | YES | (Larcombe et al, 2017) |
| CanD | Canadian Dene | NAM | 126 | YES | (Larcombe et al, 2017) |
| CapVNW | Cape Verdeans from NW island | SSA | 124 | YES | (Spinola et al, 2005) |
| CapVSE | Cape Verdeans from SE island | SSA | 124 | YES | (Spinola et al, 2005) |
| CatG | Catalonians from Girona | EUR | 176 | YES | (Comas et al, 1998) |
| ChiK | Kazakhs from China | NEA | 220 | YES | (Wang et al, 2021) |
| ChiNW | North-western Chinese | NEA | 438 | NR | (Yao et al, 2012) |
| ChiS | Chinese from Shanghai | SEA | 52532 | YES | (Du et al, 2007) |
| ChiSH | Southern Han from China | SEA | 568 | DEV (HLA-A) | (Mack, 2007) |
| ChiSW | South-western Chinese | SEA | 418 | NR | (Yao et al, 2012) |
| ChiT | Tibetans | NEA | 316 | YES | (Chen et al, 2006) |
| ChiU | Uygurs from China | SWA | 208 | YES | (Shen et al, 2010) |
| ChM | Chilean Mapuche | SAM | 208 | YES | (Rey et al, 2013b) |
| ColW | Colombian Wayu | SAM | 94 | NR | (Silvera et al, 2011) |
| CRAC | African-Caribbeans from Costa Rica | NAM | 204 | YES | (Arrieta-Bolanos et al, 2019a) |
| CRAI | Costa Rican Amerindians | NAM | 250 | YES | (Arrieta-Bolanos et al, 2019b) |
| Cro | Croatians | EUR | 300 | YES | (Mack, 2007) |
| Cyp | Cypriots | EUR | 4594 | YES | (Nunes et al, 2014) |
| CzeR | Czechs | EUR | 212 | YES* | (Gonzalez-Galarza et al, 2020) |
| Den | Danes | EUR | 152 | NT | (Jersild & Steffensen, 1997a) |
| Egy | Egyptians | NAF | 184 | YES | (Nunes et al, 2014) |
| Eng | English from Leeds | EUR | 10048 | NT | (Gonzalez-Galarza et al, 2020) |
| EqGB | Bubi, Equatorial Guinea | SSA | 202 | NT | (de Pablo et al, 1998) |
| EthJ | Ethiopian Jews | SSA | 11856 | YES | (Manor et al, 2016) |
| Far | Faroese | EUR | 100 | NT | (Jersild & Steffensen, 1997b) |
| Fin | Finns | EUR | 182 | YES | (Mack, 2007) |
| FraC | Corsicans | EUR | 200 | YES | (Grimaldi et al, 2001) |
| FraR | French from Rennes | EUR | 2000 | NT | (Gonzalez-Galarza et al, 2020) |
| FraSE | French from south-east | EUR | 260 | NT | (Gonzalez-Galarza et al, 2020) |
| Geo | Georgians | EUR | 218 | YES | (Rey et al, 2013a) |
| Ger | Germans | EUR | 22814 | NT | (Gonzalez-Galarza et al, 2020) |
| Gha | Ghanians | SSA | 262 | NR | (Norman et al, 2013) |
| GreC | Greeks from Crete | EUR | 270 | YES | (Arnaiz-Villena et al, 1999) |
| GreN | Greeks from the north | EUR | 1000 | NT | (Gonzalez-Galarza et al, 2020) |
| GrIn | Inuit from Greenland | NAM | 86 | NT | (Grunnet et al, 1996) |
| GuaM | Guatemalan Mayas | NAM | 264 | NR | (Gomez-Casado et al, 2003) |
| GuiB | Guineans | SSA | 130 | YES | (Spinola et al, 2005) |
| Hun | Hungarians | EUR | 3288 | YES | (Inotai et al, 2015) |
| HunR | Hungarian Roma | EUR | 372 | YES | (Inotai et al, 2015) |
| IndAP | Indians from Andrah-Pradesh | SWA | 222 | DEV (HLA-A) | (Mack, 2007) |
| IndM | Indians from Mumbai | SWA | 656 | YES | (Chhaya et al, 2010) |
| IndN | Northern Indians | SWA | 1174 | NT | (Gonzalez-Galarza et al, 2020) |
| InJW | Indonesians from western Java | SEA | 472 | NT | (Yuliwulandari et al, 2010) |
| IraB | Iranian Baloch | SWA | 200 | YES | (Farjadian et al, 2004) |
| IraG | Iranian Turkmen from Gorgan | SWA | 138 | YES | (Rey et al, 2015) |
| IraRB | Iranians | SWA | 31200 | NT | (Gonzalez-Galarza et al, 2020) |
| IreS | Irish | EUR | 500 | YES | (Dunne et al, 2008) |
| IrqK | Iraqui Kurds | SWA | 808 | YES | (Nunes et al, 2014) |
| IsrJ | Israeli Jews | SWA | 46000 | NT | (Gonzalez-Galarza et al, 2020) |
| IsrP | Palestinians | SWA | 330 | YES | (Gonzalez-Galarza et al, 2020) |
| ItaN | Italians North Pavia | EUR | 1370 | NT | (Gonzalez-Galarza et al, 2020) |
| ItaSa | Sardinians | EUR | 200 | YES | (Grimaldi et al, 2001) |
| ItaSi | Sicilian | EUR | 366 | YES | (Bonanno et al, 2007) |
| Jap | Japanese | NEA | 37208 | YES | (Ikeda et al, 2015) |
| JapA | Ainu | NEA | 100 | DEV (HLA-A) | (Bannai et al, 2000) |
| Jor | Jordanians | SWA | 30282 | NT | (Elbjeirami et al, 2013) |
| Kaz | Donors of Kazakh origin | SWA | 3352 | YES | (Pingel et al, 2013) |
| Ken | Kenyans | SSA | 288 | YES | (Mack, 2007) |
| KenL | Kenyans-Luo | SSA | 530 | YES | (Cao et al, 2004) |
| KenN | Kenyans-Nandi | SSA | 480 | YES | (Cao et al, 2004) |
| Leb | Lebanese | SWA | 2618 | NT | (Khansa et al, 2013) |
| LibC | Lybians-Cyrenaica | NAF | 236 | YES | (Galgani et al, 2013) |
| Mac | Macedonians | EUR | 344 | YES | (Arnaiz-Villena et al, 2001) |
| Mal | Malaysians | SEA | 2890 | DEV (HLA-A) | (Dhaliwal et al, 2007) |
| MalB | Mali Bandiagara | SSA | 276 | YES | (Cao et al, 2004) |
| MalN | Malaysian Negritos | SEA | 100 | YES | (Jinam et al, 2010) |
| Mar | Martinique | NAM | 200 | NT | (Bera et al, 2001) |
| Mau | Mauritanians | SSA | 114 | NR | (Hamed et al, 2018) |
| Mela | Melanesian from Madang and Rabaul | OCE | 362 | NT | (Gao et al, 1997) |
| MexChT | Mexican Tarahumara from Chihuahua | NAM | 88 | YES | (Garcia-Ortiz et al, 2006) |
| MexCLM | Mexican Lacandon Maya from Chiapas | NAM | 436 | DEV (both) | (Barquera et al, 2020) |
| MexMy | Mexican Mayos | NAM | 120 | YES | (Arnaiz-Villena et al, 2007) |
| MexOMt | Mexican Mixtec from Oaxaca | NAM | 206 | YES | (Hollenbach et al, 2001) |
| MexOMx | Mexican Mixe from Oaxaca | NAM | 110 | YES | (Hollenbach et al, 2001) |
| MexOt | Mexican Otomi | NAM | 144 | DEV* (both) | (Gonzalez-Galarza et al, 2020) |
| MexOZ | Mexican Zapotec from Oaxaca | NAM | 180 | YES* | (Hollenbach et al, 2001) |
| MexTH | Mexican Teenek from Huasteca region | NAM | 110 | YES | (Vargas-Alarcon et al, 2006) |
| Micro | Micronesians from Nauru and Kiribati | OCE | 222 | NT | (Gao et al, 1997) |
| Mld | Maldivians | SWA | 424 | YES | (Testi et al, 2011) |
| MonK | Mongolians (Khalkha) | NEA | 404 | YES | (Machulla et al, 2003) |
| MorC | Moroccans from Casablanca | NAF | 200 | YES | (Choukri et al, 2002) |
| MorNM | Moroccans from Nador (Metalsa) | NAF | 146 | YES | (Piancatelli et al, 2004) |
| Moz | Mozambicans | SSA | 500 | YES | (Assane et al, 2010) |
| Mya | Myanmar Bamar and Kayin | SEA | 180 | NT | (Gonzalez-Galarza et al, 2020) |
| NCab | Spanish North Cabuernigo | EUR | 190 | YES | (Sanchez-Velasco et al, 2003) |
| NCal | New Caledonians | OCE | 130 | NT | (Main et al, 2001) |
| NCant | North Cantabrians | EUR | 166 | YES | (Sanchez-Velasco et al, 2003) |
| Neth | Dutch | EUR | 2748 | YES | (Pingel et al, 2013) |
| Nig | Nigerians | SSA | 516 | NT | (Gonzalez-Galarza et al, 2020) |
| NorS | Sami from Norway | EUR | 384 | YES | (Harbo et al, 2010) |
| NZM | New Zealand Maori | OCE | 98 | YES | (Edinur et al, 2012) |
| Oman | Omanis | SWA | 236 | YES | (Middleton et al, 2000; Williams et al, 2001) |
| PakB | Pakistan Brahui | SWA | 208 | YES | (Mohyuddin et al, 2002) |
| PakP | Pakistan Pathan | SWA | 200 | YES | (Mohyuddin et al, 2002) |
| PakS | Pakistan Sindh | SWA | 202 | YES | (Mohyuddin et al, 2002) |
| PapNG | Papua-New Guineans | OCE | 262 | NT | (Main et al, 2001) |
| ParGua | Paraguayan Guaraní | SAM | 80 | YES | (Benitez et al, 2011) |
| PasV | Spanish from Pas Valley | EUR | 176 | YES | (Sanchez-Velasco et al, 2003) |
| PerA | Peruvian from Arequipa | SAM | 336 | YES | (de Pablo et al, 2000) |
| PerLC | Peruvian Lama | SAM | 166 | YES | (Moscoso et al, 2006) |
| PerTU | Peruvian Uro | SAM | 210 | YES | (Arnaiz-Villena et al, 2009) |
| Phil | Filipinos (USA) | SEA | 101228 | NT | (Gragert et al, 2013) |
| Pol | Poles | EUR | 41306 | YES | (Schmidt et al, 2011) |
| Poly | Polynesians | OCE | 448 | YES | (Edinur et al, 2012) |
| PorC | Portuguese from Central Portugal | EUR | 1124 | NT | (Gonzalez-Galarza et al, 2020) |
| PorF | Portuguese from Faro | EUR | 2484 | NT | (Gonzalez-Galarza et al, 2020) |
| PorP | Portuguese from Porto | EUR | 15874 | NT | (Gonzalez-Galarza et al, 2020) |
| Rom | Romanians | EUR | 696 | NT | (Gonzalez-Galarza et al, 2020) |
| RusAl | Russian Aleut | NEA | 208 | NT | (Arnaiz-Villena et al, 2019) |
| RusAP | Russians from Arkangelsk | EUR | 126 | NT | (Evseeva et al, 2002) |
| RusBa | Russian Bashkirs | EUR | 292 | YES | (Suslova et al, 2012) |
| RusCh | Russian Chuvash | EUR | 164 | YES | (Arnaiz-Villena et al, 2003) |
| RusKa | Russian Kalmyks | EUR | 1692 | YES | (Loginova et al, 2021) |
| RusMS | Sami from Russia (Murmansk) | EUR | 140 | NT | (Evseeva et al, 2002) |
| RusNe | Russian Nenets | NEA | 110 | NT | (Evseeva et al, 2002) |
| RusNW | Russians from the north-west | EUR | 200 | NT | (Gonzalez-Galarza et al, 2020) |
| RusSN | Russian Nivkhi | NEA | 106 | NT | (Lou et al, 1998) |
| RusSR | Russians from Samara | EUR | 5000 | NT | (Gonzalez-Galarza et al, 2020) |
| RusTu | Russian Tuva | NEA | 338 | NT | (Gonzalez-Galarza et al, 2020) |
| RusUT | Russian Tatar | EUR | 270 | YES | (Suslova et al, 2012) |
| Rwa | Rwandans | SSA | 560 | YES | (Tang et al, 2000) |
| SA | Saudis | SWA | 766 | NT | (Hajeer et al, 2009) |
| SAB | Black South Africans | SSA | 400 | YES | (Paximadis et al, 2012) |
| SAC | South Africans of European descent | SSA | 204 | YES | (Paximadis et al, 2012) |
| ScoO | Scottish from Orkney | EUR | 198 | YES | (Tonks et al) |
| SenNM | Senegalese (Madenka) | SSA | 330 | YES | (Sanchez-Mazas et al, 2000) |
| Ser | Serbians | EUR | 582 | NT | (Gonzalez-Galarza et al, 2020) |
| Sev | Spanish from Seville | EUR | 556 | NT | (Gonzalez-Galarza et al, 2020) |
| SK | South Koreans | NEA | 970 | NT | (Lee et al, 2005) |
| SL | Sri Lankan Sinhalese | SWA | 202 | NT | (Malavige et al, 2007) |
| Slk | Slovakians | EUR | 8468 | NT | (Gonzalez-Galarza et al, 2020) |
| Slo | Slovenians | EUR | 260 | NT | (Smigoc Schweiger et al, 2014) |
| Sp-Can | Spanish-BMD-Canarias | EUR | 1218 | YES | (Romon et al, 2016) |
| STIF | Sao Tome Islanders (Forro) | SSA | 132 | DEV (HLA-B) | (Saldanha, 2009) |
| Sud | Sudanese | SSA | 400 | NT | (Gonzalez-Galarza et al, 2020) |
| SudN | Sudanese Nuba | SSA | 92 | NT | (Gonzalez-Galarza et al, 2020) |
| SweNS | Sami from Sweden | EUR | 308 | YES | (Johansson et al, 2008) |
| Swe | Swedish | EUR | 1932 | NT | (Gonzalez-Galarza et al, 2020) |
| Swi | Swiss from Bern | EUR | 1440 | YES | (Buhler et al, 2012) |
| Syr | Syrians | SWA | 328 | YES | (Nunes et al, 2014) |
| TaiA | Taiwanese Aborigines (Atayal) | SEA | 212 | YES* | (Gonzalez-Galarza et al, 2020) |
| Tan | Tanzanians | SSA | 424 | YES | (Koehler et al, 2010) |
| Thai | Thai | SEA | 33614 | NT | (Kupatawintu et al, 2010) |
| TriA | Trinidad Africans | NAM | 134 | NT | (Gonzalez-Galarza et al, 2020) |
| TunB | Tunisian Berbers | NAF | 210 | YES | (Hajjej et al, 2011) |
| TunG | Tunisians from Ghannouch | NAF | 164 | YES | (Hajjej et al, 2006a) |
| TunT | Tunisians from Tunis | NAF | 208 | YES | (Hajjej et al, 2006b) |
| Tur | Turks (DKMS) | EUR | 9712 | YES | (Pingel et al, 2013) |
| UAE | United Arab Emirates | SWA | 746 | NT | (Gonzalez-Galarza et al, 2020) |
| UgaK | Ugandan from Kampala | SSA | 350 | YES | (Kijak et al, 2009) |
| UK | British | EUR | 202 | YES | (Boyton et al, 2008) |
| Ukr | Ukrainians from Kazakhstan | SWA | 210 | YES | (Hajjej et al, 2021) |
| USAA | African Americans | NAM | 4822 | YES | (Maiers et al, 2007) |
| USAAI | Arizona Gila River Amerindian | NAM | 984 | YES | (Williams et al, 2009) |
| USAAO | African Americans | NAM | 3020 | YES | (Leffell et al, 2007) |
| USAN | Alaska Yupik | NAM | 504 | YES | (Leffell et al, 2002) |
| USAPi | Pima from Arizona | NAM | 200 | YES | (Mack, 2007) |
| USCau | European-descendants from USA | NAM | 17050 | YES | (Leffell et al, 2007) |
| USHO | Okinawans from Hawaii | OCE | 212 | YES* | (Mack, 2007) |
| USHPI | USA Hawaiian and Pacific Islanders | OCE | 22998 | NT | (Gragert et al, 2013) |
| USSDS | South Dakota Sioux | NAM | 604 | YES | (Leffell et al, 2004) |
| Uzb | Uzbeks from Kazakhstan | SWA | 84 | YES | (Hajjej et al, 2020a) |
| VenPMB | Venezuelan Bari | SAM | 110 | YES | (Mack, 2007) |
| VietH | Vietnamese | SEA | 340 | YES | (Hoa et al, 2008) |
| Wal | Welsh | EUR | 79958 | NT | (Gonzalez-Galarza et al, 2020) |
| YeJ | Yemen Jews | SWA | 31084 | DEV (both) | (Manor et al, 2016) |
| ZamL | Zambians from Lusaka | SSA | 88 | YES | (Cao et al, 2004) |
| ZimHS | Zimbabwe Harare Shona | SSA | 460 | YES | (Mack, 2007) |
|  |  |  |  |  |  |
| **Total** | **200** |  | **712,462** |  |  |
| ^1^Geographic region where each population is located. AUS, Australia; EUR, Europe; NAF, North Africa; NAM, North America; NEA, Northeast Asia; OCE, Oceania; SAM, South America; SEA, Southeast Asia; SWA, Southwest Asia; SSA, Sub-Saharan Africa.^2^HWE, Hardy-Weinberg equilibrium testing results as reported by the authors of the original study or tested in this study. DEV, deviation (locus); NR, test included in Methods but results not reported; NT, not tested; YES, both HLA-A and HLA-B in HWE. *HWE tested in this work. | | | | | |

| **Supplementary Table 2. Details of the populations samples included in the HLA-DRB1 analyses.** | | | | | |
| --- | --- | --- | --- | --- | --- |
| **Code** | **Name** | **Region** | **Sample size (2N)** | **HWE^2*^** | **Reference** |
| Al | Albanians | EUR | 320 | YES | (Sulcebe et al., 2009) |
| Dz | Algerians | NAF | 212 | NT | (Arnaiz-Villena et al., 1995) |
| ArE | Argentina Gran Chaco Eastern Toba | SAM | 270 | YES | (Cerna et al., 1993) |
| ArM | Argentina Gran Chaco Mataco Wichi | SAM | 98 | YES | (Cerna et al., 1993) |
| ArP | Argentina Gran Chaco Western Toba Pilaga | SAM | 38 | YES | (Cerna et al., 1993) |
| ArT | Argentina Rosario Toba | SAM | 172 | NT | (Gonzalez-Galarza et al., 2020) |
| Am | Armenians | EUR | 200 | YES | (Matevosyan et al., 2011) |
| AuC | Australia Cape York Peninsula Aborigines | AUS | 206 | YES* | (Gao et al., 2007) |
| AuK | Australia Kimberley Aborigines | AUS | 82 | YES* | (Gao et al., 2007) |
| AuY | Australia Yuendumu Aborigines | AUS | 382 | NT | (Gao et al., 2007) |
| At | Austrians | EUR | 3396 | YES | (Pingel et al., 2013) |
| AzC | Azoreans from Central Island | EUR | 118 | YES | (Gonzalez-Galarza et al., 2020) |
| AzO | Azoreans from Oriental Island | EUR | 86 | YES | (Gonzalez-Galarza et al., 2020) |
| AzT | Azoreans from Terceira Island | EUR | 260 | YES | (Gonzalez-Galarza et al., 2020) |
| Ber | Aleuts from Bering strait | NEA | 170 | YES | (Moscoso et al., 2008) |
| Be2 | Aleuts from Bering strait 2 | NEA | 208 | NT | (Arnaiz-Villena et al., 2019) |
| BoA | Bolivian Aymara | SAM | 204 | YES | (Arnaiz-Villena et al., 2005) |
| BoQ | Bolivian Quechua | SAM | 160 | YES | (Martinez-Laso et al., 2006) |
| Ba | Bosnians | EUR | 2056 | YES | (Pingel et al., 2013) |
| BrK | Guarani-Kaiowá | SAM | 288 | YES* | (Petzl-Erler and Tsuneto, 2007) |
| BrN | Guarani-Ñandeva | SAM | 106 | YES* | (Petzl-Erler and Tsuneto, 2007) |
| BrT | Brazil Terena | SAM | 120 | NT | (Lázaro et al., 1999) |
| Bg | Bulgarians | EUR | 110 | NT | (Ivanova et al., 2002) |
| BfF | Burkina Faso Fulani | SSA | 98 | NT | (Modiano et al., 2001) |
| BfM | Burkina Faso Mossi | SSA | 106 | NT | (Modiano et al., 2001) |
| BfR | Burkina Faso Rimaibe | SSA | 94 | NT | (Modiano et al., 2001) |
| CaC | Canada Chipewyan | NAM | 50 | YES | (Single et al., 2020) |
| CaR | Canada Cree | NAM | 36 | YES | (Single et al., 2020) |
| CaO | Canada Ojibwa | NAM | 32 | YES | (Single et al., 2020) |
| CvN | Cape Verdeans from NW Island | SSA | 124 | DEV | (Spínola et al., 2005) |
| CvS | Cape Verdeans from SE Island | SSA | 124 | DEV | (Spínola et al., 2005) |
| Cm | Camerooneans | SSA | 252 | NT | (Pimtanothai et al., 2001) |
| Cf | Central African Republic pygmy | SSA | 186 | YES | (Renquin et al., 2001) |
| ClM | Chile Mapuche | SAM | 208 | YES | (Rey et al., 2013) |
| ClH | Chile Huiliche | SAM | 40 | YES | (Single et al., 2020) |
| Cn | Chinese | NEA | 2564 | YES | (Pingel et al., 2013) |
| CnH | Chinese Han | SEA | 528 | YES | (Trachtenberg et al., 2007) |
| CnHG | Chinese Han (1000G Project) | SEA | 200 | YES* | (Gourraud et al., 2014) |
| CnB | China Beijing Shijiazhuang Tianjian Han | NEA | 1236 | NT | (Yang et al., 2006) |
| CnM | China Guangxi Region Maonan | SEA | 216 | NT | (Ogata et al., 2007) |
| CnJ | China Jiangsu Han | NEA | 6476 | YES | (Qin Qin et al., 2011) |
| CnL | China Lisu | SEA | 222 | YES | (Chen et al., 2007) |
| CnW | China Wuhan | SEA | 240 | NT | (Ferencik et al., 1998) |
| CnY | China Yunnan Bulang | SEA | 232 | YES | (Shi et al., 2010) |
| CoI | Colombia Inga | SAM | 32 | YES | (Single et al., 2020) |
| CoA | Colombia Arara | SAM | 34 | YES | (Single et al., 2020) |
| CoT | Colombia Tarapaca | SAM | 38 | YES | (Single et al., 2020) |
| CoU | Colombia Waunana | SAM | 40 | YES | (Single et al., 2020) |
| CoW | Colombia Wayu | SAM | 96 | NT | (Silvera et al., 2011) |
| CRC | Costa Rica Cabécar | NAM | 38 | YES | (Single et al., 2020) |
| CRG | Costa Rica Ngäbe | NAM | 36 | YES | (Single et al., 2020) |
| Hr | Croatians | EUR | 4114 | YES | (Pingel et al., 2013) |
| Cz | Czech Republic | EUR | 212 | DEV* | (Ivaskova and Bendukidze, 2007) |
| Cz2 | Czech Republic | EUR | 10198 | NT | (Gonzalez-Galarza et al., 2020) |
| En | NW England | EUR | 596 | YES | (Alfirevic et al., 2012) |
| FiG | Finland 1000G | EUR | 200 | YES* | (Gourraud et al., 2014) |
| Fr | France | EUR | 2812 | YES | (Pingel et al., 2013) |
| Geo | Georgians | EUR | 160 | NT | (Sánchez-Velasco and Leyva-Cobián, 2001) |
| De | Germany | EUR | 26772 | NT | (Mullër et al., 2003) |
| De2 | Germany | EUR | 79378 | NT | (Gonzalez-Galarza et al., 2020) |
| GhA | Ghana Akan | SSA | 858 | NT | (Yamazaki et al., 2011) |
| GtM | Guatemala Maya | NAM | 264 | NT | (Gómez-Casado et al., 2003) |
| Gw | Guinea Bissau | SSA | 130 | YES | (Spínola et al., 2005) |
| Gr | Greece | EUR | 3788 | YES | (Pingel et al., 2013) |
| Hk | Hong Kong | SEA | 15190 | DEV | (Kwok et al., 2016) |
| InA | India Andhra Pradesh Golla | SWA | 222 | DEV* | (Mack et al., 2007b) |
| InM | India Mumbai Maratha | SWA | 182 | NT | (Shankarkumar et al., 2010) |
| InN | India North pop 2 | SWA | 144 | YES | (Rajalingam et al., 2002) |
| IdW | Indonesia Java Western | SEA | 472 | NT | (Yuliwulandari et al., 2010) |
| IdJ | Indonesia Javanese | SEA | 402 | NT | (Yuliwulandari et al., 2010) |
| Ir | Iran Baloch | SWA | 200 | DEV | (Farjadian et al., 2004) |
| Iq | Iraq | SWA | 418 | NT | (Gonzalez-Galarza et al., 2020) |
| IeN | Ireland (North) | EUR | 2000 | YES | (Middleton et al., 2000) |
| IeS | Ireland (South) | EUR | 500 | NT | (Dunne et al., 2008) |
| IlE | Israel Ethiopian Jew | SSA | 11856 | YES | (Manor et al., 2016) |
| IlJ | Israel Ashkenazi Jews | SWA | 9250 | YES | (Manor et al., 2016) |
| IlY | Israel Yemen Jews | SWA | 31084 | DEV | (Manor et al., 2016) |
| It | Italy | EUR | 2318 | YES | (Pingel et al., 2013) |
| ItG | Italy Tuscany (1000G Project) | EUR | 180 | YES* | (Gourraud et al., 2014) |
| Jp | Japanese | NEA | 742 | NT | (Saito et al., 2000) |
| JpA | Japan Ainu | NEA | 100 | NT | (Bannai et al., 2000) |
| JpG | Japan Tokio (1000G Project) | NEA | 362 | YES* | (Gourraud et al., 2014) |
| Jo | Jordan Amman | SWA | 292 | NT | (Sánchez-Velasco et al., 2001) |
| KeG | Kenya Luhya (1000G Project) | SSA | 180 | YES* | (Gourraud et al., 2014) |
| KeL | Kenya Luo | SSA | 200 | YES | (Arlehamn et al., 2017) |
| Ly | Libya Cyrenaica | NAF | 236 | YES | (Galgani et al., 2013) |
| Mk | Macedonia | EUR | 344 | YES | (Arnaiz-Villena et al., 2001a) |
| PtM | Portuguese from Madeira | EUR | 346 | YES | (Arnaiz-Villena et al., 2009b) |
| My | Malaysians | SEA | 2890 | YES | (Dhaliwal et al., 2007) |
| MyJ | Malaysia Jelebu Temuan | SEA | 50 | YES | (Jinam et al., 2010) |
| MyK | Malaysia Kedah Baling Kensiu | SEA | 50 | YES | (Jinam et al., 2010) |
| MyP | Malaysia Perak Grik Jehai | SEA | 50 | YES | (Jinam et al., 2010) |
| MyS | Malaysia Sarawak Bau Bidayuh | SEA | 50 | YES | (Jinam et al., 2010) |
| Ml | Doggon from Bandiagara, Mali | SSA | 276 | YES* | (Cao et al., 2007) |
| MxL | Mexico Lacandon | NAM | 436 | DEV | (Barquera et al., 2020) |
| MxM | Mexico Mayos | NAM | 120 | YES | (Arnaiz-Villena et al., 2007) |
| MxA | Mexico Mazatecan | NAM | 178 | YES | (Arnaiz-Villena et al., 2000) |
| MxI | Mexico Mixe | NAM | 110 | DEV | (Hollenbach et al., 2001) |
| MxX | Mexico Mixtec | NAM | 206 | DEV | (Hollenbach et al., 2001) |
| MxN | Mexico Nahuas | NAM | 170 | YES | (Vargas-Alarcon et al., 2007) |
| MxR | Mexico Rarámuri | NAM | 88 | YES | (García-Ortiz et al., 2006) |
| MxS | Mexico Seri | NAM | 68 | YES* | (Infante et al., 2007) |
| MxT | Mexico Teenek | NAM | 110 | NT | (Vargas-Alarcon et al., 2003) |
| Mx2 | Mexico Teenek 2 | NAM | 106 | NT | (Gonzalez-Galarza et al., 2020) |
| MxZ | Mexico Zapotec | NAM | 180 | DEV | (Hollenbach et al., 2001) |
| Mn | Mongolia | NEA | 170 | NT | (Munkhbat et al., 1997) |
| MnK | Mongolia Khalkha | NEA | 404 | YES | (Tanaka et al., 1997) |
| MnO | Mongolia Oold | NEA | 208 | YES | (Machulla et al., 2003) |
| MaM | Moroccans from Metalsa | NAF | 200 | YES* | (Piancatelli et al., 2007) |
| MaC | Morocco Atlantic Coast Chaouya | NAF | 196 | YES | (Canossi et al., 2010) |
| Ma | Morocco | NAF | 192 | YES | (Gómez-Casado et al., 2000) |
| Mz | Mozambique | SSA | 404 | YES | (Assane et al., 2010) |
| MmK | Myanmar Kayin | SEA | 88 | NT | (Gonzalez-Galarza et al., 2020) |
| MmB | Myanmar Bamar | SEA | 92 | NT | (Gonzalez-Galarza et al., 2020) |
| Nl | Netherlands | EUR | 2748 | YES | (Pingel et al., 2013) |
| Nc | New Caledonia | OCE | 130 | NT | (Gao et al., 1992) |
| Nc2 | New Caledonia 2 | OCE | 108 | YES | (Maitland et al., 2004) |
| NzM | New Zealand Maori | OCE | 92 | DEV | (Edinur et al., 2013) |
| NzP | New Zealand Polynesian | OCE | 42 | DEV | (Edinur et al., 2013) |
| Ng | Nigerians | SSA | 548 | NT | (Steiner et al., 2009) |
| NgG | Nigeria Yoruba 1000G | SSA | 178 | YES* | (Gourraud et al., 2014) |
| NoS | Norway Sámi | EUR | 400 | YES | (Harbo et al., 2010) |
| No | Norway | EUR | 1152 | DEV | (Harbo et al., 2010) |
| Pk | Pakistan Parsi | SWA | 182 | NT | (Mohyuddin and Mehdi, 2005) |
| Ps | Palestinians | SWA | 330 | YES | (Arnaiz-Villena et al., 2001b) |
| Pg | Papua New Guinea East New Britain Rabaul | OCE | 120 | NT | (Gao et al., 1992) |
| PgH | Papua New Guinea Highlanders | OCE | 184 | DEV* | (Mack et al., 2007a) |
| Py | Paraguay Guaraní | SAM | 80 | YES | (Benitez et al., 2011) |
| PeL | Peru Lama | SAM | 166 | YES | (Moscoso et al., 2006) |
| PeU | Peru Titikaka Lake Uro | SAM | 210 | YES | (Arnaiz-Villena et al., 2009a) |
| Ph | Philippines Ivatan | SEA | 100 | YES* | (Chu et al., 2007a) |
| PhL | Filipino from Luzon Island, Philippines | SEA | 188 | YES* | (Erlich et al., 2007) |
| Pl | Poland | EUR | 41306 | YES | (Pingel et al., 2013) |
| Pt | Portugal | EUR | 2352 | YES | (Pingel et al., 2013) |
| Ro | Romania | OCE | 2468 | YES | (Pingel et al., 2013) |
| Rn | Rapanui | EUR | 96 | NT | (Thorsby, 2012) |
| RuB | Russia Bashkir | EUR | 292 | YES | (Suslova et al., 2012) |
| RuC | Russia Chuvash | EUR | 164 | YES | (Arnaiz-Villena et al., 2003) |
| RuU | Russia South Ural | EUR | 414 | YES | (Suslova et al., 2012) |
| RuA | Russia Tartar | EUR | 270 | YES | (Arnaiz-Villena et al., 2003) |
| RuK | Russia Buryats | NEA | 300 | YES* | (Gonzalez-Galarza et al., 2020) |
| RuT | Russia Tuva | NEA | 380 | YES | (Martinez-Laso et al., 2001) |
| RuT2 | Russia Tuva pop 2 | NEA | 338 | NT | (Begovich et al., 2001) |
| Rw | Rwanda | SSA | 560 | YES | (Tang et al., 2000) |
| Ws | Samoans | OCE | 58 | YES | (Severson et al., 2007) |
| StM | Sao Tome Angolar | SSA | 64 | YES | (Saldanha et al., 2009) |
| StF | Sao Tome Forro | SSA | 132 | YES | (Saldanha et al., 2009) |
| SaG | Saudi Arabia Guraiat and Hail | SWA | 426 | NT | (Gonzalez-Galarza et al., 2020) |
| Sn | Senegal | SSA | 224 | NT | (Andrien and Dupont, 2004) |
| Sg | Singapore Riau Malay | SEA | 264 | NT | (Mack et al., 2007c) |
| ZaB | South Africa Black | SSA | 400 | YES | (Paximadis et al., 2012) |
| ZaC | South Africa Caucasian | SSA | 204 | DEV | (Paximadis et al., 2012) |
| ZaZ | Zulu from South Africa | SSA | 176 | YES | (Paximadis et al., 2012) |
| Kr | South Korea | NEA | 970 | NT | (Lee et al., 2005) |
| Kr2 | South Korea 2 | NEA | 8256 | YES | (Huh et al., 2013) |
| Es | Spain | EUR | 2214 | YES | (Pingel et al., 2013) |
| EsI | Spain Ibiza | EUR | 176 | YES | (Crespí et al., 2002) |
| Esj | Spain Majorcan Jews | EUR | 206 | YES | (Crespí et al., 2002) |
| EsM | Spain Murcia | EUR | 346 | YES | (Muro et al., 2001) |
| EsC | Spain North Cabuérnigo | EUR | 190 | YES | (Sánchez-Velasco et al., 2003) |
| EsP | Spain Pas Valley | EUR | 176 | YES | (Sánchez-Velasco et al., 2003) |
| Sd | Sudanese | SSA | 400 | NT | (Gonzalez-Galarza et al., 2020) |
| Se | Sweden | EUR | 1932 | NT | (Gonzalez-Galarza et al., 2020) |
| SNS | Sweden Northern Sámi | EUR | 308 | YES | (Johansson et al., 2008) |
| SSS | Sweden Southern Sámi | EUR | 260 | YES | (Johansson et al., 2008) |
| TwA | Taiwan Ami | SEA | 196 | YES* | (Chu et al., 2007b) |
| TwM | Taiwan Minnan | SEA | 204 | YES* | (Chu et al., 2007b) |
| TwC | Taiwan Tzu Chi | SEA | 1420 | YES | (Wen et al., 2008) |
| TwT | Taiwan Atayal | SEA | 212 | YES* | (Chu et al., 2007b) |
| TwB | Taiwan Bunun | SEA | 202 | YES* | (Chu et al., 2007b) |
| TwH | Taiwan Hakka | SEA | 110 | YES* | (Chu et al., 2007b) |
| TwP | Taiwan Puyuma | SEA | 100 | YES* | (Chu et al., 2007b) |
| TwS | Taiwan Saisiat | SEA | 102 | YES* | (Chu et al., 2007b) |
| Th | Thailand | SEA | 284 | NT | (Gonzalez-Galarza et al., 2020) |
| Th2 | Thailand 2 | SEA | 33614 | NT | (Kupatawintu et al., 2010) |
| Tn | Tunisia | NAF | 164 | YES | (Hajjej et al., 2006) |
| TnG | Tunisia Gabes | NAF | 190 | YES | (Hajjej et al., 2011) |
| Tr | Turkey | EUR | 9712 | DEV | (Pingel et al., 2013) |
| Ae | UAE | SWA | 596 | NT | (Santhosh et al., 2004) |
| Ug | Uganda | SSA | 206 | NT | (Okello et al., 2014) |
| UK | UK | EUR | 2086 | YES | (Pingel et al., 2013) |
| UKG | UK (1000G Project) | EUR | 192 | YES* | (Gourraud et al., 2014) |
| USG | USA Gila River Native American | NAM | 984 | YES | (Williams et al., 2009) |
| USN | USA Navajo | NAM | 86 | YES | (Williams et al., 2009) |
| USP | USA Pima | NAM | 6000 | NT | (Gonzalez-Galarza et al., 2020) |
| USS | USA Sioux | NAM | 604 | DEV | (Leffell et al., 2004) |
| UtG | USA Utah (1000G Project) | NAM | 222 | YES* | (Gourraud et al., 2014) |
| USY | USA Yupik | NAM | 504 | YES | (Leffell et al., 2002) |
| VuA | Vanuatu Aniwa | OCE | 168 | DEV | (Maitland et al., 2004) |
| VuF | Vanuatu Futuna | OCE | 164 | DEV | (Maitland et al., 2004) |
| VuP | Vanuatu Paama | OCE | 188 | YES | (Maitland et al., 2004) |
| VuS | Vanuatu Santo | OCE | 584 | YES | (Maitland et al., 2004) |
| VuT | Vanuatu Tanna | OCE | 256 | DEV | (Maitland et al., 2004) |
| VeY | Venezuela Yucpa | SAM | 146 | YES | (Layrisse et al., 2001) |
| Vn | Vietnam | SEA | 340 | YES | (Hoa et al., 2008) |
| Zm | Zambia | SSA | 584 | YES | (Tang et al., 2004) |
| Zw | Zimbabwe Harare Shona | SSA | 460 | YES* | (Louie et al., 2007) |
|  |  |  |  |  |  |
| **Total** | **197** |  | **370,794** |  |  |
| ^1^Geographic region where each population is located. AUS, Australia; EUR, Europe; NAF, North Africa; NAM, North America; NEA, Northeast Asia; OCE, Oceania; SAM, South America; SEA, Southeast Asia; SWA, Southwest Asia; SSA, Sub-Saharan Africa. .^2^HWE, Hardy-Weinberg equilibrium testing results as reported by the authors of the original study or tested in this study. DEV, deviation; NT, not tested; YES, HLA-DRB1 in HWE. *HWE tested in this work. | | | | | |

| **Supplementary Table 3. Geographic coordinates used for the *Barrier* analyses of the African continent (Figure 8a).** | | | |
| --- | --- | --- | --- |
| **Population** | **Code** | **Latitude** | **Long_mod_^1^** |
| Algeria (n = 106) | 1 | 36.75 | -147.00 |
| Azores Central Islands (n = 59) | 2 | 38.61 | -177.00 |
| Azores Oriental Islands (n = 43) | 3 | 38.06 | -175.00 |
| Azores Terceira Islands (n = 130) | 4 | 38.41 | -179.00 |
| Burkina Faso Fulani (n = 49) | 5 | 12.67 | -150.00 |
| Burkina Faso Mossi (n = 53) | 6 | 12.68 | -150.00 |
| Burkina Faso Rimaibe (n = 47) | 7 | 12.69 | -151.00 |
| Cape Verde NW (n = 62) | 8 | 15.28 | -174.00 |
| Cape Verde SE (n = 62) | 9 | 14.90 | -174.00 |
| Cameroon (n_CI_ = 548, n_CII_ = 126) | 10 | 3.84 | -139.00 |
| Central African Republic (n= 36) | 11 | 4.36 | -131.00 |
| Ghana Akan (n=429) | 12 | 6.08 | -150.00 |
| Guinea Bissau (n = 65) | 13 | 11.80 | -165.00 |
| Israel Ethiopian Jews (n=5928) | 14 | 8.98 | -111.00 |
| Kenya Luhya 1000G (n = 90) | 15 | -1.29 | -113.00 |
| Kenya Luo (n=100) | 16 | -0.08 | -115.00 |
| Libya Cyrenaica (n = 118) | 17 | 32.09 | -130.00 |
| Madeira (n = 185) | 18 | 32.66 | -167.00 |
| Doggon from Bandiagara, Mali (n= 138) | 19 | 12.63 | -158.00 |
| Metalsa from Morocco (n=100) | 20 | 34.75 | -154.00 |
| Morocco Atlantic Coast Chaouya  (n = 98) | 21 | 35.16 | -155.00 |
| Morocco (n = 96) | 22 | 33.23 | -159.00 |
| Mozambique (n = 202) | 23 | -25.90 | -117.00 |
| Nigeria (n = 274) | 24 | 9.07 | -143.00 |
| Nigeria Yoruba 1000G (n = 89) | 25 | 7.37 | -146.00 |
| Rwanda (n = 280) | 26 | -1.90 | -120.00 |
| Sao Tome Angolar (n = 32) | 27 | 0.12 | -143.00 |
| Sao Tome Forro (n = 66) | 28 | 0.33 | -143.00 |
| Senegal (n_CI_ = 165, n_CII_ = 112) | 29 | 14.49 | -164.00 |
| South Africa Black (n = 200) | 30 | -25.70 | -122.00 |
| Zulu from South Africa (n=199; n=88) | 31 | -28.50 | -119.00 |
| Sudan Mixed (n = 200) | 32 | 15.50 | -117.00 |
| Tunisia (n = 82) | 33 | 33.94 | -140.00 |
| Tunisia Gabes (n = 95) | 34 | 33.88 | -140.00 |
| Uganda (n_CI_ = 175; n_CII_ = 103) | 35 | 0.03 | -117.00 |
| Zambia (n_CI_ = 44; n_CII_ = 292) | 36 | -15.40 | -122.00 |
| Zimbabwe Harare Shona (n = 230) | 37 | -17.80 | -119.00 |
| ^1^ Longitude modified as explained in Materials and Methods. | | | |

| **Supplementary Table 4. Geographic coordinates used for the *Barrier* analyses in Eurasia (Figure 8b).** | | | |
| --- | --- | --- | --- |
| **Population** | **Code** | **Latitude** | **Long_mod_^1^** |
| Albania (n = 160) | 1 | 41.33 | -130.18 |
| Armenia (n = 100) | 2 | 40.18 | -105.51 |
| Austria (n = 1698) | 3 | 48.20 | -133.63 |
| Bosnia (n = 1028) | 4 | 43.85 | -131.61 |
| Bulgaria (n = 55) | 5 | 42.69 | -126.68 |
| China (n = 1282) | 6 | 39.97 | -33.60 |
| China Han (n = 264) | 7 | 23.10 | -36.75 |
| China Han 1000G (n = 100) | 8 | 30.57 | -35.73 |
| China Beijing Shijiazhuang Tianjian Han (n = 618) | 9 | 39.90 | -33.59 |
| China Guangxi Region Maonan (n = 108) | 10 | 23.80 | -41.02 |
| China Jiangsu Han (n = 3238) | 11 | 21.95 | -49.55 |
| China Lisu (n = 111) | 12 | 24.50 | -48.50 |
| China Wuhan (n=121) | 13 | 30.75 | -35.75 |
| China Yunnan Bulang (n = 116) | 14 | 32.90 | -30.20 |
| Croatia (n = 2057) | 15 | 45.81 | -134.02 |
| Czech Republic (n = 106) | 16 | 50.07 | -135.57 |
| Czech Republic (n = 5099) | 17 | 50.08 | -135.47 |
| NW Eng (n = 298) | 18 | 53.40 | -152.99 |
| Finland 1000G (n = 100) | 19 | 60.16 | -125.07 |
| France (n = 1406) | 20 | 48.85 | -147.65 |
| Georgia (n = 80) | 21 | 43.03 | -107.32 |
| Germany (n = 13386) | 22 | 52.52 | -136.60 |
| Germany (n = 39689) | 23 | 52.53 | -140.95 |
| Greece (n = 1894) | 24 | 37.97 | -126.28 |
| Hong Kong (N=7595) | 25 | 22.25 | -35.83 |
| India Andhra Pradesh Golla (n = 111) | 26 | 17.08 | -71.92 |
| India Mumbai Maratha (n = 91) | 27 | 18.97 | -77.18 |
| India North pop 2 (n = 72) | 28 | 31.67 | -74.42 |
| Indonesia Java Western (n = 236) | 29 | -7.09 | -42.34 |
| Indonesia Javanese (n = 201) | 30 | -6.13 | -43.83 |
| Iran Baloch (n = 100) | 31 | 26.22 | -84.94 |
| Iraq (n = 209) | 32 | 36.16 | -106.02 |
| Ire N (n = 1000) | 33 | 54.57 | -155.98 |
| Ire S (n = 250) | 34 | 53.34 | -156.26 |
| Italy (n = 1159) | 35 | 41.90 | -137.51 |
| Italy Tuscany 1000G (n = 90) | 36 | 41.91 | -137.50 |
| Israel Ashkenazi Jews (N=4625) | 37 | 32.13 | -115.13 |
| Israel Yemen Jews (N=4625) | 38 | 15.34 | -105.80 |
| Japan (n = 371) | 39 | 35.68 | -10.31 |
| Japan Ainu (n = 50) | 40 | 43.06 | -8.66 |
| Japan Tokio 1000G (n = 181) | 41 | 35.69 | -10.30 |
| Jordan Amman (n = 146) | 42 | 31.94 | -114.08 |
| Macedonia (n = 172) | 43 | 41.99 | -128.58 |
| Malaysia (n = 1445) | 44 | 3.13 | -48.30 |
| Malaysia Jelebu Temuan (n = 25) | 45 | 2.93 | -47.92 |
| Malaysia Kedah Baling Kensiu (n = 25) | 46 | 6.11 | -49.64 |
| Malaysia Perak Grik Jehai (n = 25) | 47 | 4.59 | -48.91 |
| Malaysia Sarawak Bau Bidayuh (n = 25) | 48 | 1.55 | -39.65 |
| Mongolia (n = 85) | 49 | 49.61 | -48.00 |
| Mongolia Khalkha | 50 | 47.92 | -43.12 |
| Mongolia Oold | 51 | 48.00 | -54.00 |
| Myanmar Kayin (n = 44) | 52 | 17.00 | -52.25 |
| Myanmar Bamar (n = 46) | 53 | 19.75 | -52.20 |
| Netherlands (n = 1374) | 54 | 52.37 | -145.11 |
| Norway Sami (n = 200) | 55 | 69.47 | -124.49 |
| Norway (n = 576) | 56 | 59.91 | -139.25 |
| Pakistan Parsi (n = 91) | 57 | 24.86 | -83.00 |
| Palestina (n = 165) | 58 | 31.50 | -115.55 |
| Philippines Ivatan (n = 50) | 59 | 20.44 | -28.03 |
| Filipino from Luzon Island, Philippines (n=94) | 60 | 16.56 | -28.74 |
| Poland (n = 20 653) | 61 | 52.22 | -128.99 |
| Portugal (n = 1176) | 62 | 38.72 | -159.13 |
| Romania (n = 1234) | 63 | 44.42 | -123.90 |
| Russia Bashkir (n = 146) | 64 | 55.16 | -88.57 |
| Russia Chuvash (n = 82) | 65 | 55.55 | -103.08 |
| Russia S Ural (n = 207) | 66 | 54.53 | -89.67 |
| Russia Tartar (n = 135) | 67 | 55.18 | -99.28 |
| Russia Buryats | 68 | 54.27 | -31.73 |
| Russia Tuva (n = 190) | 69 | 51.88 | -54.38 |
| Russia Tuva pop 2  (n = 169) | 70 | 51.89 | -54.37 |
| Saudi Arabia Guraiat and Hail (n = 213) | 71 | 27.52 | -108.32 |
| Singapore Riau Malay (n = 132) | 72 | 1.35 | -46.14 |
| South Korea (n = 485) | 73 | 37.56 | -23.03 |
| South Korea (n = 4128) | 74 | 37.66 | -22.92 |
| Spain (n = 1107) | 75 | 40.41 | -153.70 |
| Spain Ibiza (n = 88) | 76 | 38.90 | -148.58 |
| Spain Maj Jews (n = 103) | 77 | 39.56 | -147.35 |
| Spain Murcia (n = 173) | 78 | 37.99 | -151.13 |
| Spain N Cabuer (n = 95) | 79 | 43.10 | -153.98 |
| Spain Pas Valley (n = 88) | 80 | 43.15 | -152.81 |
| Sweden (n = 966) | 81 | 59.32 | -131.94 |
| Sweden Northern Sami (n = 154) | 82 | 66.83 | -129.61 |
| Sweden Southern Sami (n = 130) | 83 | 60.33 | -133.49 |
| Taiwan Ami (n = 98) | 84 | 23.00 | -28.99 |
| Taiwan Bunun (n = 101) | 85 | 23.77 | -28.95 |
| Taiwan Tzu Chi (n = 710) | 86 | 23.77 | -28.97 |
| Taiwan Hakka (n = 55) | 87 | 25.08 | -28.92 |
| Taiwan Minnan (n = 102) | 88 | 24.00 | -28.98 |
| Taiwan Puyuma (n = 50) | 89 | 23.00 | -29.00 |
| Saisiat from Wufen / Nanchuang, Taiwan (n=51) | 90 | 24.58 | -28.99 |
| Taiwan Atayal (n = 106) | 91 | 24.00 | -28.96 |
| Thailand (n = 142) | 92 | 14.00 | -49.50 |
| Thailand (n = 16 807) | 93 | 14.00 | -49.49 |
| Turkey (n = 4856) | 94 | 39.85 | -117.15 |
| UAE (n = 298) | 95 | 24.00 | -96.99 |
| UK (n = 1043) | 96 | 51.50 | -150.12 |
| UK 1000G (n = 96) | 97 | 51.60 | -150.15 |
| Vietnam (n = 170) | 98 | 21.02 | -44.17 |
| ^1^ Longitude modified as explained in Materials and Methods. | | | |

| **Supplementary Table 5. Geographic coordinates used for the *Barrier* analyses in East Asia and the Pacific (Figure 8c).** | | | |
| --- | --- | --- | --- |
| **Population** | **Code** | **Latitude** | **Long_mod_^1^** |
| Australia Cape York Peninsula Aborigine (n = 103) | 1 | -10.71 | -7.56 |
| Australia Kimberly Aborigine (n = 41) | 2 | -17.31 | -26.36 |
| Australia Yuendumu Aborigine (n = 191) | 3 | -23.69 | -16.12 |
| China (n = 1282) | 4 | 39.97 | -33.60 |
| China Han (n = 264) | 5 | 23.1 | -36.75 |
| China Han 1000G (n = 100) | 6 | 30.57 | -35.73 |
| China Beijing Shijiazhuang Tianjian Han (n = 618) | 7 | 39.9 | -33.59 |
| China Guangxi Region Maonan (n = 108) | 8 | 23.8 | -41.02 |
| China Jiangsu Han (n = 3238) | 9 | 21.95 | -49.55 |
| China Lisu (n = 111) | 10 | 24.5 | -48.50 |
| China Wuhan (n=121) | 11 | 30.75 | -35.75 |
| China Yunnan Bulang (n = 116) | 12 | 32.9 | -30.20 |
| Hong Kong (N=7595) | 13 | 22.25 | -35.83 |
| India Andhra Pradesh Golla (n = 111) | 14 | 17.08 | -71.92 |
| India Mumbai Maratha (n = 91) | 15 | 18.97 | -77.18 |
| India North pop 2 (n = 72) | 16 | 31.67 | -74.42 |
| Indonesia Java Western (n = 236) | 17 | -7.09 | -42.34 |
| Indonesia Javanese (n = 201) | 18 | -6.13 | -43.83 |
| Japan (n = 371) | 19 | 35.68 | -10.31 |
| Japan Ainu (n = 50) | 20 | 43.06 | -8.66 |
| Japan Tokio 1000G (n = 181) | 21 | 35.69 | -10.30 |
| Malaysia (n = 1445) | 22 | 3.13 | -48.30 |
| Malaysia Jelebu Temuan (n = 25) | 23 | 2.93 | -47.92 |
| Malaysia Kedah Baling Kensiu (n = 25) | 24 | 6.11 | -49.64 |
| Malaysia Perak Grik Jehai (n = 25) | 25 | 4.59 | -48.91 |
| Malaysia Sarawak Bau Bidayuh (n = 25) | 26 | 1.55 | -39.65 |
| Mongolia (n = 85) | 27 | 49.61 | -48.00 |
| Mongolia Khalkha | 28 | 47.916667 | -43.12 |
| Mongolia Oold | 29 | 48 | -54.00 |
| Myanmar Kayin (n = 44) | 30 | 17 | -52.25 |
| Myanmar Bamar (n = 46) | 31 | 19.75 | -52.20 |
| New Caledonia (n = 65) | 32 | -22.25 | 16.45 |
| New Caledonia 2 (n = 54) | 33 | -22.28 | 16.44 |
| New Zealand Maori (n = 46) | 34 | -21.22 | 50.23 |
| New Zealand Polynesian (n = 46) | 35 | -42 | 34.75 |
| Papua New Guinea East New Britain Rabaul (n = 60) | 36 | -4.19 | 2.16 |
| Highlanders from Papua New Guinea (n = 92) | 37 | -4.3 | 2.30 |
| Philippines Ivatan (n = 50) | 38 | 20.44 | -28.03 |
| Filipino from Luzon Island, Philippines (n=94) | 39 | 16.56 | -28.74 |
| Rapanui (n=49) | 40 | -27.11 | 100.65 |
| Russia Buryats | 41 | 54.266667 | -31.73 |
| Russia Tuva (n = 190) | 42 | 51.88 | -54.38 |
| Russia Tuva pop 2  (n = 169) | 43 | 51.89 | -54.37 |
| Samoa (n_CI_ = 51; n_CII_ = 50) | 44 | -14.3 | 39.30 |
| Singapore Riau Malay (n = 132) | 45 | 1.35 | -46.14 |
| South Korea (n = 485) | 46 | 37.56 | -23.03 |
| South Korea (n = 4128) | 47 | 37.66 | -22.92 |
| Taiwan Ami (n = 98) | 48 | 23 | -28.99 |
| Taiwan Bunun (n = 101) | 49 | 23.77 | -28.95 |
| Taiwan Tzu Chi (n = 710) | 50 | 23.77 | -28.97 |
| Taiwan Hakka (n = 55) | 51 | 25.08 | -28.92 |
| ^1^ Longitude modified as explained in Materials and Methods. | | | |

| **Supplementary Table 6. Geographic coordinates used for the *Barrier* analyses in the Americas (Figure 8d).** | | | |
| --- | --- | --- | --- |
| **Population** | **Code** | **Latitude** | **Long_mod_^1^** |
| Argentina Gran Chaco Eastern Toba (n = 135) | 1 | -26.19 | 151.82 |
| Argentina Gran Chaco Mataco Wichi (n = 49) | 2 | -22.52 | 146.19 |
| Argentina Gran Chaco Western Toba Pilaga (n = 19) | 3 | -31.61 | 149.31 |
| Argentina Rosario Toba (n = 86) | 4 | -29.26 | 149.83 |
| Bering (n = 85) | 5 | 55.00 | 16.27 |
| Aleuts from Bering strait 2 (n=104) | 6 | 55.10 | 16.28 |
| Bolivia Aymara (n = 102) | 7 | -16.00 | 141.40 |
| Bolivia Quechua (n = 80) | 8 | -16.48 | 141.16 |
| Brazil Ivaí Kaingang (n = 127) | 9 | -24.66 | 158.39 |
| Brazil Río das cobras Guaraní (n = 98) | 10 | -15.56 | 153.94 |
| Brazil Río das cobras Kaingang (n = 113) | 11 | -29.76 | 156.84 |
| Guarani-Kaiowá from Mato Grosso do Sul, Brazil (n=144) | 12 | -12.68 | 153.08 |
| Guarani-Ñandeva from Mato Grosso do Sul, Brazil (n=53) | 13 | -12.78 | 153.07 |
| Brazil Terena (n = 60) | 14 | -20.00 | 155.22 |
| Canada Chipewyan (n=25) | 15 | 59.60 | 102.67 |
| Canada Cree (n=18) | 16 | 50.33 | 107.50 |
| Canada Ojibwa (n=16) | 17 | 46.00 | 129.00 |
| Chile Huiliche (n=20) | 18 | -41.00 | 137.00 |
| Chile Mapuche (n = 104) | 19 | -37.80 | 136.61 |
| Colombia Inga (n=16) | 20 | 1.00 | 133.00 |
| Colombia Arara (n=17) | 21 | -4.00 | 140.00 |
| Colombia Tarapaca (n=19 | 22 | -4.10 | 139.90 |
| Colombia Waunana (n=20 | 23 | 5.00 | 138.00 |
| Colombia Wayu (n = 48) | 24 | 11.35 | 137.48 |
| Costa Rica Cabécar (n=19) | 25 | 10.00 | 126.00 |
| Costa Rica Ngäbe (n=18) | 26 | 9.00 | 128.00 |
| Guatemala Maya (n = 132) | 27 | 14.85 | 118.47 |
| Mexico Chiapas Lacandon Mayans (n=218) | 28 | 16.90 | 117.92 |
| Mexico Mayos (n = 60) | 29 | 26.41 | 101.39 |
| Mexico Mazatecan (n = 89) | 30 | 18.14 | 113.14 |
| Mexico Mixe (n = 55) | 31 | 15.88 | 114.15 |
| Mexico Mixtec (n = 103) | 32 | 17.01 | 113.27 |
| Mexico Nahuas (n = 85) | 33 | 19.03 | 110.90 |
| Mexico Tarahumara (n = 44) | 34 | 26.82 | 102.94 |
| Mexico Seri (n = 34) | 35 | 29.50 | 97.61 |
| Mexico Teenek (n = 55) | 36 | 22.15 | 109.02 |
| Mexico Teenek 2 (n=55) | 37 | 21.72 | 111.42 |
| Mexico Zapotec (n = 90) | 38 | 17.07 | 113.22 |
| Paraguay Guarani (n = 40) | 39 | -25.26 | 153.98 |
| Peru Lama (n = 83) | 40 | -6.42 | 133.47 |
| Peru Titikaka Lake Uro (n = 105) | 41 | -15.23 | 139.95 |
| USA Gila River (n = 492) | 42 | 33.07 | 96.58 |
| US Navajo (n=43) | 43 | 35.09 | 102.89 |
| USA Gila River Pima (n=3000) | 44 | 33.07 | 98.28 |
| USA Sioux (n = 302) | 45 | 43.91 | 107.26 |
| US Yupik (n = 252) | 46 | 60.79 | 45.20 |
| ^1^ Longitude modified as explained in Materials and Methods. | | | |

# Supplementary Figures


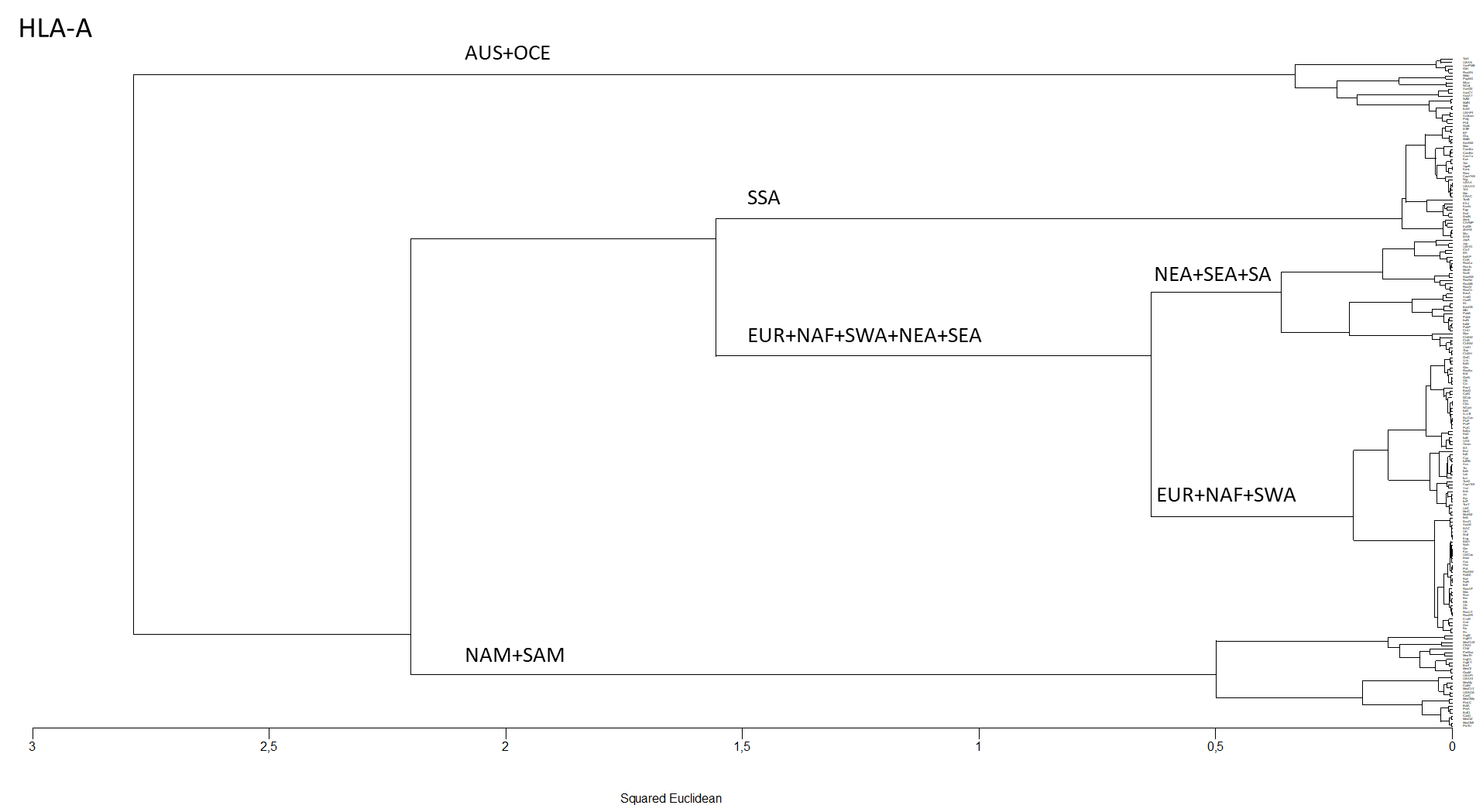


**Supplementary Figure 1.** Clustering based on HLA-A first-field frequencies. Dendrogram generated with minimum variance clustering analysis of squared Euclidean distances calculated using 20 HLA-A allele group frequencies in 200 worldwide populations. Main branches are labelled according to the predominant continental origin of populations forming its clusters. AUS, Australia; EUR, Europe; NAF, North Africa; NAM, North America; NEA, Northeast Asia; OCE, Oceania; SA, South Asia; SAM, South America; SEA, Southeast Asia; SWA, Southwest Asia; SSA, Sub-Saharan Africa.


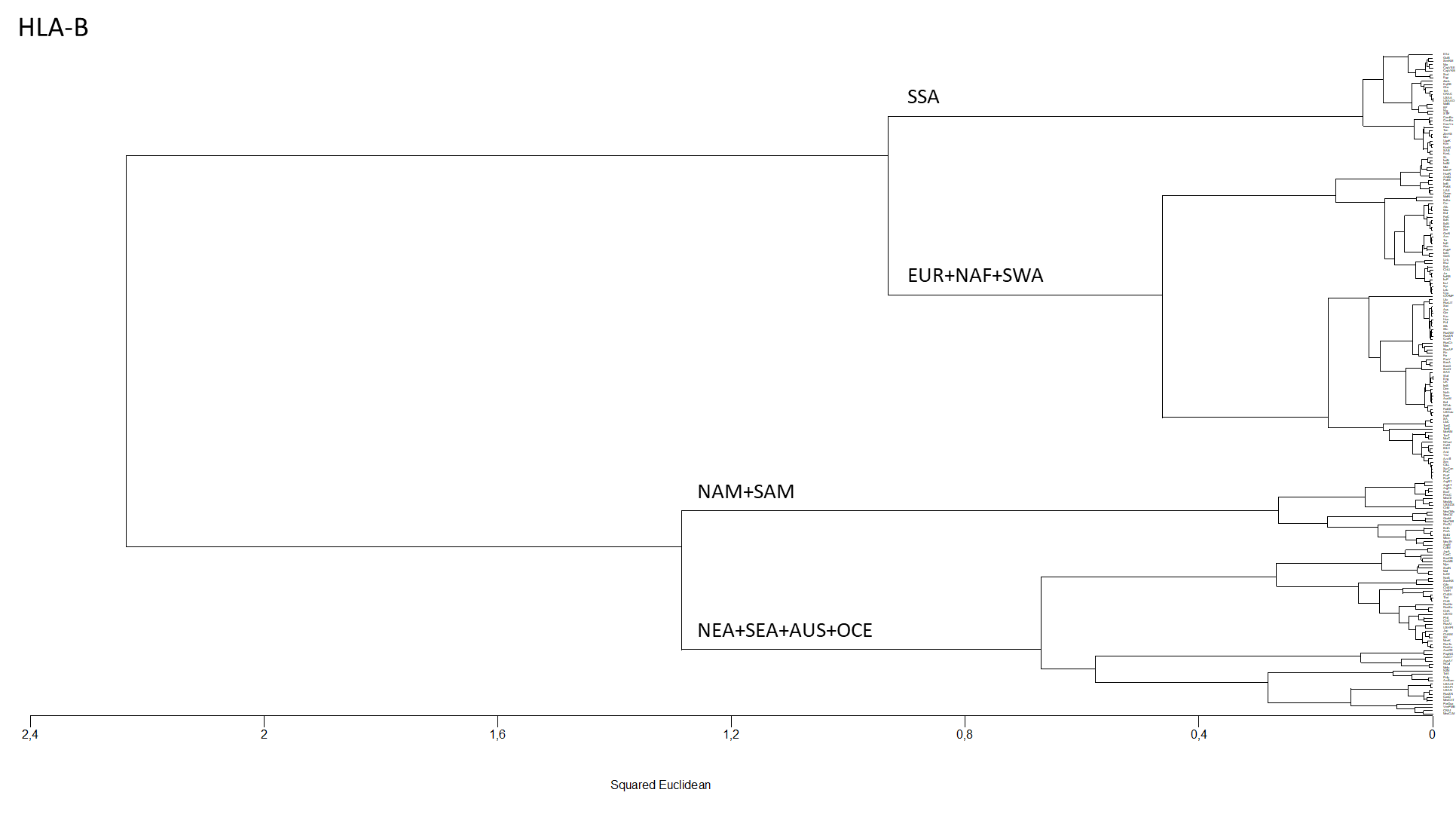


**Supplementary Figure 2.** Clustering based on HLA-B first-field frequencies. Dendrogram generated with minimum variance clustering analysis of squared Euclidean distances calculated using 30 HLA-B allele group frequencies in 200 worldwide populations. Main branches are labelled according to the predominant continental origin of populations forming its clusters. AUS, Australia; EUR, Europe; NAF, North Africa; NAM, North America; NEA, Northeast Asia; OCE, Oceania; SAM, South America; SEA, Southeast Asia; SWA, Southwest Asia; SSA, Sub-Saharan Africa.


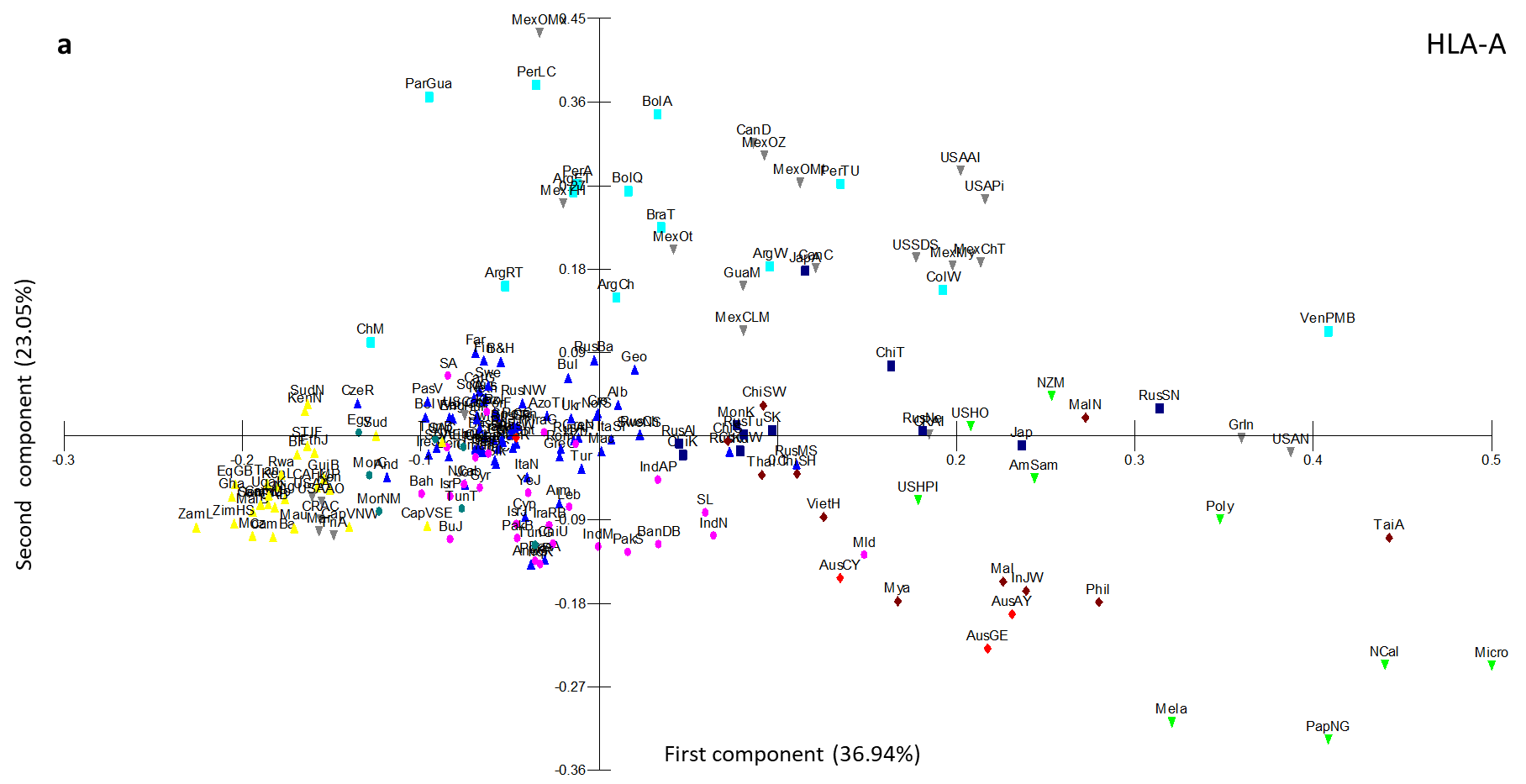


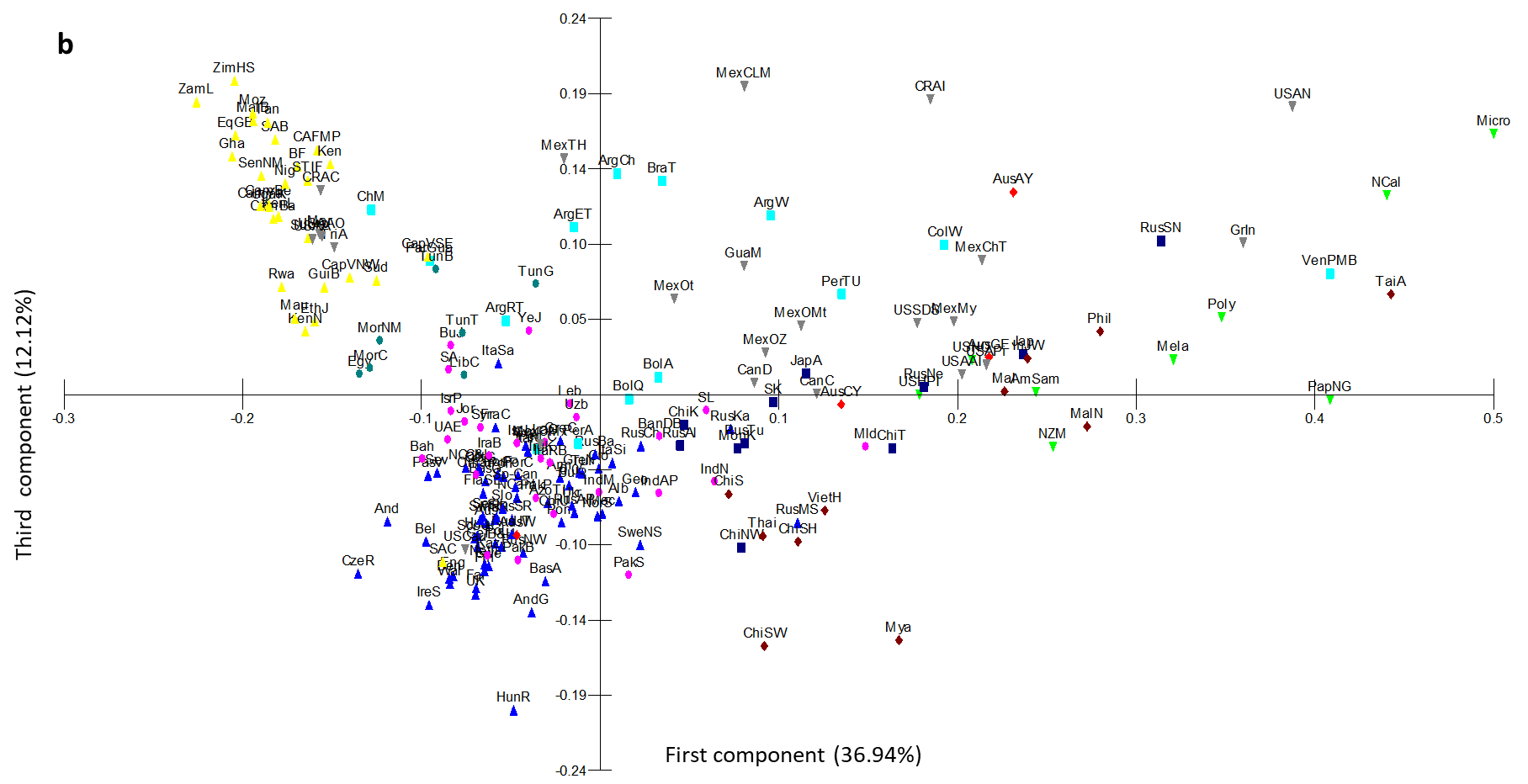


Second component (18.21%)

**
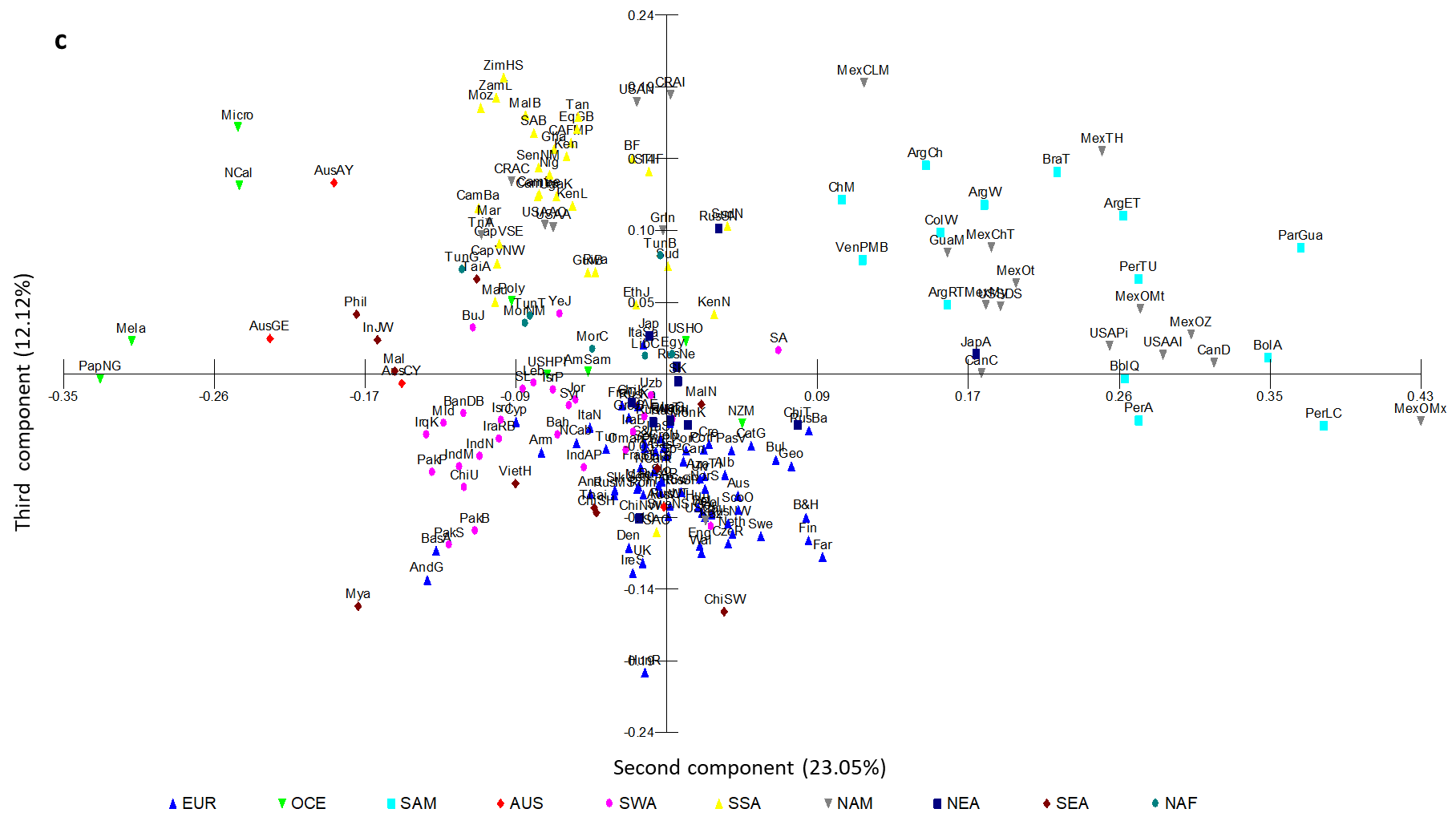
**

**Supplementary Figure 3.** PCoA scatterplots based on HLA-A first-field frequencies. a-c. Population scatterplots for the PCoA case scores based on Euclidean distances calculated on 20 HLA-A allele group frequencies in 200 worldwide populations are plotted for the first (36.94% of variation), second (23.05%), and third (12.12%) components. Population samples are depicted according to their geographic continental classification. AUS, Australia; EUR, Europe; NAF, North Africa; NAM, North America; NEA, Northeast Asia; OCE, Oceania; SAM, South America; SEA, Southeast Asia; SWA, Southwest Asia; SSA, Sub-Saharan Africa.


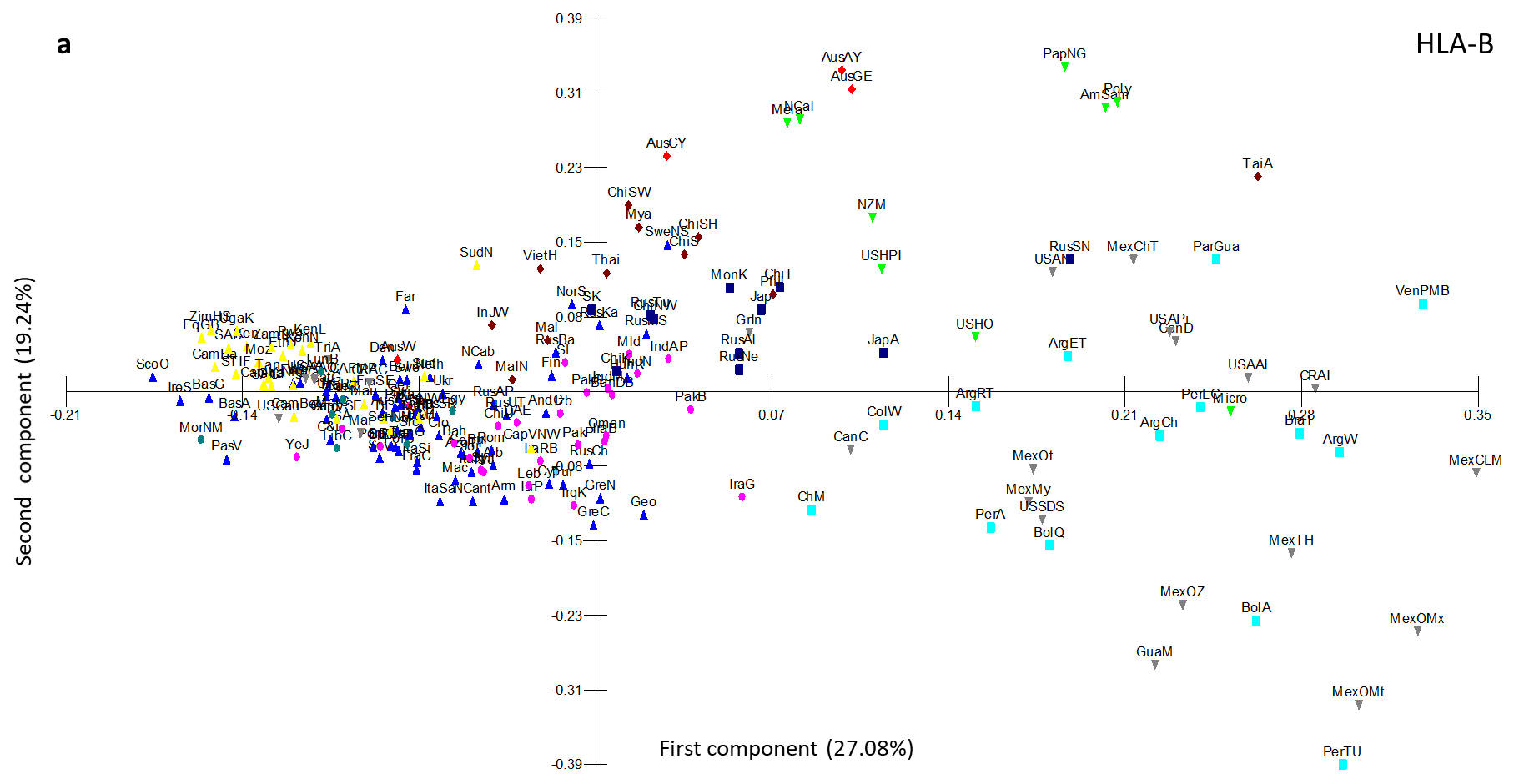


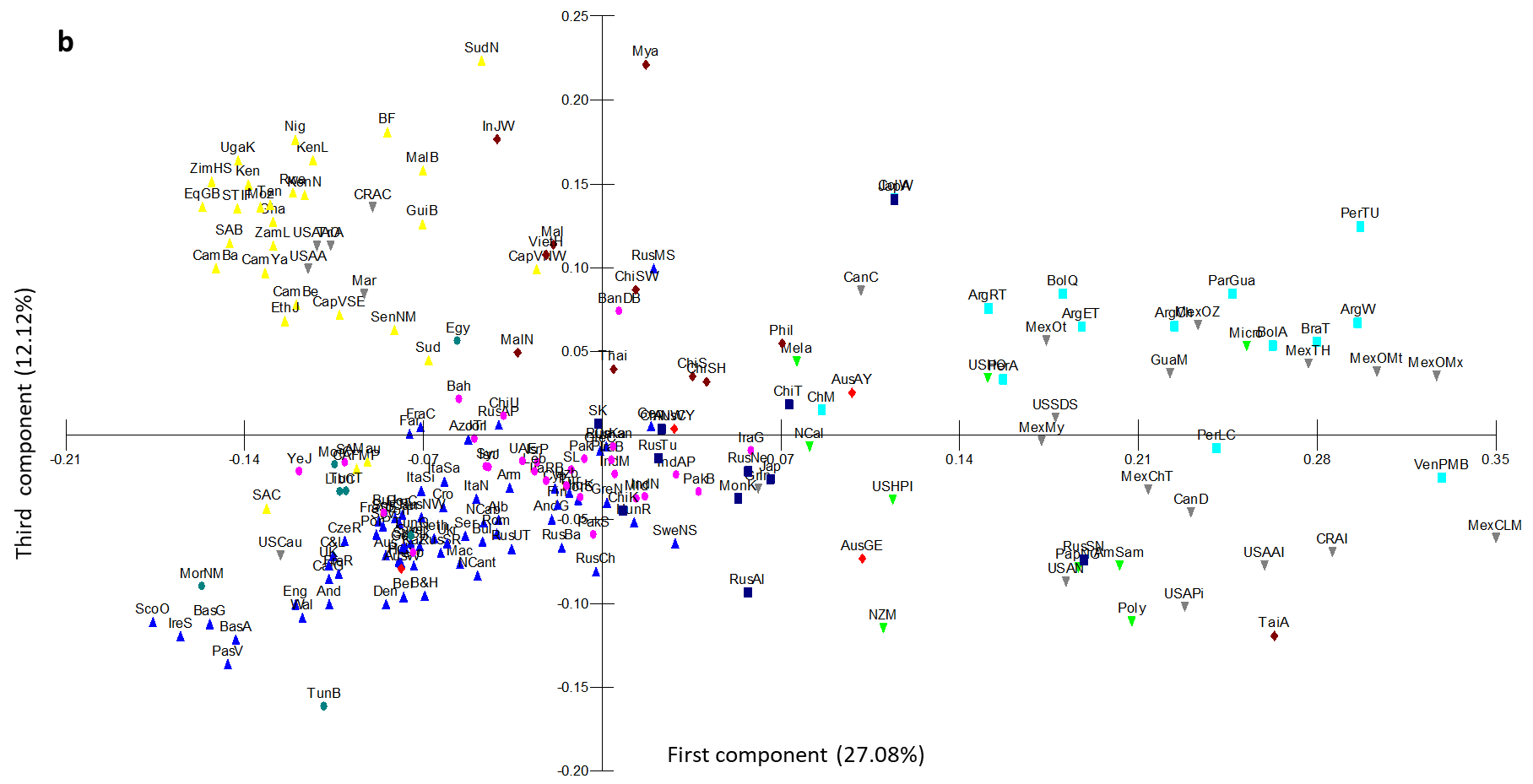


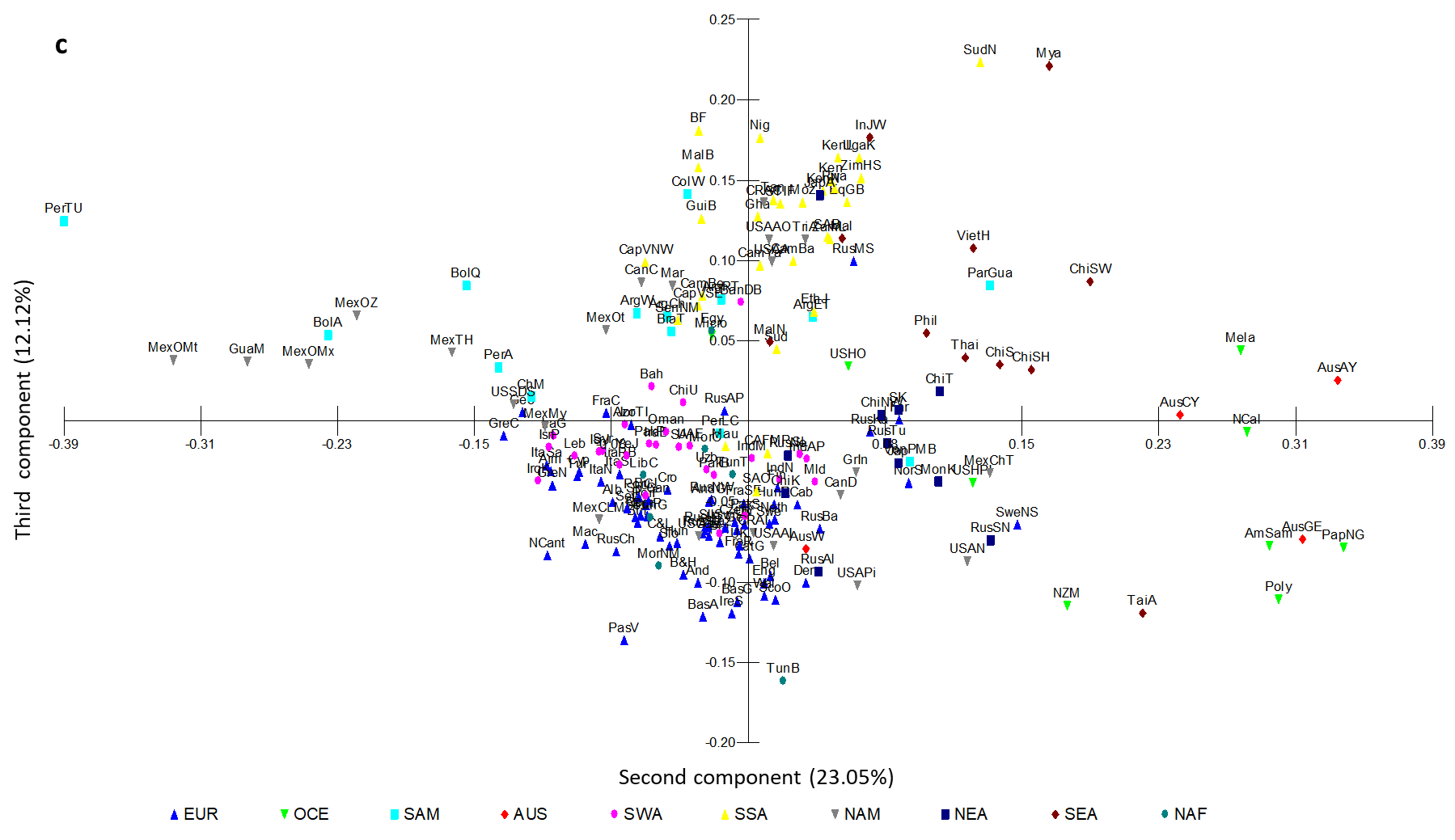


**Supplementary Figure 4.** PCoA scatterplots based on HLA-B first-field frequencies. a-c. Population scatterplots for the PCoA case scores based on Euclidean distances calculated on 30 HLA-B allele group frequencies in 200 worldwide populations are plotted for the first (27.08% of variation), second (19.24%), and third (11.26%) components. Population samples are depicted according to their geographic continental classification. AUS, Australia; EUR, Europe; NAF, North Africa; NAM, North America; NEA, Northeast Asia; OCE, Oceania; SAM, South America; SEA, Southeast Asia; SWA, Southwest Asia; SSA, Sub-Saharan Africa.


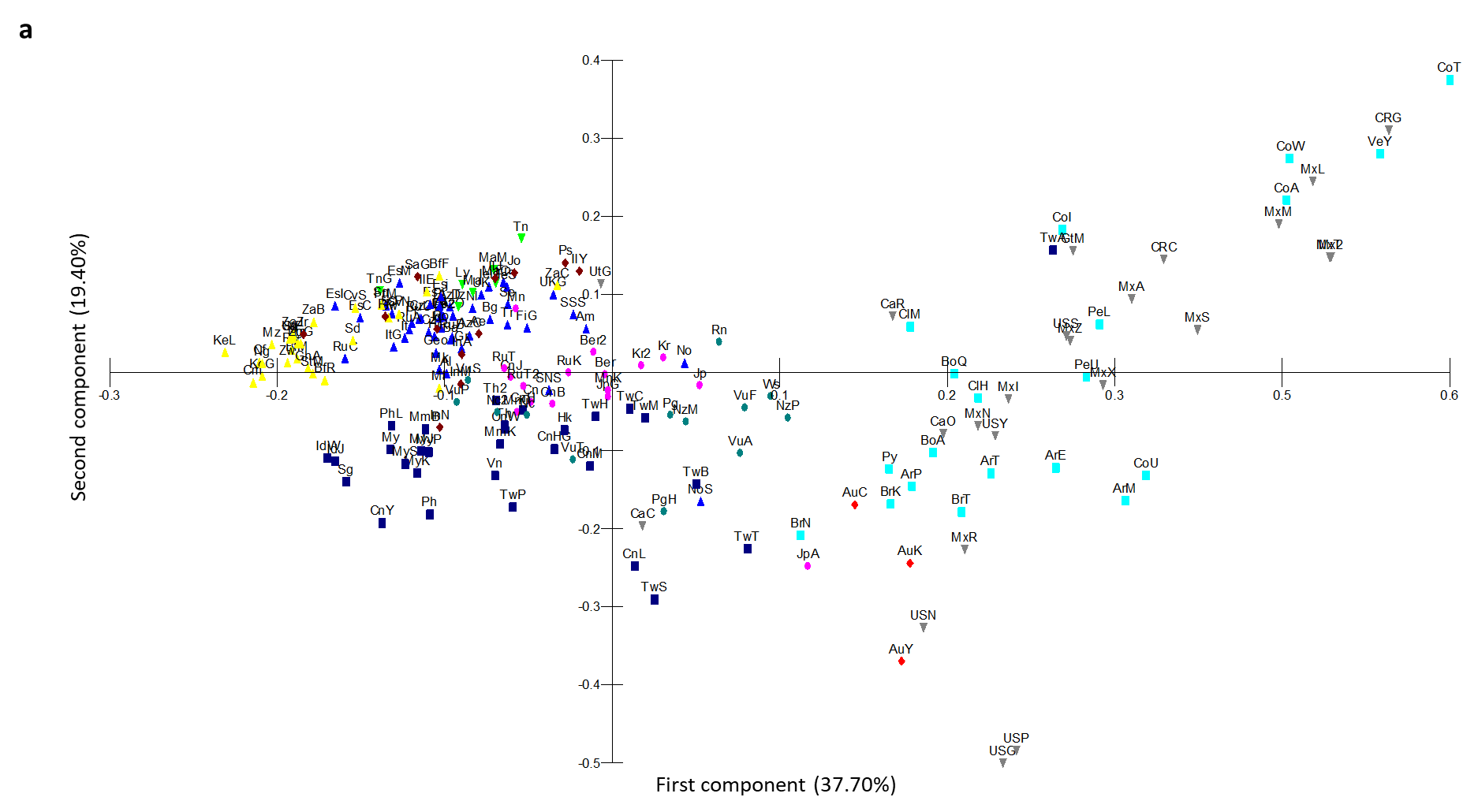


**
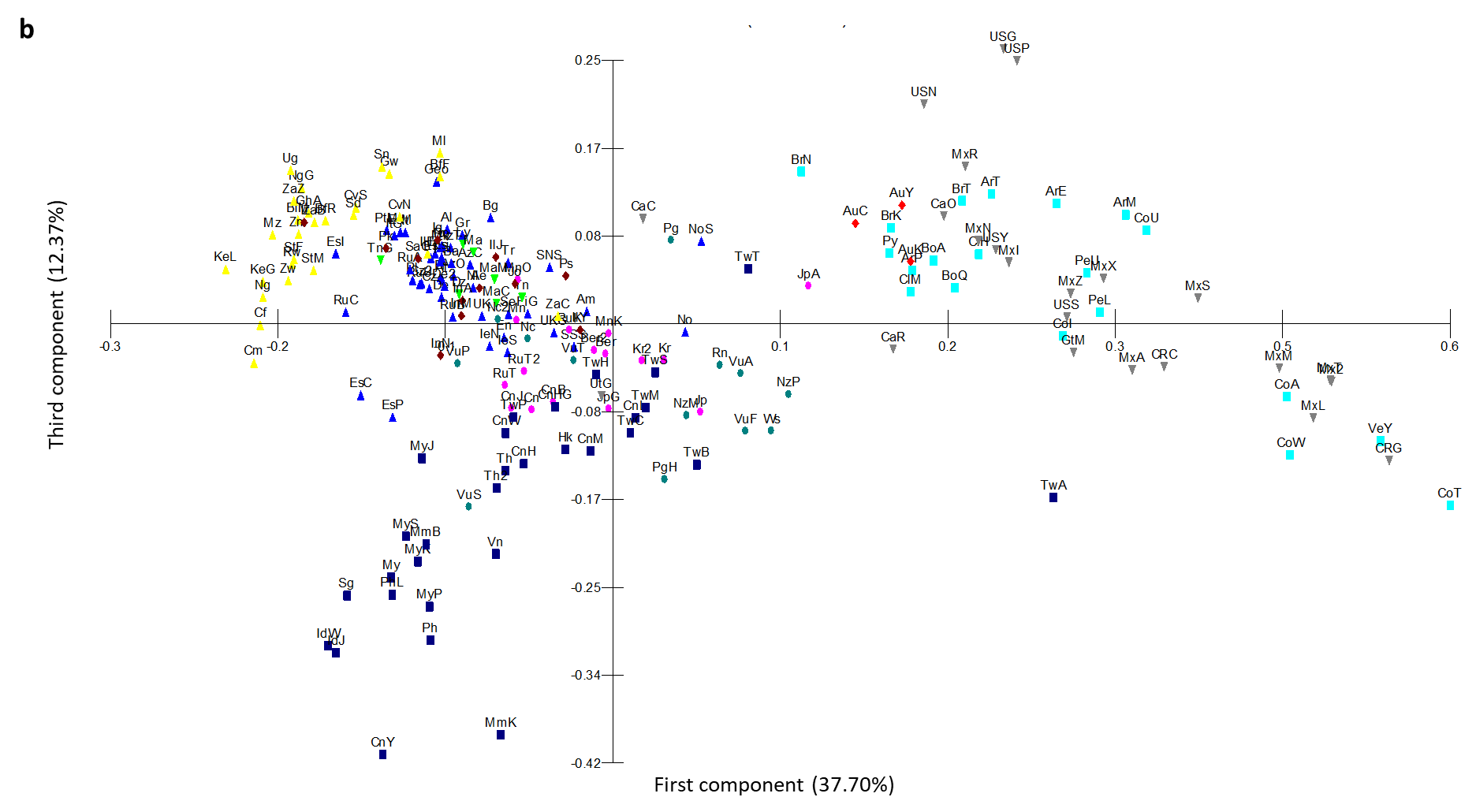
**

**
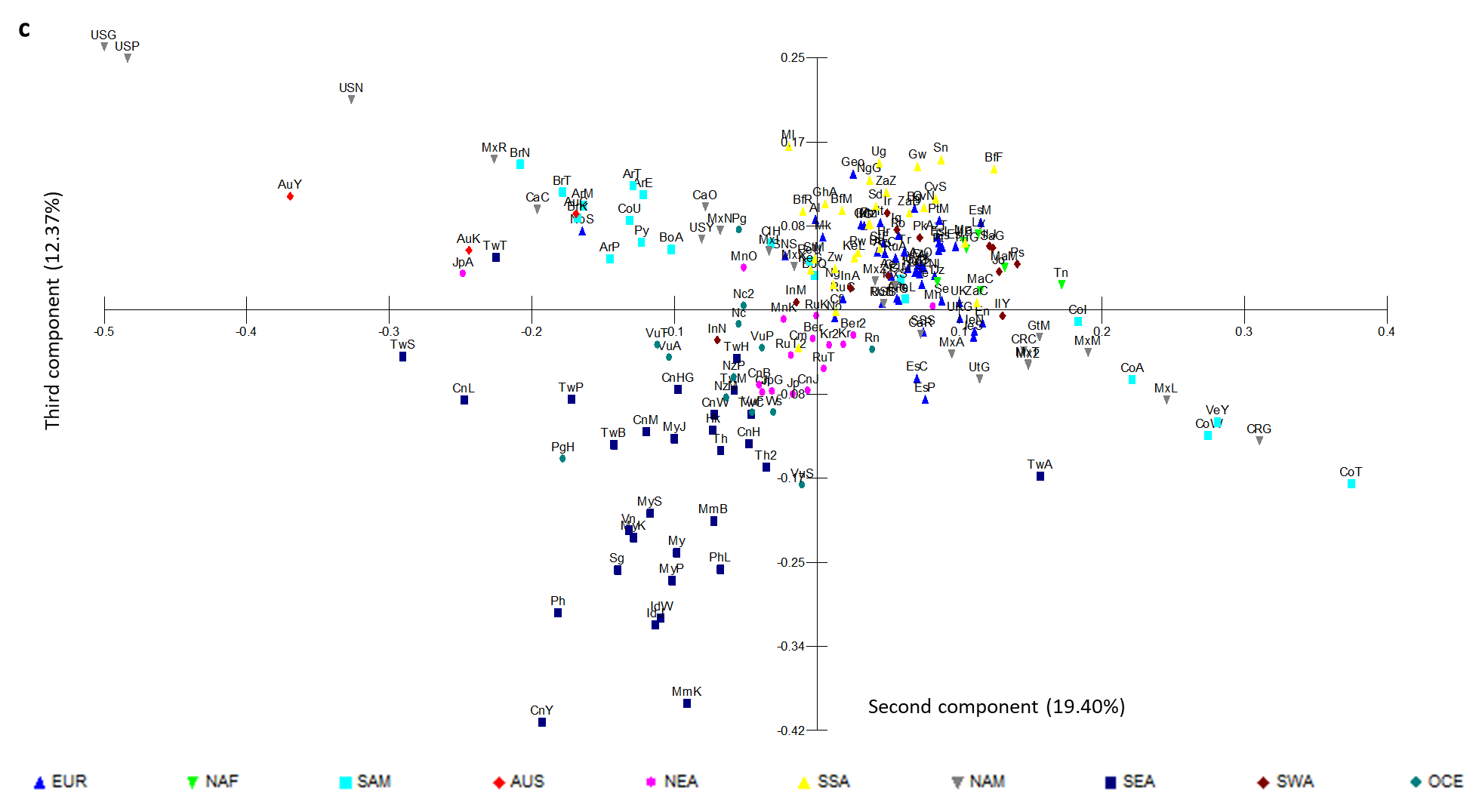
**

**Supplementary Figure 5.** **PCoA scatterplots based on HLA-DRB1 first-field frequencies**. a-c. Population scatterplots for the PCoA case scores based on Euclidean distances calculated on 13 HLA-DRB1 allele group frequencies in 197 worldwide populations are plotted for the first (37.70% of variation), second (19.40%), and third (12.37%) components. Population samples are depicted according to their geographic continental classification. AUS, Australia; EUR, Europe; NAF, North Africa; NAM, North America; NEA, Northeast Asia; OCE, Oceania; SAM, South America; SEA, Southeast Asia; SWA, Southwest Asia; SSA, Sub-Saharan Africa.

**
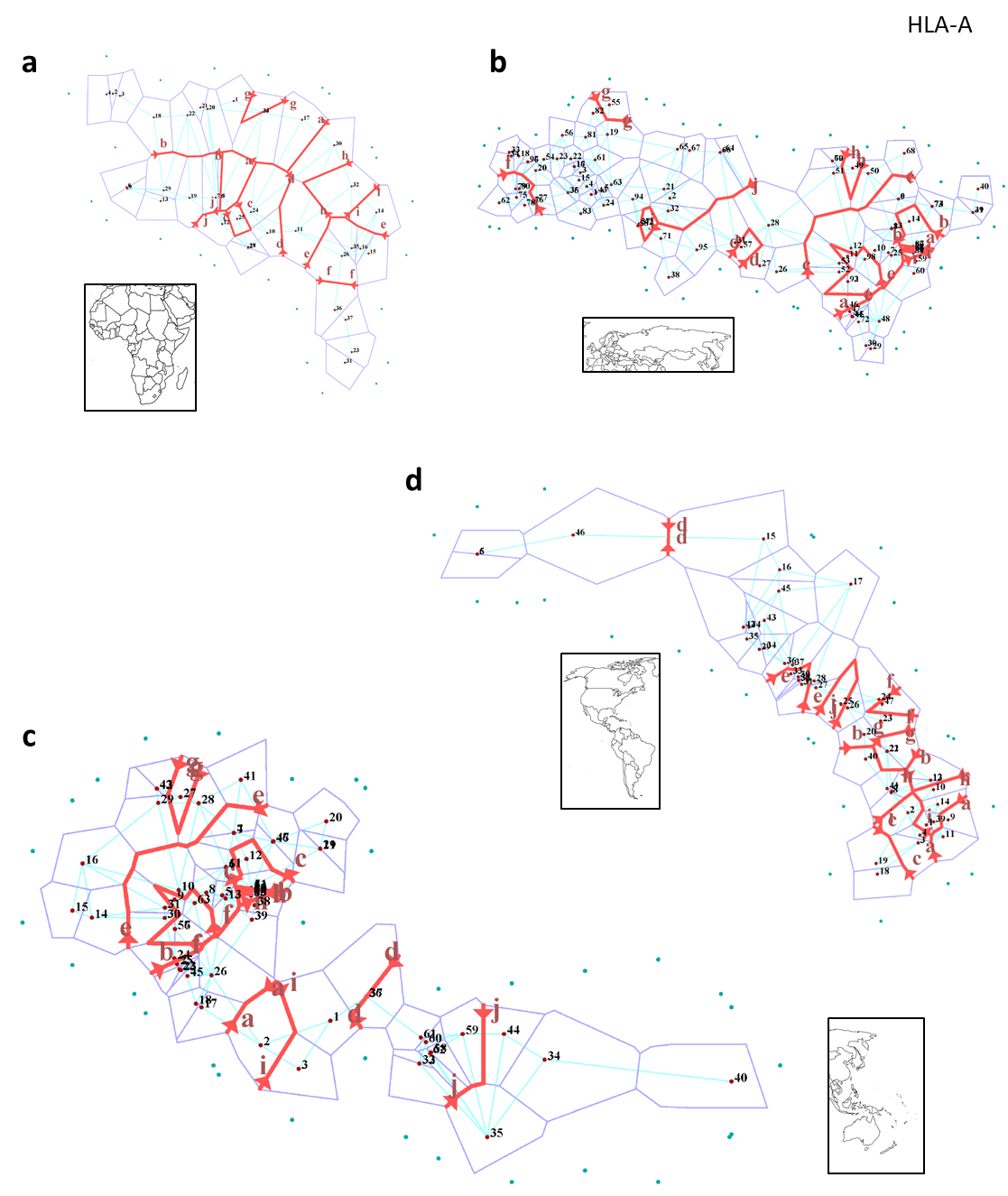
**

**Supplementary Figure 6.** *Barrier* analysis based on HLA-A frequencies. Plots show genetic discontinuities (red lines) based on the analysis of HLA-A first-field frequencies for populations located in a. Africa, b. Eurasia, c. East Asia and the Pacific, and d. the Americas. Numbers correspond to the populations included in the *Barrier* analyses (Supplementary Tables 3-6).

**
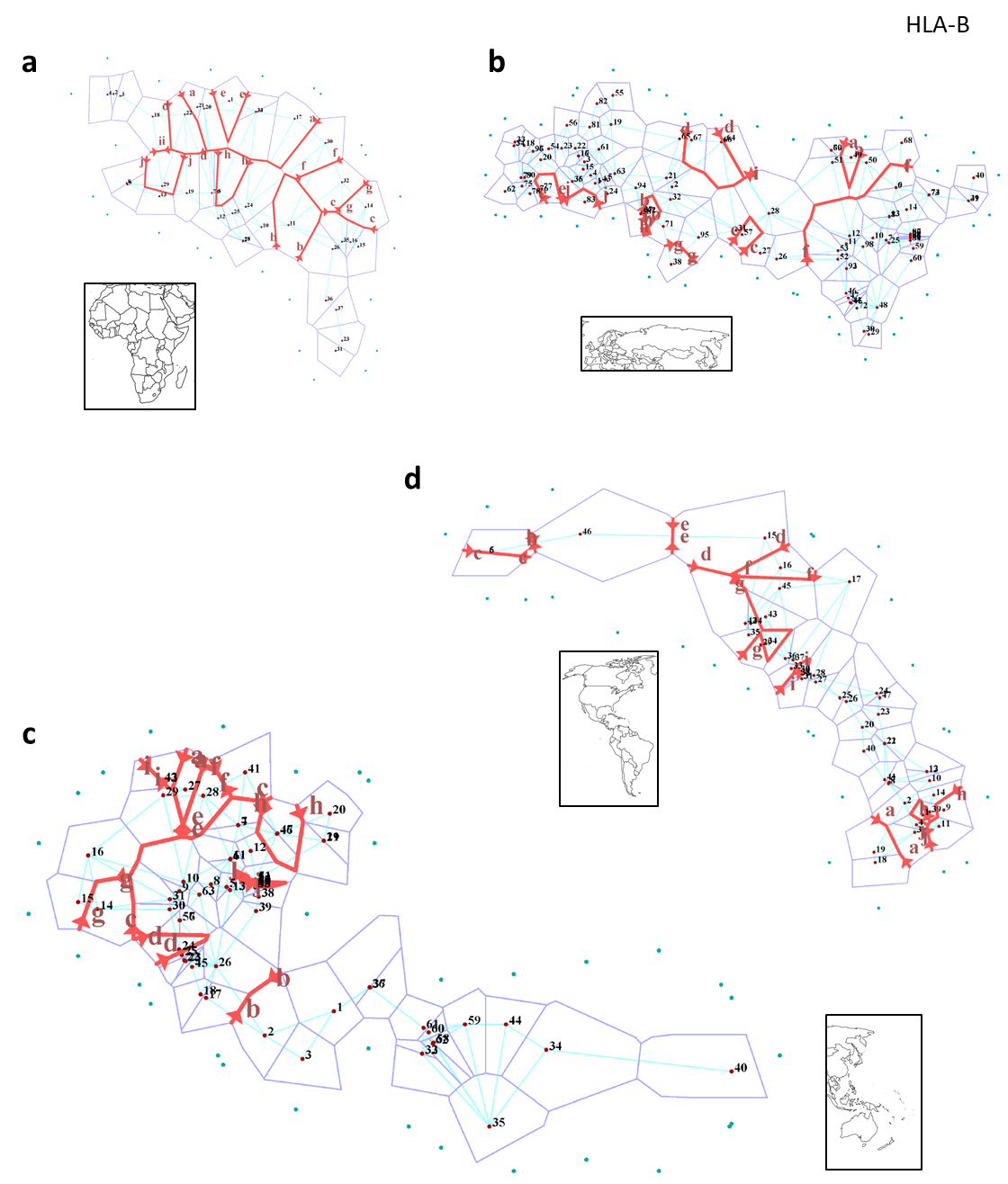
**

**Supplementary Figure 7.** *Barrier* analysis based on HLA-B frequencies. Plots show genetic discontinuities (red lines) based on the analysis of HLA-B first-field frequencies for populations located in a. Africa, b. Eurasia, c. East Asia and the Pacific, and d. the Americas. Numbers correspond to the populations included in the *Barrier* analyses (Supplementary Tables 3-6).

**
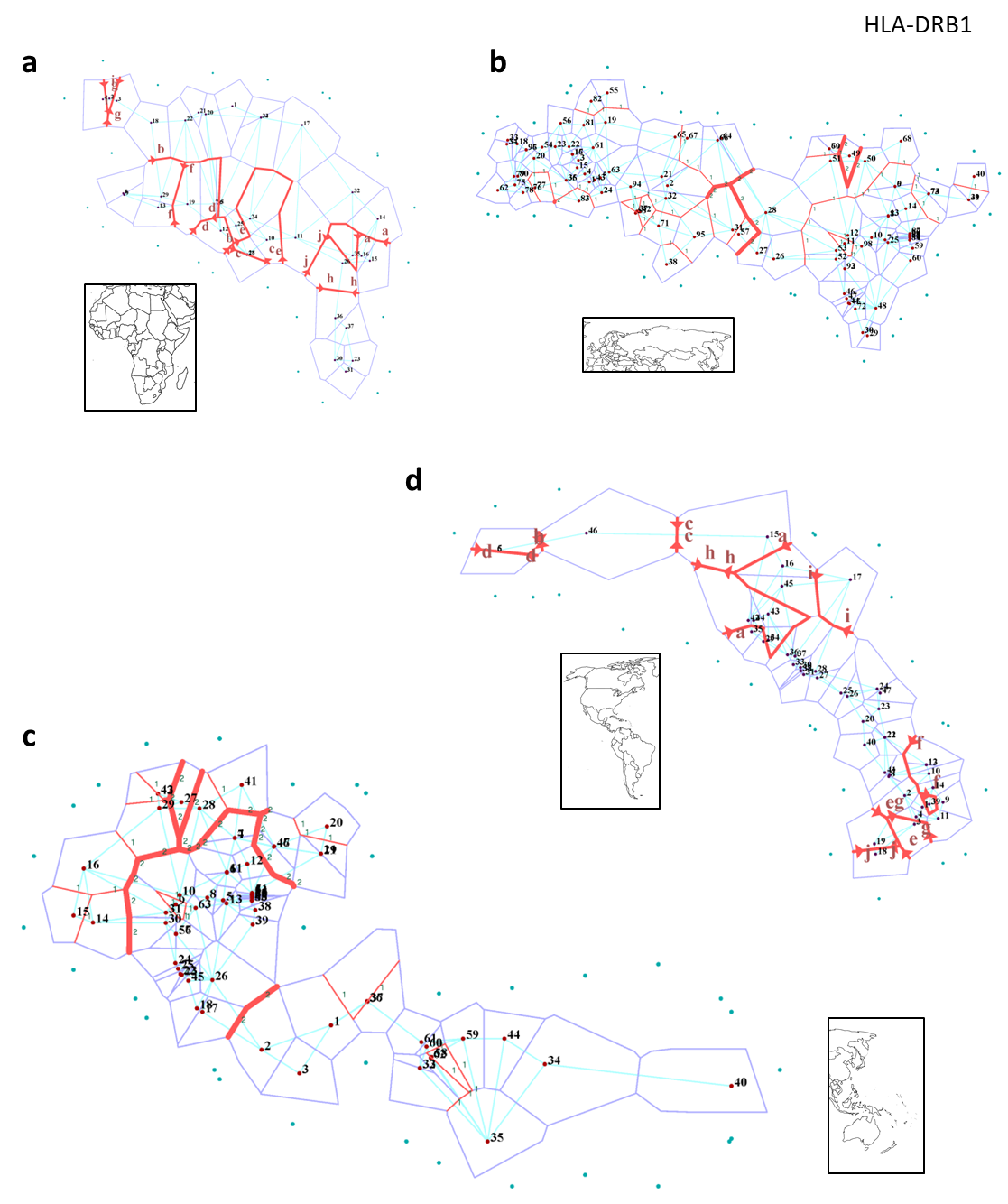
**

**Supplementary Figure 8.** *Barrier* analysis based on HLA-DRB1 frequencies. Plots show genetic discontinuities based (red lines) on the analysis of HLA-DRB1 first-field frequencies for populations located in a. Africa, b. Eurasia, c. East Asia and the Pacific, and d. the Americas. Numbers correspond to the populations included in the *Barrier* analyses (Supplementary Tables 3-6).

**
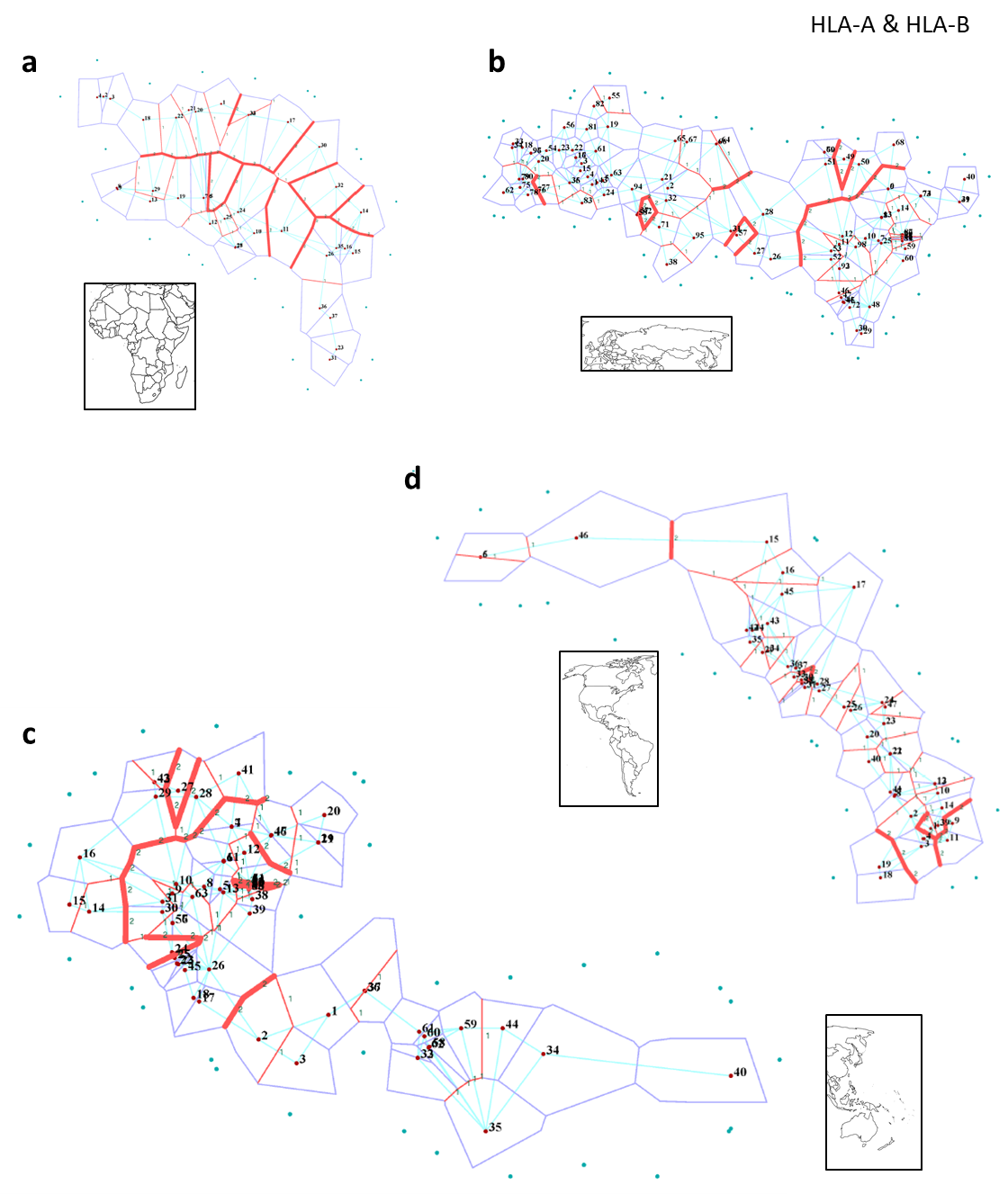
**

**Supplementary Figure 9.** *Barrier* analysis based on HLA-A & HLA-B frequencies. Plots show genetic discontinuities based on the joint analysis of HLA-A and HLA-B first-field frequencies for populations located in a. Africa, b. Eurasia, c. East Asia and the Pacific, and d. the Americas. Numbers correspond to the populations included in the *Barrier* analyses (Supplementary Tables 3-6). Statistically significant genetic barriers are highlighted as thick red lines.

**References for HLA-A and HLA-B**

Alcoceba, M., Marin, L., Balanzategui, A., Sarasquete, M. E., Chillon, M. C., Martin-Jimenez, P., Puig, N., Santamaria, C., Corral, R., Garcia-Sanz, R., San Miguel, J. F. & Gonzalez, M. (2011) Frequency of HLA-A, -B and -DRB1 specificities and haplotypic associations in the population of Castilla y Leon (northwest-central Spain). *Tissue Antigens*, 78(4), 249-55.

Ali, M. E., Ahmed, M. U., Alam, S. & Rahman, M. H. (2008) HLA-A, -B and -DRB1 allele frequencies in the Bangladeshi population. *Tissue Antigens*, 72(2), 115-9.

Arnaiz-Villena, A., Dimitroski, K., Pacho, A., Moscoso, J., Gomez-Casado, E., Silvera-Redondo, C., Varela, P., Blagoevska, M., Zdravkovska, V. & Martinez-Laso, J. (2001) HLA genes in Macedonians and the sub-Saharan origin of the Greeks. *Tissue Antigens*, 57(2), 118-27.

Arnaiz-Villena, A., Gonzalez-Alcos, V., Serrano-Vela, J. I., Reguera, R., Barbolla, L., Parga-Lozano, C., Gomez-Prieto, P., Abd-El-Fatah-Khalil, S. & Moscoso, J. (2009) HLA genes in Uros from Titikaka Lake, Peru: origin and relationship with other Amerindians and worldwide populations. *Int J Immunogenet*, 36(3), 159-67.

Arnaiz-Villena, A., Iliakis, P., Gonzalez-Hevilla, M., Longas, J., Gomez-Casado, E., Sfyridaki, K., Trapaga, J., Silvera-Redondo, C., Matsouka, C. & Martinez-Laso, J. (1999) The origin of Cretan populations as determined by characterization of HLA alleles. *Tissue Antigens*, 53(3), 213-26.

Arnaiz-Villena, A., Lopez-Nares, A., Callado, A., A, H. S., Rashidi, F., Palacio-Gruber, J. & Juarez, I. (2019) Study of HLA genes in Russia Bering Island Aleuts. *Hum Immunol*, 80(9), 631-632.

Arnaiz-Villena, A., Martinez-Laso, J., Moscoso, J., Livshits, G., Zamora, J., Gomez-Casado, E., Silvera-Redondo, C., Melvin, K. & Crawford, M. H. (2003) HLA genes in the Chuvashian population from European Russia: admixture of Central European and Mediterranean populations. *Hum Biol*, 75(3), 375-92.

Arnaiz-Villena, A., Moscoso, J., Granados, J., Serrano-Vela, J. I., de la Pena, A., Reguera, R., Ferri, A., Seclen, E., Izaguirre, R., Perez-Hernandez, N. & Vargas-Alarcon, G. (2007) HLA Genes in Mayos Population from Northeast Mexico. *Curr Genomics*, 8(7), 466-75.

Arnaiz-Villena, A., Siles, N., Moscoso, J., Zamora, J., Serrano-Vela, J. I., Gomez-Casado, E., Castro, M. J. & Martinez-Laso, J. (2005) Origin of Aymaras from Bolivia and their relationship with other Amerindians according to HLA genes. *Tissue Antigens*, 65(4), 379-90.

Arrieta-Bolanos, E., Madrigal-Sanchez, J. J., Stein, J. E., Arrieta-Molina, G., Grant, S., Salazar-Sanchez, L., Madrigal, J. A., Marsh, S. G. E. & Shaw, B. E. (2019a) 4-Locus high-resolution HLA allele and haplotype frequencies in Costa Ricans from African-Caribbean descent. *Hum Immunol*, 80(7), 411-412.

Arrieta-Bolanos, E., Madrigal-Sanchez, J. J., Stein, J. E., Salazar-Sanchez, L., Madrigal, J. A., Marsh, S. G. E. & Shaw, B. E. (2019b) 4-Locus high-resolution HLA allele and haplotype frequencies in Amerindians from Costa Rica. *Hum Immunol*, 80(7), 409-410.

Assane, A. A., Fabricio-Silva, G. M., Cardoso-Oliveira, J., Mabunda, N. E., Sousa, A. M., Jani, I. V., Ferreira, O. C., Jr. & Porto, L. C. (2010) Human leukocyte antigen-A, -B, and -DRB1 allele and haplotype frequencies in the Mozambican population: a blood donor-based population study. *Human immunology*, 71(10), 1027-32.

Bannai, M., Ohashi, J., Harihara, S., Takahashi, Y., Juji, T., Omoto, K. & Tokunaga, K. (2000) Analysis of HLA genes and haplotypes in Ainu (from Hokkaido, northern Japan) supports the premise that they descent from Upper Paleolithic populations of East Asia. *Tissue Antigens*, 55(2), 128-39.

Barquera, R., Zuniga, J., Flores-Rivera, J., Corona, T., Penman, B. S., Hernandez-Zaragoza, D. I., Soler, M., Jonapa-Gomez, L., Mallempati, K. C., Yescas, P., Ochoa-Morales, A., Barsakis, K., Aguilar-Vazquez, J. A., Garcia-Lechuga, M., Mindrinos, M., Yunis, M., Jimenez-Alvarez, L., Mena-Hernandez, L., Ortega, E., Cruz-Lagunas, A., Tovar-Mendez, V. H., Granados, J., Fernandez-Vina, M. & Yunis, E. (2020) Diversity of HLA Class I and Class II blocks and conserved extended haplotypes in Lacandon Mayans. *Sci Rep*, 10(1), 3248.

Benitez, O., Busson, M., Charron, D. & Loiseau, P. (2011) HLA polymorphism in a Guarani-Indian population from Paraguay and its usefulness for the Hispano-Indian admixture study in Paraguay. *International journal of immunogenetics*, 38(1), 7-11.

Bera, O., Cesaire, R., Quelvennec, E., Quillivic, F., de Chavigny, V., Ribal, C. & Semana, G. (2001) HLA class I and class II allele and haplotype diversity in Martinicans. *Tissue Antigens*, 57(3), 200-7.

Bettencourt, B. F., Santos, M. R., Pereira, J., Amaro, B., Fialho, R., Meneses, R., Couto, A. R. & Bruges Armas, J. (2016) HLA-A, -B, -C, -DQA1, -DQB1, -DRB1, -E, -F and -G genotyping of 130 individuals from Terceira Island, Azores, Portugal. *Hum Immunol*, 77(6), 445-6.

Bonanno, C. T., Cigna, D., Danna, C., D'Anna, R. P., Di Sano, C., Matranga, D., Raffa, M., Impeduglia, A. & Salerno, A. (2007) HLA class I and class II polymorphism in three Sicilian populations. *Hum Biol*, 79(3), 339-54.

Boyton, R. J., Smith, J., Jones, M., Reynolds, C., Ozerovitch, L., Chaudhry, A., Wilson, R., Rose, M. & Altmann, D. M. (2008) Human leucocyte antigen class II association in idiopathic bronchiectasis, a disease of chronic lung infection, implicates a role for adaptive immunity. *Clin Exp Immunol*, 152(1), 95-101.

Bruges Armas, J., Destro-Bisol, G., Lopez-Vazquez, A., Couto, A. R., Spedini, G., Gonzalez, S., Battaggia, C., Peixoto, M. J., Martinez-Borra, J. & Lopez-Larrea, C. (2003) HLA class I variation in the West African Pygmies and their genetic relationship with other African populations. *Tissue Antigens*, 62(3), 233-42.

Buhler, S., Nunes, J. M., Nicoloso, G., Tiercy, J. M. & Sanchez-Mazas, A. (2012) The heterogeneous HLA genetic makeup of the Swiss population. *PLoS One*, 7(7), e41400.

Cao, K., Moormann, A. M., Lyke, K. E., Masaberg, C., Sumba, O. P., Doumbo, O. K., Koech, D., Lancaster, A., Nelson, M., Meyer, D., Single, R., Hartzman, R. J., Plowe, C. V., Kazura, J., Mann, D. L., Sztein, M. B., Thomson, G. & Fernandez-Vina, M. A. (2004) Differentiation between African populations is evidenced by the diversity of alleles and haplotypes of HLA class I loci. *Tissue Antigens*, 63(4), 293-325.

Chen, S., Hong, W., Shao, H., Fu, Y., Liu, X., Chen, D. & Xu, A. (2006) Allelic distribution of HLA class I genes in the Tibetan ethnic population of China. *Int J Immunogenet*, 33(6), 439-45.

Chhaya, S., Desai, S. & Saranath, D. (2010) HLA polymorphisms in Sindhi community in Mumbai, India. *Int J Immunogenet*, 37(5), 373-7.

Choukri, F., Chakib, A., Himmich, H., Raissi, H. & Caillat-Zucman, S. (2002) HLA class I polymorphism in a Moroccan population from Casablanca. *Eur J Immunogenet*, 29(3), 205-11.

Comas, D., Mateu, E., Calafell, F., Perez-Lezaun, A., Bosch, E., Martinez-Arias, R. & Bertranpetit, J. (1998) HLA class I and class II DNA typing and the origin of Basques. *Tissue Antigens*, 51(1), 30-40.

de Pablo, R., Beraun, Y., Nieto, A., Calzada, J. E., Rementeria, M. C., Sanz, L., Lopez-Nevot, M. A. & Martin, J. (2000) HLA class I and class II allele distribution in the Peruvian population. *Tissue Antigens*, 56(6), 507-14.

de Pablo, R., Garcia-Pacheco, J. S., Vilches, C. & Moereno, M. E. (1998) Bubi normal, in Gjertson, D. W. & Terasaki, P. I. (eds), *HLA 1998*. USA: American society for Histocompatibility and Immunogenetics.

Dhaliwal, J. S., Shahnaz, M., Too, C. L., Azrena, A., Maiselamah, L., Lee, Y. Y., Irda, Y. A. & Salawati, M. (2007) HLA-A, -B and -DR allele and haplotype frequencies in Malays. *Asian Pac J Allergy Immunol*, 25(1), 47-51.

Du, K. M., Ji, Y., Xie, J. H., Fu, M., Sun, Y., Jin, Y., Sun, J. L., Yang, J. H., Zhang, Z., Mao, Z., Liu, D. Z., Qian, K. C. & Zhao, T. M. (2007) HLA-A, -B, -DR haplotype frequencies from DNA typing data of 26,266 Chinese bone marrow donors. *Hum Immunol*, 68(10), 854-66.

Dunne, C., Crowley, J., Hagan, R., Rooney, G. & Lawlor, E. (2008) HLA-A, B, Cw, DRB1, DQB1 and DPB1 alleles and haplotypes in the genetically homogenous Irish population. *Int J Immunogenet*, 35(4-5), 295-302.

Edinur, H. A., Dunn, P. P., Hammond, L., Selwyn, C., Velickovic, Z. M., Lea, R. A. & Chambers, G. K. (2012) Using HLA loci to inform ancestry and health in Polynesian and Maori populations. *Tissue Antigens*, 80(6), 509-22.

Elbjeirami, W. M., Abdel-Rahman, F. & Hussein, A. A. (2013) Probability of finding an HLA-matched donor in immediate and extended families: the Jordanian experience. *Biol Blood Marrow Transplant*, 19(2), 221-6.

Ellis, J. M., Mack, S. J., Leke, R. F., Quakyi, I., Johnson, A. H. & Hurley, C. K. (2000) Diversity is demonstrated in class I HLA-A and HLA-B alleles in Cameroon, Africa: description of HLA-A*03012, *2612, *3006 and HLA-B*1403, *4016, *4703. *Tissue Antigens*, 56(4), 291-302.

Evseeva, I., Spurkland, A., Thorsby, E., Smerdel, A., Tranebjaerg, L., Boldyreva, M., Groudakova, E., Gouskova, I. & Alexeev, L. L. (2002) HLA profile of three ethnic groups living in the North-Western region of Russia. *Tissue Antigens*, 59(1), 38-43.

Farjadian, S., Naruse, T., Kawata, H., Ghaderi, A., Bahram, S. & Inoko, H. (2004) Molecular analysis of HLA allele frequencies and haplotypes in Baloch of Iran compared with related populations of Pakistan. *Tissue Antigens*, 64(5), 581-7.

Fernandez-Vina, M. A., Lazaro, A. M., Marcos, C. Y., Nulf, C., Raimondi, E., Haas, E. J. & Stastny, P. (1997) Dissimilar evolution of B-locus versus A-locus and class II loci of the HLA region in South American Indian tribes. *Tissue Antigens*, 50(3), 233-50.

Galgani, A., Mancino, G., Martinez-Labarga, C., Cicconi, R., Mattei, M., Amicosante, M., Bonanno, C. T., Di Sano, C., Gimil, G. S., Salerno, A., Colizzi, V. & Montesano, C. (2013) HLA-A, -B and -DRB1 allele frequencies in Cyrenaica population (Libya) and genetic relationships with other populations. *Hum Immunol*, 74(1), 52-9.

Gao, X., Lester, S., Boetcher, B. & McCluskey, J. (1997) Diversity of HLA genes in populations of Australia and the Pacific, *Proceedings of the Twelfth International Histocompatibility Workshop and Conference*. EDK.

Garcia-Ortiz, J. E., Sandoval-Ramirez, L., Rangel-Villalobos, H., Maldonado-Torres, H., Cox, S., Garcia-Sepulveda, C. A., Figuera, L. E., Marsh, S. G., Little, A. M., Madrigal, J. A., Moscoso, J., Arnaiz-Villena, A. & Arguello, J. R. (2006) High-resolution molecular characterization of the HLA class I and class II in the Tarahumara Amerindian population. *Tissue Antigens*, 68(2), 135-46.

Gomez-Casado, E., Martinez-Laso, J., Moscoso, J., Zamora, J., Martin-Villa, M., Perez-Blas, M., Lopez-Santalla, M., Lucas Gramajo, P., Silvera, C., Lowy, E. & Arnaiz-Villena, A. (2003) Origin of Mayans according to HLA genes and the uniqueness of Amerindians. *Tissue Antigens*, 61(6), 425-36.

Gonzalez-Galarza, F. F., McCabe, A., Santos, E., Jones, J., Takeshita, L., Ortega-Rivera, N. D., Cid-Pavon, G. M. D., Ramsbottom, K., Ghattaoraya, G., Alfirevic, A., Middleton, D. & Jones, A. R. (2020) Allele frequency net database (AFND) 2020 update: gold-standard data classification, open access genotype data and new query tools. *Nucleic Acids Res*, 48(D1), D783-D788.

Gragert, L., Madbouly, A., Freeman, J. & Maiers, M. (2013) Six-locus high resolution HLA haplotype frequencies derived from mixed-resolution DNA typing for the entire US donor registry. *Hum Immunol*, 74(10), 1313-20.

Grimaldi, M. C., Crouau-Roy, B., Amoros, J. P., Cambon-Thomsen, A., Carcassi, C., Orru, S., Viader, C. & Contu, L. (2001) West Mediterranean islands (Corsica, Balearic islands, Sardinia) and the Basque population: contribution of HLA class I molecular markers to their evolutionary history. *Tissue Antigens*, 58(5), 281-92.

Grunnet, N., Steffensen, R., Varming, K. & Jersild, C. (1996) HLA class I allele typed by PCR-ARMS in Greenlander of Eskimo origin, *Twelfth International Histocompatibility Workshop and Conference*. Paris.

Hajeer, A. H., Sawidan, F. A., Bohlega, S., Saleh, S., Sutton, P., Shubaili, A., Tahan, A. A. & Al Jumah, M. (2009) HLA class I and class II polymorphisms in Saudi patients with myasthenia gravis. *Int J Immunogenet*, 36(3), 169-72.

Hajjej, A., Abdrakhmanova, S., Turganbekova, A. & Almawi, W. Y. (2020a) Distribution of HLA Class I and Class II alleles and haplotypes in German and Uzbek minorities in Kazakhstan, and relationship to other populations. *HLA*, 96(5), 615-620.

Hajjej, A., Abdrakhmanova, S., Turganbekova, A. & Almawi, W. Y. (2021) Origin of the Ukrainian minority of Kazakhstan as inferred from HLA-A, -B, -C, -DRB1, and -DQB1 alleles and haplotypes distribution. *HLA*, 98(6), 525-535.

Hajjej, A., Hmida, S., Kaabi, H., Dridi, A., Jridi, A., El Gaa l ed, A. & Boukef, K. (2006a) HLA genes in Southern Tunisians (Ghannouch area) and their relationship with other Mediterraneans. *Eur J Med Genet*, 49(1), 43-56.

Hajjej, A., Kaabi, H., Sellami, M. H., Dridi, A., Jeridi, A., El borgi, W., Cherif, G., Elgaaied, A., Almawi, W. Y., Boukef, K. & Hmida, S. (2006b) The contribution of HLA class I and II alleles and haplotypes to the investigation of the evolutionary history of Tunisians. *Tissue Antigens*, 68(2), 153-62.

Hajjej, A., Saldhana, F. L., Dajani, R. & Almawi, W. Y. (2020b) HLA-A, -B, -C, -DRB1 and -DQB1 allele and haplotype frequencies and phylogenetic analysis of Bahraini population. *Gene*, 735, 144399.

Hajjej, A., Sellami, M. H., Kaabi, H., Hajjej, G., El-Gaaied, A., Boukef, K., Almawi, W. Y. & Hmida, S. (2011) HLA class I and class II polymorphisms in Tunisian Berbers. *Ann Hum Biol*, 38(2), 156-64.

Hamed, C. T., Meiloud, G., Veten, F., Hadrami, M., Ghaber, S. M., Boussaty, E. C., Habti, N. & Houmeida, A. (2018) HLA class I (-A, -B, -C) and class II (-DR, -DQ) polymorphism in the Mauritanian population. *BMC Med Genet*, 19(1), 2.

Harbo, H. F., Riccio, M. E., Lorentzen, A. R., Utsi, E., Myhr, K. M., Mellgren, S. I., Flam, S. T., Thorsby, E., Sanchez-Mazas, A. & Lie, B. A. (2010) Norwegian Sami differs significantly from other Norwegians according to their HLA profile. *Tissue Antigens*, 75(3), 207-17.

Hidajat, M., Andrien, M., Canck, I. D. & Dupont, E. (1998) Caucasian Belgian Normal, in Terasaki, P. I. & Gjertson, D. W. (eds), *HLA 1998*, 119.

Hoa, B. K., Hang, N. T., Kashiwase, K., Ohashi, J., Lien, L. T., Horie, T., Shojima, J., Hijikata, M., Sakurada, S., Satake, M., Tokunaga, K., Sasazuki, T. & Keicho, N. (2008) HLA-A, -B, -C, -DRB1 and -DQB1 alleles and haplotypes in the Kinh population in Vietnam. *Tissue Antigens*, 71(2), 127-34.

Hollenbach, J. A., Thomson, G., Cao, K., Fernandez-Vina, M., Erlich, H. A., Bugawan, T. L., Winkler, C., Winter, M. & Klitz, W. (2001) HLA diversity, differentiation, and haplotype evolution in Mesoamerican Natives. *Human immunology*, 62(4), 378-90.

Ikeda, N., Kojima, H., Nishikawa, M., Hayashi, K., Futagami, T., Tsujino, T., Kusunoki, Y., Fujii, N., Suegami, S., Miyazaki, Y., Middleton, D., Tanaka, H. & Saji, H. (2015) Determination of HLA-A, -C, -B, -DRB1 allele and haplotype frequency in Japanese population based on family study. *Tissue Antigens*, 85(4), 252-9.

Inotai, D., Szilvasi, A., Benko, S., Boros-Major, A., Illes, Z., Bors, A., Kiss, K. P., Rajczy, K., Gelle-Hosso, A., Buhler, S., Nunes, J. M., Sanchez-Mazas, A. & Tordai, A. (2015) HLA genetic diversity in Hungarians and Hungarian Gypsies: complementary differentiation patterns and demographic signals revealed by HLA-A, -B and -DRB1 in Central Europe. *Tissue Antigens*, 86(2), 115-21.

Ivanova, M., Rozemuller, E., Tyufekchiev, N., Michailova, A., Tilanus, M. & Naumova, E. (2002) HLA polymorphism in Bulgarians defined by high-resolution typing methods in comparison with other populations. *Tissue Antigens*, 60(6), 496-504.

Jersild, C. & Steffensen, R. (1997a) Danish Normal, in Terasaki, P. I. & Gjertson, D. W. (eds), *HLA 1997*UCLA Tissue Typing Laboratory, 206.

Jersild, C. & Steffensen, R. (1997b) Faroese Normal, in Terasaki, P. I. & Gjertson, D. W. (eds), *HLA 1997*UCLA Tissue Typing Laboratory, 207.

Jinam, T. A., Saitou, N., Edo, J., Mahmood, A. & Phipps, M. E. (2010) Molecular analysis of HLA Class I and Class II genes in four indigenous Malaysian populations. *Tissue Antigens*, 75(2), 151-8.

Johansson, A., Ingman, M., Mack, S. J., Erlich, H. & Gyllensten, U. (2008) Genetic origin of the Swedish Sami inferred from HLA class I and class II allele frequencies. *Eur J Hum Genet*, 16(11), 1341-9.

Khansa, S., Hoteit, R., Shammaa, D., Khalek, R. A., El Halas, H., Greige, L., Abbas, F. & Mahfouz, R. A. (2013) HLA class I allele frequencies in the Lebanese population. *Gene*, 512(2), 560-5.

Kijak, G. H., Walsh, A. M., Koehler, R. N., Moqueet, N., Eller, L. A., Eller, M., Currier, J. R., Wang, Z., Wabwire-Mangen, F., Kibuuka, H. N., Michael, N. L., Robb, M. L. & McCutchan, F. E. (2009) HLA class I allele and haplotype diversity in Ugandans supports the presence of a major east African genetic cluster. *Tissue Antigens*, 73(3), 262-9.

Koehler, R. N., Walsh, A. M., Sanders-Buell, E. E., Eller, L. A., Eller, M., Currier, J. R., Bautista, C. T., Wabwire-Mangen, F., Hoelscher, M., Maboko, L., Kim, J., Michael, N. L., Robb, M. L., McCutchan, F. E. & Kijak, G. H. (2010) High-throughput high-resolution class I HLA genotyping in East Africa. *PLoS One*, 5(5), e10751.

Kupatawintu, P., Pheancharoen, S., Srisuddee, A., Tanaka, H., Tadokoro, K. & Nathalang, O. (2010) HLA-A, -B, -DR haplotype frequencies in the Thai Stem Cell Donor Registry. *Tissue Antigens*, 75(6), 730-6.

Larcombe, L. A., Shafer, L. A., Nickerson, P. W., Lodge, A. M., Brown, J. S., Milligan, L. C., Pochinco, D., Beaudin, L., Arundel, B., Wong, J., Dantouze, J., Denechezhe, L. & Orr, P. H. (2017) HLA-A, B, DRB1, DQA1, DQB1 alleles and haplotype frequencies in Dene and Cree cohorts in Manitoba, Canada. *Hum Immunol*.

Lazaro, A. M., Moraes, M. E., Marcos, C. Y., Moraes, J. R., Fernandez-Vina, M. A. & Stastny, P. (1999) Evolution of HLA-class I compared to HLA-class II polymorphism in Terena, a South-American Indian tribe. *Human immunology*, 60(11), 1138-49.

Lee, K. W., Oh, D. H., Lee, C. & Yang, S. Y. (2005) Allelic and haplotypic diversity of HLA-A, -B, -C, -DRB1, and -DQB1 genes in the Korean population. *Tissue Antigens*, 65(5), 437-47.

Leffell, M. S., Cherikh, W. S., Land, G. & Zachary, A. A. (2007) Improved definition of human leukocyte antigen frequencies among minorities and applicability to estimates of transplant compatibility. *Transplantation*, 83(7), 964-72.

Leffell, M. S., Fallin, M. D., Erlich, H. A., Fernandez-Vijna, M., Hildebrand, W. H., Mack, S. J. & Zachary, A. A. (2002) HLA antigens, alleles and haplotypes among the Yup'ik Alaska natives: report of the ASHI Minority Workshops, Part II. *Hum Immunol*, 63(7), 614-25.

Leffell, M. S., Fallin, M. D., Hildebrand, W. H., Cavett, J. W., Iglehart, B. A. & Zachary, A. A. (2004) HLA alleles and haplotypes among the Lakota Sioux: report of the ASHI minority workshops, part III. *Human immunology*, 65(1), 78-89.

Loginova, M., Smirnova, D., Kutyavina, S., Makhova, O., Kashin, K. & Paramonov, I. (2021) Kalmyks from Republic of Kalmykia, Russia. *HLA*, 97(2), 177-179.

Lou, H., Li, H. C., Kuwayama, M., Yashiki, S., Fujiyoshi, T., Suehara, M., Osame, M., Yamashita, M., Hayami, M., Gurtsevich, V., Ballas, M., Imanishi, T. & Sonoda, S. (1998) HLA class I and class II of the Nivkhi, an indigenous population carrying HTLV-I in Sakhalin, Far Eastern Russia. *Tissue Antigens*, 52(5), 444-51.

Machulla, H. K., Batnasan, D., Steinborn, F., Uyar, F. A., Saruhan-Direskeneli, G., Oguz, F. S., Carin, M. N. & Dorak, M. T. (2003) Genetic affinities among Mongol ethnic groups and their relationship to Turks. *Tissue Antigens*, 61(4), 292-9.

Mack, S., Tsai, Y., Sanchez-Mazas, A., Erlich, HA. (2007) Chapter 3: Anthropology/ human genetic diversity population reports. 13th International Histocompatibility Workshop Anthropology/Human Genetic Diversity Joint Report, in Hansen, J. A. (ed), *Immunobiology of the human MHC: Proceedings of the 13th International Histocompatibility Workshop and Conference*. Seattle, WA: IHWG Press, 580-652.

Maiers, M., Gragert, L. & Klitz, W. (2007) High-resolution HLA alleles and haplotypes in the United States population. *Hum Immunol*, 68(9), 779-88.

Main, P., Attenborough, R., Chelvanayagam, G., Bhatia, K. & Gao, X. (2001) The peopling of New Guinea: evidence from class I human leukocyte antigen. *Hum Biol*, 73(3), 365-83.

Malavige, G. N., Rostron, T., Seneviratne, S. L., Fernando, S., Sivayogan, S., Wijewickrama, A. & Ogg, G. S. (2007) HLA analysis of Sri Lankan Sinhalese predicts North Indian origin. *Int J Immunogenet*, 34(5), 313-5.

Manor, S., Halagan, M., Shriki, N., Yaniv, I., Zisser, B., Maiers, M., Madbouly, A. & Stein, J. (2016) High-resolution HLA A approximately B approximately DRB1 haplotype frequencies from the Ezer Mizion Bone Marrow Donor Registry in Israel. *Hum Immunol*, 77(12), 1114-1119.

Martinez-Laso, J., Siles, N., Moscoso, J., Zamora, J., Serrano-Vela, J. I., JI, R. A.-C., Castro, M. J., Serrano-Rios, M. & Arnaiz-Villena, A. (2006) Origin of Bolivian Quechua Amerindians: their relationship with other American Indians and Asians according to HLA genes. *European journal of medical genetics*, 49(2), 169-85.

Matevosyan, L., Chattopadhyay, S., Madelian, V., Avagyan, S., Nazaretyan, M., Hyussian, A., Vardapetyan, E., Arutunyan, R. & Jordan, F. (2011) HLA-A, HLA-B, and HLA-DRB1 allele distribution in a large Armenian population sample. *Tissue Antigens*, 78(1), 21-30.

Middleton, D., Williams, F., Meenagh, A., Daar, A. S., Gorodezky, C., Hammond, M., Nascimento, E., Briceno, I. & Perez, M. P. (2000) Analysis of the distribution of HLA-A alleles in populations from five continents. *Hum Immunol*, 61(10), 1048-52.

Modiano, D., Luoni, G., Petrarca, V., Sodiomon Sirima, B., De Luca, M., Simpore, J., Coluzzi, M., Bodmer, J. G. & Modiano, G. (2001) HLA class I in three West African ethnic groups: genetic distances from sub-Saharan and Caucasoid populations. *Tissue Antigens*, 57(2), 128-37.

Mohyuddin, A., Ayub, Q., Khaliq, S., Mansoor, A., Mazhar, K., Rehman, S. & Mehdi, S. Q. (2002) HLA polymorphism in six ethnic groups from Pakistan. *Tissue Antigens*, 59(6), 492-501.

Moscoso, J., Seclen, S., Serrano-Vela, J. I., Villena, A., Martinez-Laso, J., Zamora, J., Moreno, A., Ira-Cachafeiro, J. & Arnaiz-Villena, A. (2006) HLA genes in Lamas Peruvian-Amazonian Amerindians. *Mol Immunol*, 43(11), 1881-9.

Norman, P. J., Hollenbach, J. A., Nemat-Gorgani, N., Guethlein, L. A., Hilton, H. G., Pando, M. J., Koram, K. A., Riley, E. M., Abi-Rached, L. & Parham, P. (2013) Co-evolution of human leukocyte antigen (HLA) class I ligands with killer-cell immunoglobulin-like receptors (KIR) in a genetically diverse population of sub-Saharan Africans. *PLoS Genet*, 9(10), e1003938.

Nunes, J. M., Buhler, S., Roessli, D., Sanchez-Mazas, A. & collaboration, H. L.-n. (2014) The HLA-net GENE[RATE] pipeline for effective HLA data analysis and its application to 145 population samples from Europe and neighbouring areas. *Tissue Antigens*, 83(5), 307-23.

Paximadis, M., Mathebula, T. Y., Gentle, N. L., Vardas, E., Colvin, M., Gray, C. M., Tiemessen, C. T. & Puren, A. (2012) Human leukocyte antigen class I (A, B, C) and II (DRB1) diversity in the black and Caucasian South African population. *Hum Immunol*, 73(1), 80-92.

Piancatelli, D., Canossi, A., Aureli, A., Oumhani, K., Del Beato, T., Di Rocco, M., Liberatore, G., Tessitore, A., Witter, K., El Aouad, R. & Adorno, D. (2004) Human leukocyte antigen-A, -B, and -Cw polymorphism in a Berber population from North Morocco using sequence-based typing. *Tissue Antigens*, 63(2), 158-72.

Pingel, J., Solloch, U. V., Hofmann, J. A., Lange, V., Ehninger, G. & Schmidt, A. H. (2013) High-resolution HLA haplotype frequencies of stem cell donors in Germany with foreign parentage: how can they be used to improve unrelated donor searches? *Hum Immunol*, 74(3), 330-40.

Rey, D., Amirzargar, A., Areces, C., Enriquez-de-Salamanca, M., Marco, J., Abd-El-Fatah-Khalil, S., Fernandez-Honrado, M., Muniz, E., Martin-Villa, J. M. & Arnaiz-Villena, A. (2015) Gorgan (Turkmen in Iran) HLA genetics: transplantation, pharmacogenomics and anthropology. *Immunol Invest*, 44(1), 88-100.

Rey, D., Areces, C., Alonso-Rubio, J., Enriquez-de-Salamanca, M., Abd-El-Fatah-Khalil, S., Bendikuze, N., Fernandez-Honrado, M., Barbolla, L., Martin-Villa, J. M. & Arnaiz-Villena, A. (2013a) HLA in Georgians (Caucasus) and their relationship with Eastern Mediterraneans. *Mol Biol Rep*, 40(10), 5523-30.

Rey, D., Parga-Lozano, C., Moscoso, J., Areces, C., Enriquez-de-Salamanca, M., Fernandez-Honrado, M., Abd-El-Fatah-Khalil, S., Alonso-Rubio, J. & Arnaiz-Villena, A. (2013b) HLA genetic profile of Mapuche (Araucanian) Amerindians from Chile. *Mol Biol Rep*, 40(7), 4257-67.

Romon, I., Montes, C., Ligeiro, D., Trindade, H., Sanchez-Mazas, A., Nunes, J. M. & Buhler, S. (2016) Mapping the HLA diversity of the Iberian Peninsula. *Hum Immunol*, 77(10), 832-40.

Saldanha, N., Spínola, C., Santos, MR., Simoes, JP., Bruges-Armas, J., Brehm, A., Spinola, H. (2009) HLA polymorphisms in Forros and Angolares from Sao Tome Island (West Africa): evidence for the population origin. *Journal of Genetic Genealogy*, 5(2), 76-85.

Sanchez-Mazas, A., Steiner, Q. G., Grundschober, C. & Tiercy, J. M. (2000) The molecular determination of HLA-Cw alleles in the Mandenka (West Africa) reveals a close genetic relationship between Africans and Europeans. *Tissue Antigens*, 56(4), 303-12.

Sanchez-Velasco, P., Gomez-Casado, E., Martinez-Laso, J., Moscoso, J., Zamora, J., Lowy, E., Silvera, C., Cemborain, A., Leyva-Cobian, F. & Arnaiz-Villena, A. (2003) HLA alleles in isolated populations from North Spain: origin of the Basques and the ancient Iberians. *Tissue Antigens*, 61(5), 384-92.

Schmidt, A. H., Solloch, U. V., Pingel, J., Baier, D., Bohme, I., Dubicka, K., Schumacher, S., Rutt, C., Skotnicki, A. B., Wachowiak, J. & Ehninger, G. (2011) High-resolution human leukocyte antigen allele and haplotype frequencies of the Polish population based on 20,653 stem cell donors. *Hum Immunol*, 72(7), 558-65.

Shen, C. M., Zhu, B. F., Deng, Y. J., Ye, S. H., Yan, J. W., Yang, G., Wang, H. D., Qin, H. X., Huang, Q. Z. & Zhang, J. J. (2010) Allele polymorphism and haplotype diversity of HLA-A, -B and -DRB1 loci in sequence-based typing for Chinese Uyghur ethnic group. *PLoS One*, 5(11), e13458.

Silvera, C., Vargas-Alarcon, G., Areces, C., Rey, D., Parga-Lozano, C., Gomez-Prieto, P., Barbolla, L., Martinez-Laso, J. & Arnaiz-Villena, A. (2011) HLA genes in Wayu Amerindians from Colombia. *Immunol Invest*, 40(1), 92-100.

Smigoc Schweiger, D., Mendez, A., Kunilo Jamnik, S., Bratanic, N., Bratina, N., Battelino, T., Brecelj, J. & Vidan-Jeras, B. (2014) Genetic risk for co-occurrence of type 1 diabetes and celiac disease is modified by HLA-C and killer immunoglobulin-like receptors. *Tissue Antigens*, 84(5), 471-8.

Spinola, H., Bruges-Armas, J., Middleton, D. & Brehm, A. (2005) HLA polymorphisms in Cabo Verde and Guine-Bissau inferred from sequence-based typing. *Human immunology*, 66(10), 1082-92.

Sulcebe, G., Sanchez-Mazas, A., Tiercy, J. M., Shyti, E., Mone, I., Ylli, Z. & Kardhashi, V. (2009) HLA allele and haplotype frequencies in the Albanian population and their relationship with the other European populations. *Int J Immunogenet*, 36(6), 337-43.

Suslova, T. A., Burmistrova, A. L., Chernova, M. S., Khromova, E. B., Lupar, E. I., Timofeeva, S. V., Devald, I. V., Vavilov, M. N. & Darke, C. (2012) HLA gene and haplotype frequencies in Russians, Bashkirs and Tatars, living in the Chelyabinsk Region (Russian South Urals). *Int J Immunogenet*, 39(5), 394-408.

Tang, J., Naik, E., Costello, C., Karita, E., Rivers, C., Allen, S. & Kaslow, R. A. (2000) Characteristics of HLA class I and class II polymorphisms in Rwandan women. *Experimental and clinical immunogenetics*, 17(4), 185-98.

Testi, M., Lai, S., Orru, S., Alba, F., Cappai, L., Firdous, N., Gaziev, J., Troiano, M., Andreani, M. & Carcassi, C. (2011) Distribution of HLA alleles and haplotypes in the Maldivian population. *Tissue Antigens*, 77(3), 235-8.

Tonks, S., Bodmer, J. & Bodmer, W. HLA-A, -B, -Cw, -DQA1 and -DRB1 allele frequencies in a population from Orkney, Scotland. *Human immunology*, 65, 1069 - 1071.

Torimiro, J. N., Carr, J. K., Wolfe, N. D., Karacki, P., Martin, M. P., Gao, X., Tamoufe, U., Thomas, A., Ngole, E. M., Birx, D. L., McCutchan, F. E., Burke, D. S. & Carrington, M. (2006) HLA class I diversity among rural rainforest inhabitants in Cameroon: identification of A*2612-B*4407 haplotype. *Tissue Antigens*, 67(1), 30-7.

Vargas-Alarcon, G., Hernandez-Pacheco, G., Moscoso, J., Perez-Hernandez, N., Murguia, L. E., Moreno, A., Serrano-Vela, J. I., Granados, J. & Arnaiz-Villena, A. (2006) HLA genes in Mexican Teeneks: HLA genetic relationship with other worldwide populations. *Mol Immunol*, 43(7), 790-9.

Wang, H. D., Jin, X. Y., Yin, S. S., Zhang, Q., Su, J. X., Shen, C. M. & Zhu, B. F. (2021) Diversities of HLA-A, -B, -C, -DRB1 and -DQB1 loci in Chinese Kazak population and its genetic relatedness dissection with multiple populations: a comparative study. *Hum Immunol*, 82(4), 215-225.

Williams, F., Meenagh, A., Darke, C., Acosta, A., Daar, A. S., Gorodezky, C., Hammond, M., Nascimento, E. & Middleton, D. (2001) Analysis of the distribution of HLA-B alleles in populations from five continents. *Hum Immunol*, 62(6), 645-50.

Williams, R., Chen, Y. F., Endres, R., Middleton, D., Trucco, M., Williams, J. D. & Knowler, W. (2009) Molecular variation at the HLA-A, B, C, DRB1, DQA1, and DQB1 loci in full heritage American Indians in Arizona: private haplotypes and their evolution. *Tissue Antigens*, 74(6), 520-33.

Yao, Y., Shi, L., Tao, Y., Kulski, J. K., Lin, K., Huang, X., Xiang, H., Chu, J. & Shi, L. (2012) Distinct HLA allele and haplotype distributions in four ethnic groups of China. *Tissue Antigens*, 80(5), 452-61.

Yuliwulandari, R., Sachrowardi, Q., Nakajima, H., Kashiwase, K., Hirayasu, K., Mabuchi, A., Sofro, A. S. & Tokunaga, K. (2010) Association of HLA-A, -B, and -DRB1 with pulmonary tuberculosis in western Javanese Indonesia. *Hum Immunol*, 71(7), 697-701.

**References for HLA-DRB1**

Alfirevic, A., Gonzalez-Galarza, F., Bell, C., Martinsson, K., Platt, V., Bretland, G., et al. (2012). In silico analysis of HLA associations with drug-induced liver injury: Use of a HLA-genotyped DNA archive from healthy volunteers. Genome Med. 4, 51. doi:10.1186/gm350.

Andrien, M., and Dupont, E. (2004). HLA-B and -DRB1 allele frequencies in a population from Dakar, Senegal. Hum. Immunol. 65, 1071–1072.

Arlehamn, C. S. L., Copin, R., Leary, S., Mack, S. J., Phillips, E., Mallal, S., et al. (2017). Sequence-based HLA-A, B, C, DP, DQ, and DR typing of 100 Luo infants from the Boro area of Nyanza Province, Kenya. Hum. Immunol. 78, 325–326. doi:10.1016/j.humimm.2017.03.007.

Arnaiz-Villena, A., Benmamar, D., Alvarez, M., Diaz-Campos, N., Varela, P., Gomez-Casado, E., et al. (1995). HLA allele and haplotype frequencies in Algerians. Relatedness to Spaniards and Basques. Hum. Immunol. 43, 259–268. doi:10.1016/0198-8859(95)00024-X.

Arnaiz-Villena, A., Dimitroski, K., Pacho, A., Moscoso, J., Gómez-Casado, E., Silvera-Redondo, C., et al. (2001a). HLA genes in Macedonians and the sub-Saharan origin of the Greeks. Tissue Antigens 57, 118–127. doi:10.1034/j.1399-0039.2001.057002118.x.

Arnaiz-Villena, A., Elaiwa, N., Silvera, C., Rostom, A., Moscoso, J., Gómez-Casado, E., et al. (2001b). The origin of Palestinians and their genetic relatedness with other Mediterranean populations. Hum. Immunol. 62, 889–900. doi:10.1016/S0198-8859(01)00288-9.

Arnaiz-Villena, A., Gonzalez-Alcos, V., Serrano-Vela, J. I., Reguera, R., Barbolla, L., Parga-Lozano, C., et al. (2009a). HLA genes in Uros from Titikaka Lake, Peru: Origin and relationship with other Amerindians and worldwide populations. Int. J. Immunogenet. 36, 159–167. doi:10.1111/j.1744-313X.2009.00841.x.

Arnaiz-Villena, A., Lopez-Nares, A., Callado, A., H-Sevilla, A., Rashidi, F., Palacio-Grüber, J., et al. (2019). Study of HLA genes in Russia Bering Island Aleuts. Hum. Immunol. 80, 631–632. doi:10.1016/j.humimm.2019.07.295.

Arnaiz-Villena, A., Martinez-Laso, J., Moscoso, J., Livshits, G., Zamora, J., Gomez-Casado, E., et al. (2003). HLA Genes in the Chuvashian Population from European Russia: Admixture of CentralEuropean and Mediterranean Populations. Hum. Biol. 75, 375–392.

Arnaiz-Villena, A., Moscoso, J., Granados, J., Serrano-Vela, J. I., de la Pena, A., Reguera, R., et al. (2007). HLA Genes in Mayos Population from Northeast Mexico. Curr Genomics 8, 466–475. doi:10.2174/138920207783591735.

Arnaiz-Villena, A., Reguera, R., Ferri, A., Barbolla, L., Abd-El-Fatah-Khalil, S., Bakhtiyarova, N., et al. (2009b). The peopling of Madeira archipelago (Portugal) according to HLA genes. Int. J. Immunogenet. 36, 9–14. doi:10.1111/j.1744-313X.2008.00813.x.

Arnaiz-Villena, A., Siles, N., Moscoso, J., Zamora, J., Serrano-Vela, J. I., Gomez-Casado, E., et al. (2005). Origin of Aymaras from Bolivia and their relationship with other Amerindians according to HLA genes. Tissue Antigens 65, 379–390. doi:10.1111/j.1399-0039.2005.00356.x.

Arnaiz-Villena, A., Vargas-Alarcón, G., Granados, J., Gómez-Casado, E., Longas, J., Gonzalez-Hevilla, M., et al. (2000). HLA genes in Mexican Mazatecans, the peopling of the Americas and the uniqueness of Amerindians. Tissue Antigens 56, 405–416. doi:10.1034/j.1399-0039.2000.560503.x.

Assane, A. A. A., Fabricio-Silva, G. M., Cardoso-Oliveira, J., Mabunda, N. E. J., Sousa, A. M., Jani, I. V., et al. (2010). Human leukocyte antigen-A, -B, and -DRB1 allele and haplotype frequencies in the Mozambican population: A blood donor-based population study. Hum. Immunol. 71, 1027–1032. doi:10.1016/j.humimm.2010.06.017.

Bannai, M., Ohashi, J., Harihara, S., Takahashi, Y., Juji, T., Omoto, K., et al. (2000). Analysis of HLA genes and haplotypes in Ainu (from Hokkaido, northern Japan) supports the premise that they descent from Upper Paleolithic populations of East Asia. Tissue Antigens 55, 128–139. doi:10.1034/j.1399-0039.2000.550204.x.

Barquera, R., Zuniga, J., Flores-Rivera, J., Corona, T., Penman, B. S., Hernández-Zaragoza, D. I., et al. (2020). Diversity of HLA Class I and Class II blocks and conserved extended haplotypes in Lacandon Mayans. Sci. Rep. 10, 3248. doi:10.1038/s41598-020-58897-5.

Begovich, A. B., Moonsamy, P. V, Mack, S. J., Barcellos, L. F., Steiner, L. L., Grams, S., et al. (2001). Genetic variability and linkage disequilibrium within the HLA-DP region : analysis of 15 different populations. Tissue Antigens 57, 424–439.

Benitez, O., Busson, M., Charron, D., and Loiseau, P. (2011). HLA polymorphism in a Guarani-Indian population from Paraguay and its usefulness for the Hispano-Indian admixture study in Paraguay. Int. J. Immunogenet. 38, 7–11. doi:10.1111/j.1744-313X.2010.00959.x.

Canossi, A., Piancatelli, D., Aureli, A., Oumhani, K., Ozzella, G., Del Beato, T., et al. (2010). Correlation between genetic HLA class i and II polymorphisms and anthropological aspects in the Chaouya population from Morocco (Arabic speaking). Tissue Antigens 76, 177–193. doi:10.1111/j.1399-0039.2010.01498.x.

Cao, K., Lyke, K. E., Masaberg, C., Doumbo, O. K., Ng, J., Hartzman, R. J., et al. (2007). “Anthropology/human genetic diversity population reports. Malian Admixed from Mali,” in Immunobiology of the Human MHC: Proceedings of the 13th International Histocompatibility Workshop and Conference, Volume I, ed. J. A. Hansen (Seattle: IHWG Press), 583–585.

Cao, K., Moormann, A. M., Lyke, K. E., Masaberg, C., Sumba, O. P., Doumbo, O. K., et al. (2004). Differentiation between African populations is evidenced by the diversity of alleles and haplotypes of HLA class I loci. Tissue Antigens 63, 293–325. doi:10.1111/j.0001-2815.2004.00192.x.

Cerna, M., Falco, M., Friedman, H., Raimondi, E., Maccagno, A., Fernandez-Vina, M., et al. (1993). Differences in HLA class II alleles of isolated South American Indian populations from Brazil and Argentina. Hum Immunol 37, 213–220.

Chen, S., Hu, Q., Xie, Y., Zhou, L., Xiao, C., Wu, Y., et al. (2007). Origin of Tibeto-Burman speakers: Evidence from HLA allele distribution in Lisu and Nu inhabiting Yunnan of China. Hum. Immunol. 68, 550–559. doi:10.1016/j.humimm.2007.02.006.

Chu, C., Trejaut, J., Lee, H., Chang, S., and Lin, M. (2007a). “Ivatan from Bantanes, Philippines. Anthropology/human genetic diversity population reports,” in Immunobiology of the Human MHC: Proceedings of the 13th International Histocompatibility Workshop and Conference. Volume I, ed. J. A. Hansen (Seattle: IHWG Press), 611–615.

Chu, C., Trejaut, J., Lee, H., Chang, S., and Lin, M. (2007b). “Population anthropology/human genetic diversity population reports. Ami from Hualien/Taitung; Minnan; Atayal from Wulai/Chenshih/Wufen; Bunun from Hsin-I/Taitung; Hakka from Hsinchu/Pintung; Puyuma from Peinan; Saisiat from Wufen / Nanchuang, Taiwan,” in Immunobiology of the Human MHC: Proceedings of the 13th International Histocompatibility Workshop and Conference, Volume I, ed. J. A. Hansen (Seattle: IHWG Press), 611–615.

Crespí, C., Milà, J., Martínez-Pomar, N., Etxagibel, A., Muñoz-Saa, I., Priego, D., et al. (2002). HLA polymorphism in a Majorcan population of Jewish descent : Comparison with Majorca , Minorca , Ibiza ( Balearic Islands ) and other Jewish communities. Tissue Antigens 60, 282–291.

Dhaliwal, J. S., Shahnaz, M., Too, C. L., Azrena, A., Maiselamah, L., Lee, Y. Y., et al. (2007). HLA-A, -B and -DR allele and haplo-type frequencies in Malays. Asian Pacific J. Allergy Immunol. 25, 47–51.

Dunne, C., Crowley, J., Hagan, R., Rooney, G., and Lawlor, E. (2008). HLA-A, B, Cw, DRB1, DQB1 and DPB1 alleles and haplotypes in the genetically homogenous Irish population. Int. J. Immunogenet. 35, 295–302. doi:10.1111/j.1744-313X.2008.00779.x.

Edinur, H. A., Dunn, P. P. J., Hammond, L., Selwyn, C., Brescia, P., Askar, M., et al. (2013). HLA and MICA polymorphism in Polynesians and New Zealand Maori: Implications for ancestry and health. Hum. Immunol. 74, 1119–1129. doi:10.1016/j.humimm.2013.06.011.

Erlich, H., Alejandrino, M., Pozzili, P., Panelo, A., and Bugawan, T. (2007). “Population Anthropology/human genetic diversity population reports. Filipino from Luzon Island, Philippines,” in Immunobiology of the Human MHC: Proceedings of the 13th International Histocompatibility Workshop and Conference, Volume I, ed. J. A. Hansen (Seattle: IHWG Press), 620–621.

Farjadian, S., Naruse, T., Kawata, H., Ghaderi, A., Bahram, S., and Inoko, H. (2004). Molecular analysis of HLA allele frequencies and haplotypes in Baloch of Iran compared with related populations of Pakistan. Tissue Antigens 64, 581–587. doi:10.1111/j.1399-0039.2004.00302.x.

Ferencik, S., Gong, F., and Grosse-Wilde, H. (1998). “HLA gene frequencies: Chinese (Wuhan) Normal,” in HLA 1998, eds. P. I. Terasaki and D. Gjertson (Lenexa: UCLA Tissue Typing Laboratory (UCLA)), 202–203.

Galgani, A., Mancino, G., Martínez-Labarga, C., Cicconi, R., Mattei, M., Amicosante, M., et al. (2013). HLA-A, -B and -DRB1 allele frequencies in Cyrenaica population (Libya) and genetic relationships with other populations. Hum. Immunol. 74, 52–59. doi:10.1016/j.humimm.2012.10.001.

Gao, X., Bhatia, K., Trent, R. J., and Serjeantson, S. W. (1992). HLA-DR,DQ nucleotide sequence polymorphisms in five Melanesian populations. Tissue Antigens 40, 31–37. doi:10.1111/j.1399-0039.1992.tb01954.x.

Gao, X., Mack, S. J., Currie, B., Asteal, S., and McCluskey, J. (2007). “Anthropology/human genetic diversity population reports. Aboriginal Australian from Cape York, Australia,” in Immunobiology of the Human MHC: Proceedings of the 13th International Histocompatibility Workshop and Conference, Volume I, ed. J. A. Hansen (Seattle: IHWG Press), 624–62.

García-Ortiz, J. E., Sandoval-Ramírez, L., Rangel-Villalobos, H., Maldonado-Torres, H., Cox, S., García-Sepúlveda, C. A., et al. (2006). High-resolution molecular characterization of the HLA class I and class II in the Tarahumara Amerindian population. Tissue Antigens 68, 135–146. doi:10.1111/j.1399-0039.2006.00636.x.

Gómez-Casado, E., Del Moral, P., Martinez-Laso, J., García-Gómez, A., Allende, L., Silvera-Redondo, C., et al. (2000). HLA genes in Arabic-speaking Moroccans: Close relatedness to Berbers and Iberians. Tissue Antigens 55, 239–249. doi:10.1034/j.1399-0039.2000.550307.x.

Gómez-Casado, E., Martínez-Laso, J., Moscoso, J., Zamora, J., Martin-Villa, M., Perez-Blas, M., et al. (2003). Origin of Mayans according to HLA genes and the uniqueness of Amerindians. Tissue Antigens 61, 425–436. doi:10.1034/j.1399-0039.2003.00040.x.

Gonzalez-Galarza, F. F., McCabe, A., Santos, E. J. M. Dos, Jones, J., Takeshita, L., Ortega-Rivera, N. D., et al. (2020). Allele frequency net database (AFND) 2020 update: gold-standard data classification, open access genotype data and new query tools. Nucleic Acids Res. 48, D783–D788. doi:10.1093/nar/gkz1029.

Gourraud, P. A., Khankhanian, P., Cereb, N., Yang, S. Y., Feolo, M., Maiers, M., et al. (2014). HLA diversity in the 1000 genomes dataset. PLoS One 9. doi:10.1371/journal.pone.0097282.

Hajjej, A., Hajjej, G., Almawi, W. Y., Kaabi, H., El-Gaaied, A., and Hmida, S. (2011). HLA class I and class II polymorphism in a population from south-eastern Tunisia (Gabes area). Int. J. Immunogenet. 38, 191–199. doi:10.1111/j.1744-313X.2011.01003.x.

Hajjej, A., Hmida, S., Kaabi, H., Dridi, A., Jridi, A., El Gaald, A., et al. (2006). HLA genes in southern Tunisians (Ghannouch area) and their relationship with other Mediterraneans. Eur. J. Med. Genet. 49, 43–56. doi:10.1016/j.ejmg.2005.01.001.

Harbo, H. F., Riccio, M. E., Lorentzen, Å. R., Utsi, E., Myhr, K. M., Mellgren, S. I., et al. (2010). Norwegian Sami differs significantly from other Norwegians according to their HLA profile. Tissue Antigens 75, 207–217. doi:10.1111/j.1399-0039.2009.01425.x.

Hoa, B. K., Hang, N. T. L., Kashiwase, K., Ohashi, J., Lien, L. T., Horie, T., et al. (2008). HLA-A, -B, -C, -DRB1 and -DQB1 alleles and haplotypes in the Kinh population in Vietnam. Tissue Antigens 71, 127–134. doi:10.1111/j.1399-0039.2007.00982.x.

Hollenbach, J. A., Thomson, G., Cao, K., Fernandez-Vina, M., Erlich, H. A., Bugawan, T. L., et al. (2001). HLA diversity, differentiation, and haplotype evolution in Mesoamerican Natives. Hum. Immunol. 62, 378–390. doi:10.1016/s0198-8859(01)00212-9.

Huh, J. Y., Yi, D. Y., Eo, S. H., Cho, H., Park, M. H., and Kang, M. S. (2013). HLA-A, -B and -DRB1 polymorphism in koreans defined by sequence-based typing of 4128 cord blood units. Int. J. Immunogenet. 40, 515–523. doi:10.1111/iji.12067.

Infante, E., Aláez, C., Flores, H., and Gorodezky, C. (2007). “Seri from Sonora, Mexico. Anthropology/human genetic diversity population reports,” in Immunobiology of the Human MHC: Proceedings of the 13th International Histocompatibility Workshop and Conference. Volume I, ed. J. A. Hansen (Seattle: IHWG Press), 633–634.

Ivanova, M., Rozemuller, E., Tyufekchiev, N., Michailova, A., Tilanus, M., and Naumova, E. (2002). HLA polymorphism in Bulgarians defined by high-resolution typing methods in comparison with other populations. Tissue Antigens 60, 496–504. doi:10.1034/j.1399-0039.2002.600605.x.

Ivaskova, E., and Bendukidze, N. (2007). “Anthropology/human genetic diversity population reports. Czech from Prague, Czech Republic,” in Immunobiology of the Human MHC: Proceedings of the 13th International Histocompatibility Workshop and Conference, Volume I, ed. J. A. Hansen (Seattle: IHWG Press), 597–598.

Jinam, T. A., Saitou, N., Edo, J., Mahmood, A., and Phipps, M. E. (2010). Molecular analysis of HLA Class i and Class II genes in four indigenous Malaysian populations. Tissue Antigens 75, 151–158. doi:10.1111/j.1399-0039.2009.01417.x.

Johansson, Å., Ingman, M., Mack, S. J., Erlich, H., and Gyllensten, U. (2008). Genetic origin of the Swedish Sami inferred from HLA class I and class II allele frequencies. Eur. J. Hum. Genet. 16, 1341–1349. doi:10.1038/ejhg.2008.88.

Kupatawintu, P., Pheancharoen, S., Srisuddee, A., Tanaka, H., Tadokoro, K., and Nathalang, O. (2010). HLA-A, -B, -DR haplotype frequencies in the Thai Stem Cell Donor Registry. Tissue Antigens 75, 730–736. doi:10.1111/j.1399-0039.2010.01450.x.

Kwok, J., Guo, M., Yang, W., Lee, C. K., Chan, N. K., Ho, J., et al. (2016). HLA-A, -B and -DRB1 genotyping and haplotype frequencies of 3892 cord blood units in the Hong Kong Chinese Cord Blood Registry. Hum. Immunol. 77, 1109–1110. doi:https://doi.org/10.1016/j.humimm.2016.10.006.

Layrisse, Z., Guedez, Y., Domínguez, E., Paz, N., Montagnani, S., Matos, M., et al. (2001). Extended HLA haplotypes in a Carib Amerindian population: The Yucpa of the Perija Range. Hum. Immunol. 62, 992–1000. doi:10.1016/S0198-8859(01)00297-X.

Lázaro, A. M., Moraes, M. E., Marcos, C. Y., Moraes, J. R., Fernández-Viña, M. A., and Stastny, P. (1999). Evolution of HLA-class I compared to HLA-class II polymorphism in Terena, a South-American Indian tribe. Hum. Immunol. 60, 1138–1149. doi:10.1016/S0198-8859(99)00092-0.

Lee, K. W., Oh, D. H., Lee, C., and Yang, S. Y. (2005). Allelic and haplotypic diversity of HLA-A, -B, -C, -DRB1, and -DQB1 genes in the Korean population. Tissue Antigens 65, 437–447. doi:10.1111/j.1399-0039.2005.00386.x.

Leffell, M. S., Fallin, M. D., Erlich, H. A., Fernandez-Vĩna, M., Hildebrand, W. H., Mack, S. J., et al. (2002). HLA antigens, alleles and haplotypes among the Yup’ik Alaska natives: Report of the ASHI Minority Workshops, part II. Hum. Immunol. 63, 614–625. doi:10.1016/S0198-8859(02)00415-9.

Leffell, M. S., Fallin, M. D., Hildebrand, W. H., Cavett, J. W., Iglehart, B. A., and Zachary, A. A. (2004). HLA alleles and haplotypes among the lakota sioux: Report of the ASHI minority workshops, part III. Hum. Immunol. 65, 78–89. doi:10.1016/j.humimm.2003.10.001.

Louie, L., Mather, K., Meyer, D., Hollenbach, J., Jackman, R., Schultz, K., et al. (2007). “Population Anthropology/human genetic diversity population reports. Shona from Harare, Zimbabwe,” in Immunobiology of the Human MHC: Proceedings of the 13th International Histocompatibility Workshop and Conference, Volume I, ed. J. A. Hansen (Seattle: IHWG Press), 587–589.

Luiza, M., Erler, P., Luz, R., and Sotomaior, V. S. (1993). The HLA polymorphsm of two distinctive South‐American Indian tribes: The Kaingang and the Guarani. Tissue Antigens 41, 227–237. doi:10.1111/j.1399-0039.1993.tb02011.x.

Machulla, H. K. G., Batnasan, D., Steinborn, F., Uyar, F. A., Saruhan-Direskeneli, G., Oguz, F. S., et al. (2003). Genetic affinities among Mongol ethnic groups and their relationship to Turks. Tissue Antigens 61, 292–299. doi:10.1034/j.1399-0039.2003.00043.x.

Mack, S., Bugawan, T. L., Moonsamy, P. V, Erlich, J. A., Trachtenberg, E. A., Paik, Y. K., et al. (2000). Evolution of Pacific / Asian populations inferred from HLA class II allele frequency distributions. Tissue Antigens 55, 383–400.

Mack, S. J., Bugawan, T. L., Erlich, J. A., Stoneking, M., Saha, N., and Erlich, H. A. (2007a). “Anthropology/human genetic diversity population reports. Highlander from Papua New Guinea,” in Immunobiology of the Human MHC: Proceedings of the 13th International Histocompatibility Workshop and Conference, Volume I, ed. J. A. Hansen (Seattle: IHWG Press), 621–623.

Mack, S. J., Crawford, M. H., Saha, N., Jani, A. J., Geyer, L. N., Reddy, M., et al. (2007b). “Anthropology/human genetic diversity population reports. Golla from Andrah Pradesh, India,” in Immunobiology of the Human MHC: Proceedings of the 13th International Histocompatibility Workshop and Conference. Volume I, ed. J. A. Hansen (Seattle: IHWG Press), 605–607.

Mack, S. J., Jani, A. J., Below, J. E., Saha, N., and Erlich, H. A. (2007c). “Anthropology/human genetic diversity population reports. Malay from Singapore,” in Immunobiology of the Human MHC: Proceedings of the 13th International Histocompatibility Workshop and Conference, Volume I, ed. J. A. Hansen (Seattle: IHWG Press), 615–616.

Maitland, K., Bunce, M., Harding, R. M., Barnardo, M. C. N. M., Clegg, J. B., Welsh, K., et al. (2004). HLA class-I and class-II allele frequencies and two-locus haplotypes in Melanesians of Vanuatu and New Caledonia. Tissue Antigens 64, 678–686. doi:10.1111/j.1399-0039.2004.00328.x.

Manor, S., Halagan, M., Shriki, N., Yaniv, I., Zisser, B., Maiers, M., et al. (2016). High-resolution HLA A∼B∼DRB1 haplotype frequencies from the Ezer Mizion Bone Marrow Donor Registry in Israel. Hum. Immunol. 77, 1114–1119. doi:10.1016/j.humimm.2016.09.004.

Martinez-Laso, J., Sartakova, M., Allende, L., Konenkov, V., Moscoso, J., Silvera-Redondo, C., et al. (2001). HLA molecular markers in Tuvinians: A population with both Oriental and Caucasoid characteristics. Ann. Hum. Genet. 65, 245–261. doi:10.1017/S0003480001008624.

Martinez-Laso, J., Siles, N., Moscoso, J., Zamora, J., Serrano-Vela, J. I., R-A-Cachafeiro, J. I., et al. (2006). Origin of Bolivian Quechua Amerindians: Their relationship with other American Indians and Asians according to HLA genes. Eur. J. Med. Genet. 49, 169–185. doi:10.1016/j.ejmg.2005.04.005.

Matevosyan, L., Chattopadhyay, S., Madelian, V., Avagyan, S., Nazaretyan, M., Hyussian, A., et al. (2011). HLA-A, HLA-B, and HLA-DRB1 allele distribution in a large Armenian population sample. Tissue Antigens 78, 21–30. doi:10.1111/j.1399-0039.2011.01668.x.

Middleton, D., Williams, F., Hamill, M. A., and Meenagh, A. (2000). Frequency of HLA-B alleles in a caucasoid population determined by a two-stage PCR-SSOP typing strategy. Hum. Immunol. 61, 1285–1297. doi:10.1016/S0198-8859(00)00186-5.

Modiano, D., Luoni, G., Petrarca, V., Sodiomon Sirima, B., De Luca, M., Simporé, J., et al. (2001). HLA class I in three West African ethnic groups: Genetic distances from sub-Saharan and Caucasoid populations. Tissue Antigens 57, 128–137. doi:10.1034/j.1399-0039.2001.057002128.x.

Mohyuddin, A., and Mehdi, S. Q. (2005). HLA analysis of the Parsi (Zoroastrian) population in Pakistan. Tissue Antigens 66, 691–695. doi:10.1111/j.1399-0039.2005.00507.x.

Moscoso, J., Crawford, M. H., Vicario, J. L., Zlojutro, M., Serrano-Vela, J. I., Reguera, R., et al. (2008). HLA genes of Aleutian Islanders living between Alaska (USA) and Kamchatka (Russia) suggest a possible southern Siberia origin. Mol. Immunol. 45, 1018–1026. doi:10.1016/j.molimm.2007.07.024.

Moscoso, J., Seclen, S., Serrano-Vela, J. I., Villena, A., Martinez-Laso, J., Zamora, J., et al. (2006). HLA genes in Lamas Peruvian-Amazonian Amerindians. Mol. Immunol. 43, 1881–1889. doi:10.1016/j.molimm.2005.10.013.

Mullër, C. R., Ehninger, G., and Goldmann, S. F. (2003). Gene and Haplotype Frequencies for the on Over 13,000 German Blood Donors. Hum. Immunol. 2003, 137–151. doi:10.1016/S0198-8859(02)00706-1.

Munkhbat, B., Sato, T., Hagihara, M., Sato, K., Kimura, A., Munkhtuvshin, N., et al. (1997). Molecular analysis of HLA polymorphism in Khoton-Mongolians. Tissue Antigens 50, 124–134. doi:10.1111/j.1399-0039.1997.tb02851.x.

Muro, M., Marín, L., Torío, A., Moya-Quiles, M. R., Minguela, A., Rosique-Roman, J., et al. (2001). HLA polymorphism in the Murcia population (Spain): In the cradle of the archaeologic Iberians. in Human Immunology, 910–921. doi:10.1016/S0198-8859(01)00290-7.

Ogata, S., Shi, L., Matsushita, M., Yu, L., Huang, X. Q., Shi, L., et al. (2007). Polymorphisms of human leucocyte antigen genes in Maonan people in China. Tissue Antigens 69, 154–160. doi:10.1111/j.1399-0039.2006.00698.x.

Okello, E., Beaton, A., Mondo, C. K., Kruszka, P., Kiwanuka, N., Odoi-Adome, R., et al. (2014). Rheumatic heart disease in Uganda: The association between MHC class II HLA DR alleles and disease: A case control study. BMC Cardiovasc. Disord. 14, 2–6. doi:10.1186/1471-2261-14-28.

Paximadis, M., Mathebula, T. Y., Gentle, N. L., Vardas, E., Colvin, M., Gray, C. M., et al. (2012). Human leukocyte antigen class I (A, B, C) and II (DRB1) diversity in the Black and Caucasian South African population. Hum. Immunol. 73, 80–92. doi:10.1016/j.humimm.2011.10.013.

Petzl-Erler, M. L., and Tsuneto, L. T. (2007). “Anthropology/human genetic diversity population reports. Guarani-Kaiowá Amerindians from Mato Grosso do Sul State, Brazil,” in Immunobiology of the Human MHC: Proceedings of the 13th International Histocompatibility Workshop and Conference, Volume I, ed. J. A. Hansen (Seattle: IHWG Press), 642–643.

Piancatelli, D., Canossi, A., Oumhani, K., Aureli, A., Rocco, M., and del Beato, T. (2007). “Anthropology/human genetic diversity population reports. Metalsa from Morocco,” in Immunobiology of the Human MHC: Proceedings of the 13th International Histocompatibility Workshop and Conference, Volume I, ed. J. A. Hansen (Seattle: IHWG Press), 594–595.

Pimtanothai, N., Hurley, C. K., Leke, R., Klitz, W., and Johnson, A. H. (2001). HLA-DR and -DQ polymorphism in Cameroon. Tissue Antigens 58, 1–8. doi:10.1034/j.1399-0039.2001.580101.x.

Pingel, J., Solloch, U. V., Hofmann, J. A., Lange, V., Ehninger, G., and Schmidt, A. H. (2013). High-resolution HLA haplotype frequencies of stem cell donors in Germany with foreign parentage: How can they be used to improve unrelated donor searches? Hum. Immunol. 74, 330–340. doi:10.1016/j.humimm.2012.10.029.

Qin Qin, P., Su, F., Xiao Yan, W., Xing, Z., Meng, P., Chengya, W., et al. (2011). Distribution of human leucocyte antigen-A, -B and -DR alleles and haplotypes at high resolution in the population from Jiangsu province of China. Int. J. Immunogenet. 38, 475–481. doi:10.1111/j.1744-313X.2011.01029.x.

Rajalingam, R., Krausa, P., Shilling, H. G., Stein, J. B., Balamurugan, A., McGinnis, M. D., et al. (2002). Distinctive KIR and HLA diversity in a panel of north Indian Hindus. Immunogenetics 53, 1009–1019. doi:10.1007/s00251-001-0425-5.

Renquin, J., Sanchez-Mazas, A., Halle, L., Rivalland, S., Jaeger, G., Mbayo, K., et al. (2001). HLA class II polymorphism in Aka Pygmies and Bantu Congolese and a reassessment of HLA-DRB1 African diversity. Hum Immunol 58, 211–222.

Rey, D., Parga-Lozano, C., Moscoso, J., Areces, C., Enriquez-De-Salamanca, M., Fernández-Honrado, M., et al. (2013). HLA genetic profile of Mapuche (Araucanian) Amerindians from Chile. Mol. Biol. Rep. 40, 4257–4267. doi:10.1007/s11033-013-2509-3.

Saito, S., Ota, S., Yamada, E., Inoko, H., and Ota, M. (2000). Allele frequencies and haplotypic associations defined by allelic DNA typing at HLA class I and class II loci in the Japanese population. Tissue Antigens 56, 522–529. doi:10.1034/j.1399-0039.2000.560606.x.

Saldanha, N., Spínola, C., Santos, M., Simões, J., Bruges-Armas, J., Brehm, A., et al. (2009). HLA polymorphisms in Forros and Angolares from São Tomé Island (West Africa): Evidence for the Population Origin. J. Genet. Geneal. 5, 76–85.

Sanchez-Mazas, A., Steiner, Q. G., Grundschober, C., and Tiercy, J. M. (2000). The molecular determination of HLA-Cw alleles, in the Mandenka (West Africa) reveals a close genetic relationship between Africans and Europeans. Tissue Antigens 56, 303–312. doi:10.1034/j.1399-0039.2000.560402.x.

Sánchez-Velasco, P., Gómez-Casado, E., Martínez-Laso, J., Moscoso, J., Zamora, J., Lowy, E., et al. (2003). HLA alleles in isolated populations from North Spain : Origin of the Basques and the ancient Iberians. Tissue Antigens 61, 384–392.

Sánchez-Velasco, P., Karadsheh, N. S., García-Martín, A., Ruíz De Alegría, C., and Leyva-Cobián, F. (2001). Molecular analysis of HLA allelic frequencies and haplotypes in Jordanians and comparison with other related populations. Hum. Immunol. 62, 901–909. doi:10.1016/S0198-8859(01)00289-0.

Sánchez-Velasco, P., and Leyva-Cobián, F. (2001). The HLA class I and class II allele frequencies studied at the DNA level in the Svanetian population (Upper Caucasus) and their relationships to Western European populations. Tissue Antigens 58, 223–233. doi:10.1034/j.1399-0039.2001.580402.x.

Santhosh, A., Mustafa, M., Vijayalakshmi, V., Alvares, M., Gumama, O., El Hag, M., et al. (2004). A-B and A-B-DR haplotype frequencies in United Arab Emirates nationals. Emirates Med J 22, 215–9.

Severson, L., Crews, D., and Lang, R. (2007). “Samoans from American Samoan. Anthropology/human genetic diversity population reports,” in Immunobiology of the Human MHC: Proceedings of the 13th International Histocompatibility Workshop and Conference, ed. J. A. Hansen (Seattle: IHWG Press), 623–4.

Shankarkumar, U., Pawar, A., Ghosh, K., Bajpai, S., and Pazare, A. (2010). Human leucocyte antigen class II DRB1 and DQB1 associations in human immunodeficiency virus-infected patients of Mumbai, India. Int. J. Immunogenet. 37, 199–204. doi:10.1111/j.1744-313X.2010.00911.x.

Shi, L., Shi, L., Yao, Y. F., Matsushita, M., Yu, L., Huang, X. Q., et al. (2010). Genetic link among Hani, Bulang and other Southeast Asian populations: Evidence from HLA -A, -B, -C, -DRB1 genes and haplotypes distribution. Int. J. Immunogenet. 37, 467–475. doi:10.1111/j.1744-313X.2010.00949.x.

Silvera, C., Vargas-Alarcon, G., Areces, C., Rey, D., Parga-Lozano, C., Gomez-Prieto, P., et al. (2011). HLA genes in Wayu Amerindians from Colombia. Immunol. Invest. 40, 92–100. doi:10.3109/08820139.2010.517390.

Single, R. M., Meyer, D., Nunes, K., Francisco, R. S., Hünemeier, T., Maiers, M., et al. (2020). Demographic history and selection at HLA loci in Native Americans. PLoS One 15, e0241282.

Spínola, H., Bruges-Armas, J., Middleton, D., and Brehm, A. (2005). HLA polymorphisms in Cabo Verde and Guiné-Bissau inferred from sequence-based typing. Hum. Immunol. 66, 1082–1092. doi:10.1016/j.humimm.2005.09.001.

Steiner, D., Schlaphoff, T., and Borrill, V. (2009). HLA Study in Nigeria. Tissue Antigens 68, 2007.

Sulcebe, G., Sanchez-Mazas, A., Tiercy, J. M., Shyti, E., Mone, I., Ylli, Z., et al. (2009). HLA allele and haplotype frequencies in the Albanian population and their relationship with the other European populations. Int. J. Immunogenet. 36, 337–343. doi:10.1111/j.1744-313X.2009.00868.x.

Suslova, T. A., Burmistrova, A. L., Vavilov, M. N., Chernova, M. S., Khromova, E. B., Belyaeva, S. V., et al. (2012). HLA gene and haplotype frequencies in Russians, Bashkirs and Tatars, living in the Chelyabinsk Region (Russian South Urals). Int. J. Immunogenet. 39, 394–408. doi:doi: 10.1111/j.1744-313X.2012.01117.x HLA.

Tanaka, H., Tokunaga, K., Inoko, H., Tsuji, K., Chimge, N., Jia, J., et al. (1997). “Distribution of HLA-A, B, and DRB1 alleles and haplotypes in Northeast Asia,” in Genetic diversity of HLA. Functional and medical implications, ed. D. Charron (Sèvres: EDK: Medical and Scientific International Publisher).

Tang, J., Naik, E., Costello, C., Karita, E., Rivers, C., Allen, S., et al. (2000). Characteristics of HLA class I and class II polymorphisms in Rwandan women. Exp. Clin. Immunogenet. 17, 185–198. doi:19138.

Tang, J., Penman-Aguilar, A., Lobashevsky, E., Allen, S., Kaslow, R. A., and Zambia-UAB HIV Research Project (2004). HLA-DRB1 and -DQB1 alleles and haplotypes in Zambian couples and their associations with heterosexual transmission of HIV type 1. J. Infect. Dis. 189, 1696–704. doi:10.1086/383280.

Thorsby, E. (2012). The Polynesian gene pool: An early contribution by Amerindians to Easter Island. Philos. Trans. R. Soc. B Biol. Sci. 367, 812–819. doi:10.1098/rstb.2011.0319.

Trachtenberg, E., Vinson, M., Hayes, E., Hsu, Y. M., Houtchens, K., Erlich, H., et al. (2007). HLA class I (A, B, C) and class II (DRB1, DQA1, DQB1, DPB1) alleles and haplotypes in the Han from southern China. Tissue Antigens 70, 455–463. doi:10.1111/j.1399-0039.2007.00932.x.

Vargas-Alarcon, G., Hernandez-Pacheco, G., Zuniga, J., Rodriguez-Perez, J. M., Perez-Hernandez, N., Rangel, C., et al. (2003). Distribution of HLA-B alleles in Mexican Amerindian populations. Immunogenetics 54, 756–760. doi:10.1007/s00251-002-0522-0.

Vargas-Alarcon, G., Moscoso, J., Martinez-Laso, J., Rodriguez-Perez, J. M., Flores-Dominguez, C., Serrano-Vela, J. I., et al. (2007). Origin of Mexican Nahuas (Aztecs) according to HLA genes and their relationships with worldwide populations. Mol. Immunol. 44, 747–755. doi:10.1016/j.molimm.2006.04.014.

Wen, S. H., Lai, M. J., and Yang, K. L. (2008). Human leukocyte antigen-A, -B, and -DRB1 haplotypes of cord blood units in the Tzu Chi Taiwan Cord Blood Bank. Hum. Immunol. 69, 430–436. doi:10.1016/j.humimm.2008.05.012.

Williams, R., Chen, Y. F., Endres, R., Middleton, D., Trucco, M., Williams, J. D., et al. (2009). Molecular variation at the HLA-A, B, C, DRB1, DQA1, and DQB1 loci in full heritage American Indians in Arizona: Private haplotypes and their evolution. Tissue Antigens 74, 520–533. doi:10.1111/j.1399-0039.2009.01381.x.

Yamazaki, A., Yasunami, M., Ofori, M., Horie, H., Kikuchi, M., Helegbe, G., et al. (2011). Human leukocyte antigen class I polymorphisms influence the mild clinical manifestation of Plasmodium falciparum infection in Ghanaian children. Hum. Immunol. 72, 881–888. doi:10.1016/j.humimm.2011.06.007.

Yang, G., Deng, Y. J., Hu, S. N., Wu, D. Y., Li, S. B., Zhu, J., et al. (2006). HLA-A, -B, and -DRB1 polymorphism defined by sequence-based typing of the Han population in Northern China. Tissue Antigens 67, 146–152. doi:10.1111/j.1399-0039.2006.00529.x.

Yuliwulandari, R., Sachrowardi, Q., Nakajima, H., Kashiwase, K., Hirayasu, K., Mabuchi, A., et al. (2010). Association of HLA-A, -B, and -DRB1 with pulmonary tuberculosis in western Javanese Indonesia. Hum. Immunol. 71, 697–701. doi:10.1016/j.humimm.2010.04.005.
